# Correction: *Aegicetus gehennae*, a new late Eocene protocetid (Cetacea, Archaeoceti) from Wadi Al Hitan, Egypt, and the transition to tail-powered swimming in whales

**DOI:** 10.1371/journal.pone.0230596

**Published:** 2020-03-12

**Authors:** The PLOS ONE Staff

## Notice of Republication

This article was republished on February 26, 2020, to correct errors in the order of the figures and in Table 7. The publisher apologizes for this error. Please download this article again to view the correct version. The originally published, uncorrected article and the republished, corrected article are provided here for reference.

## Supporting information

S1 File Originally published, uncorrected article. (PDF)

S2 File Republished, corrected article. (PDF)
