# Supplementary material for: Aegicetus gehennae, a new late Eocene protocetid (Cetacea, Archaeoceti) from Wadi Al Hitan, Egypt, and the transition to tail-powered swimming in whales
Source: PLoS One. 2019 Dec 11;14(12):e0225391. doi: 10.1371/journal.pone.0225391 (PMC6905522; doi:10.1371/journal.pone.0225391)
Supplement: S1 Table — (PDF) [file pone.0225391.s001.pdf]

S1 Table. Online Hyperlinks for Three-Dimensional Images of Protocetid *Aegicetus gehennae* Skeletal Elements (CGM 60584)

Image	Hyperlink	Model ID	Image	Hyperlink	Model ID
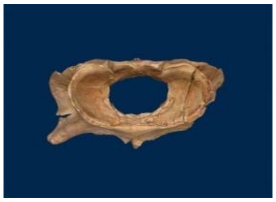	Cervical C1	1348	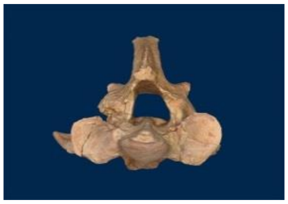	Cervical C2	1350
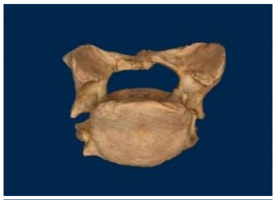	Cervical C3	1351	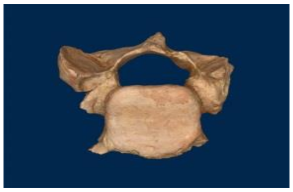	Cervical C4	1352
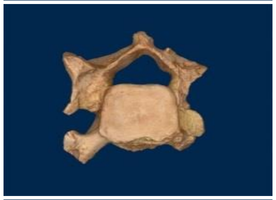	Cervical C5	1353	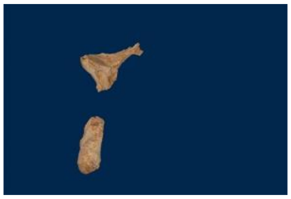	Cervical C6	1354
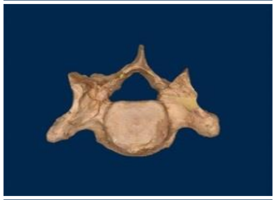	Cervical C7	1355	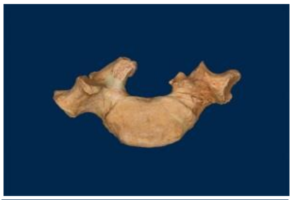	Thoracic T1	1356
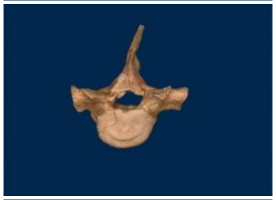	Thoracic T2	1357	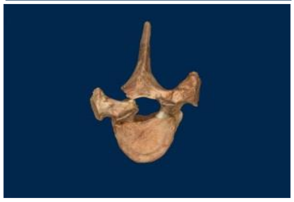	Thoracic T3	1358
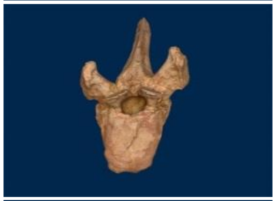	Thoracic T4	1359	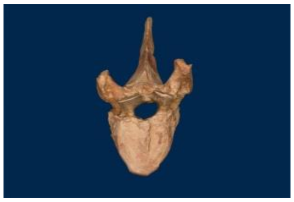	Thoracic T5	1360
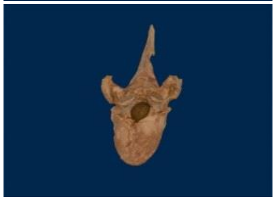	Thoracic T6	1361	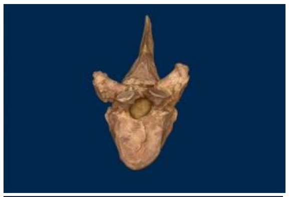	Thoracic T7	1362
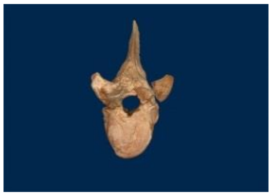	Thoracic T8	1363	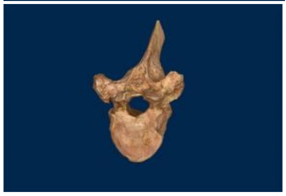	Thoracic T9	1364

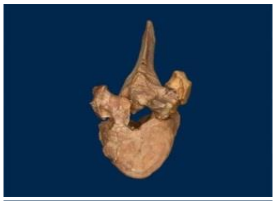	Thoracic T10	1365	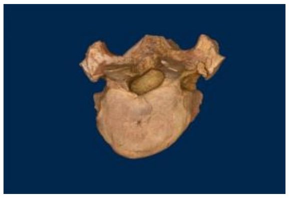	Thoracic T11	1366
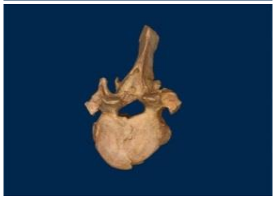	Thoracic T12	1367	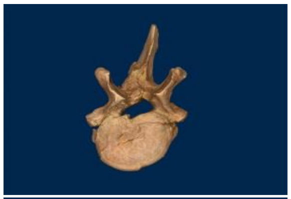	Thoracic T13	1369
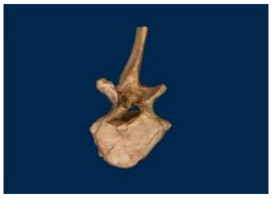	Thoracic T14	1370	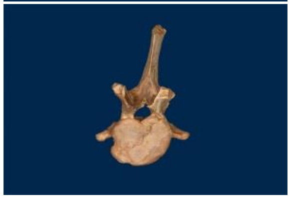	Thoracic T15	1371
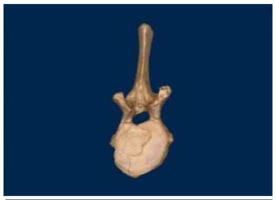	Lumbar L1	1372	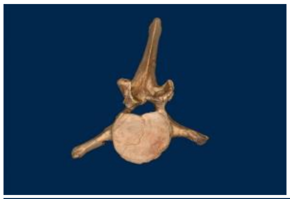	Lumbar L2	1373
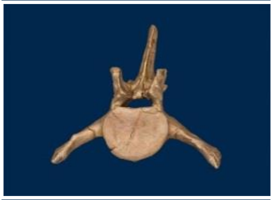	Lumbar L3	1374	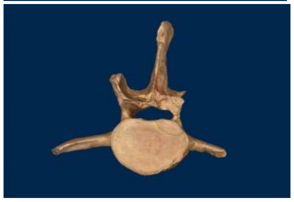	Lumbar L4	1375
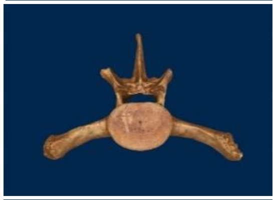	Sacral S1	1376	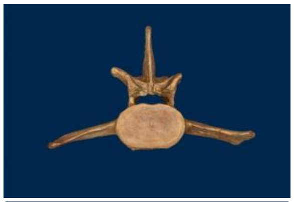	Sacral S2	1377
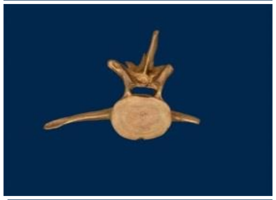	Sacral S3	1378	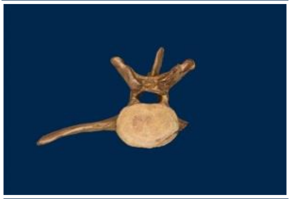	Sacral S4	1379
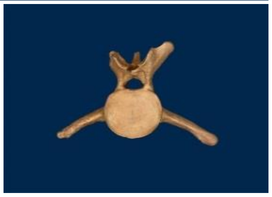	Caudal Ca1	1380	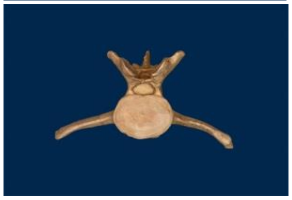	Caudal Ca2	1381
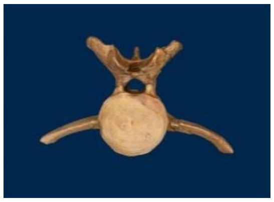	Caudal Ca3	1382	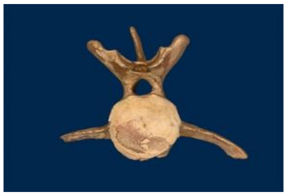	Caudal Ca4	1383

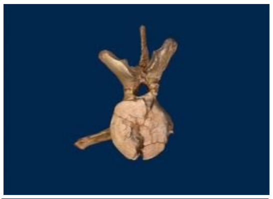

Caudal Ca5 1384

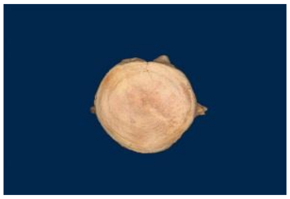

Caudal Ca6 1385

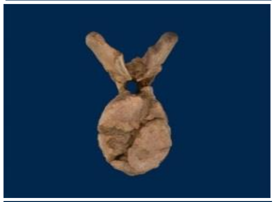

Caudal Ca7 1386

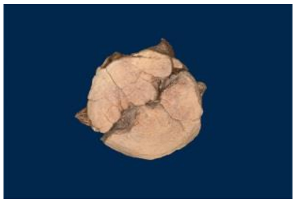

Caudal Ca8 1387

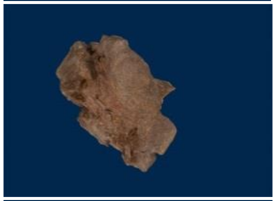

Caudal Ca9 1388

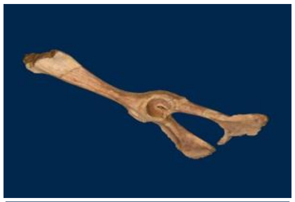

L. innomin. 1390

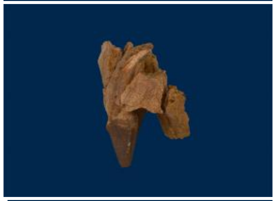

R. maxilla 1394

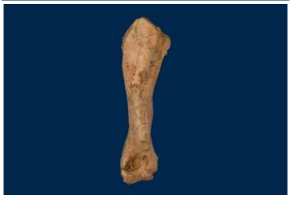

L. humerus 1395

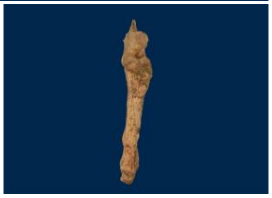

L. ulna 1396

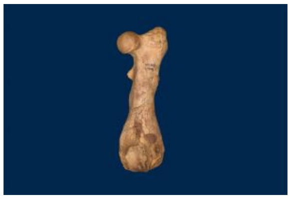

L. femur 1397

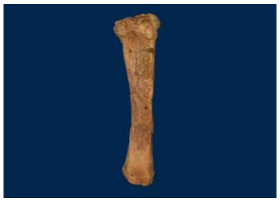

R. tibia 1398
