# Supplementary material for: Correction: Aegicetus gehennae, a new late Eocene protocetid (Cetacea, Archaeoceti) from Wadi Al Hitan, Egypt, and the transition to tail-powered swimming in whales
Source: PLoS One. 2020 Mar 12;15(3):e0230596. doi: 10.1371/journal.pone.0230596 (PMC7067414; doi:10.1371/journal.pone.0230596)
Supplement: S1 File — (PDF) [file pone.0230596.s001.pdf]

PLOS ONE

RESEARCH ARTICLE

# *Aegicetus gehennae*, a new late Eocene protocetid (Cetacea, Archaeoceti) from Wadi Al Hitan, Egypt, and the transition to tail-powered swimming in whales

**Philip D. Gingerich**[1]*, **Mohammed Sameh M. Antar**[2], **Iyad S. Zalmout**[1,3]

**1** Museum of Paleontology, University of Michigan, Ann Arbor, Michigan, United States of America, **2** Department of Geology and Paleontology, Nature Conservation Sector, Egyptian Environmental Affairs Agency, Cairo, Egypt, **3** Department of Paleontology, Saudi Geological Survey, Jeddah, Saudi Arabia

* gingeric@umich.edu

Check for updates

OPEN ACCESS

**Citation:** Gingerich PD, Antar MSM, Zalmout IS (2019) *Aegicetus gehennae*, a new late Eocene protocetid (Cetacea, Archaeoceti) from Wadi Al Hitan, Egypt, and the transition to tail-powered swimming in whales. PLoS ONE 14(12): e0225391. https://doi.org/10.1371/journal.pone.0225391

**Editor:** Thierry Smith, Royal Belgian Institute of Natural Sciences, BELGIUM

**Received:** May 28, 2019

**Accepted:** October 30, 2019

**Published:** December 4, 2019

**Copyright:** © 2019 Gingerich et al. This is an open access article distributed under the terms of the Creative Commons Attribution License, which permits unrestricted use, distribution, and reproduction in any medium, provided the original author and source are credited.

**Data Availability Statement:** All of the data used in this study are presented in the text, figures, tables, and links to online 3D images of original fossils at the University of Michigan's Museum of Palaeontology online repository of fossils in the manuscript and in the supporting information file. The original fossils are conserved in the Egyptian Geological Museum in Cairo.

**Funding:** Research in Egypt was sponsored by the Egyptian Geological Museum, the Egyptian

## Abstract

*Aegicetus gehennae* is a new African protocetid whale based on a partial skull with much of an associated postcranial skeleton. The type specimen, Egyptian Geological Museum, Cairo [CGM] 60584, was found near the base of the early-Priabonian-age (earliest late Eocene) Gehannam Formation of the Wadi Al Hitan World Heritage Site in Egypt. The cranium is distinctive in having ventrally-deflected exoccipitals. The vertebral column is complete from cervical C1 through caudal Ca9, with a vertebral formula of 7:15:4:4:9+, representing, respectively, the number of cervical, thoracic, lumbar, sacral, and caudal vertebrae. CGM 60584 has two more rib-bearing thoracic vertebrae than other known protocetids, and two fewer lumbars. Sacral centra are unfused, and there is no defined auricular surface on the ilium. Thus there was no weight-bearing sacroiliac joint. The sternum is distinctive in being exceptionally broad and flat. The body weight of CGM 60584, a putative male, is estimated to have been about 890 kg in life. Long bones of the fore and hind limbs are shorter than expected for a protocetid of this size. Bones of the manus are similar in length and more robust compared to those of the pes. A log vertebral length profile for CGM 60584 parallels that of middle Eocene *Maiacetus inuus* through the anterior and middle thorax, but more posterior vertebrae are proportionally longer. Vertebral elongation, loss of a sacroiliac articulation, and hind limb reduction indicate that *Aegicetus gehennae* was more fully aquatic and less specialized as a foot-powered swimmer than earlier protocetids. It is doubtful that *A. gehennae* had a tail fluke, and the caudal flattening known for basilosaurids is shorter relative to vertebral column length than flattening associated with a fluke in any modern whale. Late protocetids and basilosaurids had relatively long skeletons, longer than those known earlier and later, and the middle-to-late Eocene transition from foot-powered to tail-powered swimming seemingly involved some form of mid-body-and-tail undulation.

Environmental Affairs Agency, the Egyptian Mineral Resources Authority, the University of Michigan Museum of Paleontology, the U. S. National Science Foundation (NSF OISE0513544, EAR-0920972), and the National Geographic Society (NGS 7226-04). The funders had no role in study design, data collection and analysis, decision to publish, or preparation of the manuscript.

**Competing interests:** The authors have declared that no competing interests exist.

## Introduction

Protocetidae are semiaquatic whales known from middle Eocene strata in Africa, Asia, North America, and South America [1–7]. Most were foot-powered swimmers with hind limbs anchored to the vertebral column through a solid sacrum. Here we describe the first late Eocene protocetid, from the Gehannam Formation of Wadi Al Hitan in Egypt, which represents a transitional stage between more primitive foot-powered swimmers and more derived tail-powered swimmers.

Wadi Al Hitan or 'Valley of Whales' is a UNESCO World Heritage Site in the Western Desert of Egypt (Fig 1). The site measures approximately 17 km by 17 km and is best known for yielding complete skeletons of the Priabonian-age late Eocene basilosaurid archaeocetes *Dorudon atrox* [8] and *Basilosaurus isis* [9–10], and nearly complete skeletons of the protosirenid and dugongid sirenians *Protosiren smithae* and *Eotheroides* spp. [11]. Most are found in the Birket Qarun Formation exposed in the principal valley running through the World Heritage Site.

In 2007 one of us (M. S. M. A.) found a new area in the eastern part of the World Heritage Site with many archaeocete and sirenian skeletons eroding from the Gehannam Formation (Fig 2). Most are basilosaurids and protosirenids, but one partial skeleton of a protocetid, Egyptian Geological Museum, Cairo [CGM] 60583, was found at field site WH2007-031. Later in 2007 a much more complete skeleton of a protocetid, CGM 60584, was found at field site WH-203. These are the first specimens of a protocetid known to have survived into the Priabonian late Eocene. As might be expected, the more complete specimen indicates a taxon that differs in important ways from all geologically older protocetids that were known previously.

Both specimens described here, CGM 60583 from field site WH2007-031, and CGM 60584 from field site WH-203, were collected and studied following protocols outlined in a three-way Memorandum of Understanding dated November 9, 2004. Parties to this agreement were the Egyptian Geological Survey and Mining Authority [EMRA], Cairo; the Egyptian Environmental Affairs Agency [EEAA], Cairo; and the University of Michigan Museum of Paleontology, Ann Arbor, U. S. A.

Museum abbreviations appearing in the text include: CGM, Egyptian Geological Museum, Cairo; GSP-UM, Geological Survey of Pakistan–University of Michigan, Quetta; and UM, University of Michigan Museum of Paleontology, Ann Arbor.

## Geological setting and age

The geological map in Fig 2 shows the outcrop pattern of the five formations exposed in the Wadi Al Hitan World Heritage Site. All strike roughly southwest–northeast and dip at about 1˚ to the northwest.

The oldest and lowest formation is the El Gharaq Formation, named by Iskander [12], which is a nummulitic limestone exposed in the east and southeastern part of the World Heritage Site. It forms the flat plain visible in the field photograph of Fig 3. The El Gharaq Formation corresponds to the uppermost part of the Wadi Rayan Series of Beadnell [13]. It is easily recognizable in the field by the ubiquitous presence of very large nummulites ranging up to 4–5 cm in diameter. Iskander [12] described the upper surface of the El Gharaq Formation at Garet Gehannam as "the plain between the nummulitic limestone and gray-green, gypsiferous, saliferous, fossiliferous shales" of the Ravine Beds of Beadnell [13].

The second formation in the Wadi Al Hitan series is the Gehannam Formation, named by Said [14] for Garet Gehannam, which it surrounds. This is equivalent to Beadnell's 'Ravine Beds' and includes glauconite-rich clays, mudstones, and sandy mudstones interbedded with marls in the lower part, and calcareous sandstone in the upper part. The lower part of the

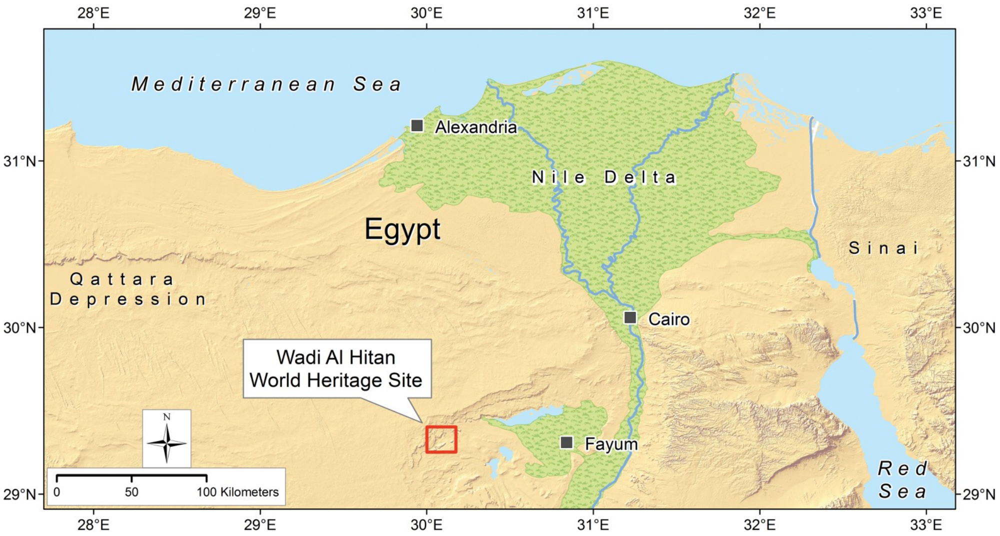


**Fig 1. Wadi Al Hitan World Heritage Site in the western desert of Egypt.** The World Heritage Site (red rectangle) is 150 km southwest of Cairo and 80 km west of Fayum. A more detailed map is shown in Fig 2.

https://doi.org/10.1371/journal.pone.0225391.g001

Gehannam Formation is visible overlying the El Gharaq Formation in the field photograph of Fig 3.

The third, fourth, and fifth formations in the Wadi Al Hitan World Heritage Site are the Birket Qarun, Qasr el-Sagha, and Gebel Qatrani formations, respectively. The Birket Qarun and Qasr el-Sagha formations are both marine and basilosaurid archaeocete-bearing, and the overlying Gebel Qatrani Formation is continental.

Detailed mapping of the El Gharaq and Gehannam formations shows that the two are not strictly conformable. The Gehannam Formation was deposited on an El Gharaq surface of perceptable topographic relief (Fig 4). This disconformity corresponds to the low sea stand Pr-1 of Haq et al. [15] and Hardenbol et al. [16]. Disconformity Pr-1 separates the Bartonian and Priabonian stratigraphic stages and ages (late middle Eocene and late Eocene, respectively). The two protocetid specimens described here, CGM 60583 and CGM 60584, were found several meters above the El Gharaq to Gehannam formation boundary, above disconformity Pr-1. By this criterion both are earliest Priabonian and earliest late Eocene in age.

Stratigraphic section S340 of Strougo et al. [18] spanning the Bartonian–Priabonian transition was sampled a few hundred meters southeast of locality WH-203 on the map in Fig 2. In section S340 the planktonic foramineran *Globigerinatheka semiinvoluta*, characteristic of planktonic foraminiferal zones P15 and E 14, made its first appearance 19 meters above the base of the section and 14 meters above the base of the Gehannam Formation. The calcareous nannofossil *Chiasmolithus oamaruensis* made its first appearance 13 meters above the base of the section and 8 meters above the base of the Gehannam Formation. These two appearances in section S340 bracket the principal global stratotype section and point (GSSP) proposed to mark the base of the Priabonian stage and age, and the base of the late Eocene [19]. The two protocetid specimens described here, CGM 60583 and CGM 60584, appear to have been found several meters below the first appearances of *Globigerinatheka semiinvoluta* and *Chiasmolithus oamaruensis*. By this criterion both specimens could be considered latest Bartonian and latest middle Eocene in age.

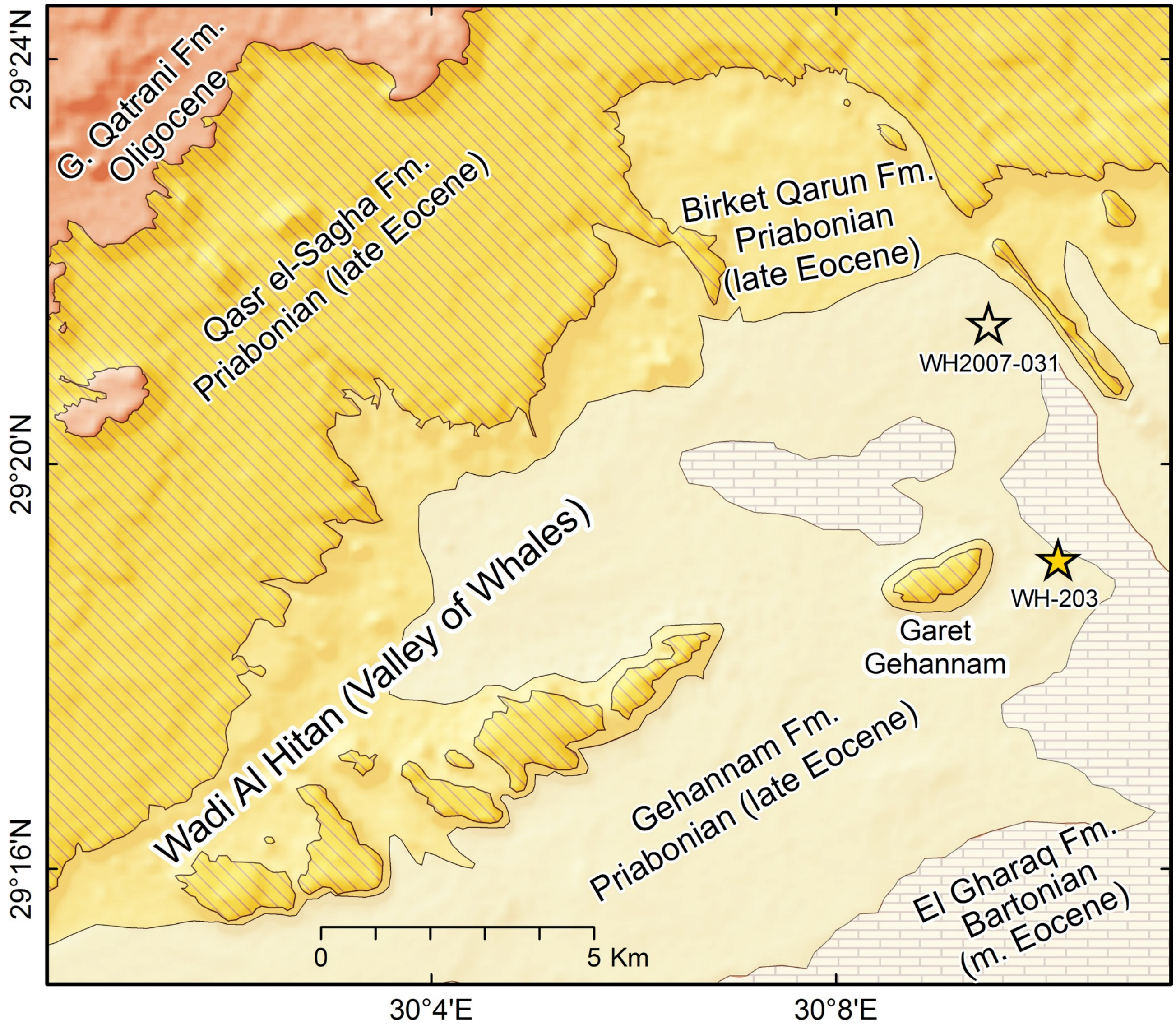


**Fig 2. Geological map of the Wadi Al Hitan World Heritage Site.** Strata are nearly flat-lying. Stars show the type locality of *Aegicetus gehennae* (WH-203; CGM 60584) east of Garet Gehannam and the locality yielding a referred specimen (WH2007-031; CGM 60583) north of Garet Gehannam. Both are in glauconite-rich beds in the lower part of the Gehannam Formation of early Priabonian age.

https://doi.org/10.1371/journal.pone.0225391.g002

It is impossible at present to reconcile a micropaleontological assessment of age with the evidence from global sea level change. Placing more weight on the latter, we conclude that the age of CGM 60583 and CGM 60584 is earliest Priabonian (earliest late Eocene).

## Nomenclatural acts

The electronic edition of this article conforms to the requirements of the amended International Code of Zoological Nomenclature, and hence the new names contained herein are available under that Code from the electronic edition of this article. This published work and the nomenclatural acts it contains have been registered in ZooBank, the online registration system for the ICZN. The ZooBank LSIDs (Life Science Identifiers) can be resolved and the associated information viewed through any standard web browser by appending the LSID to the prefix

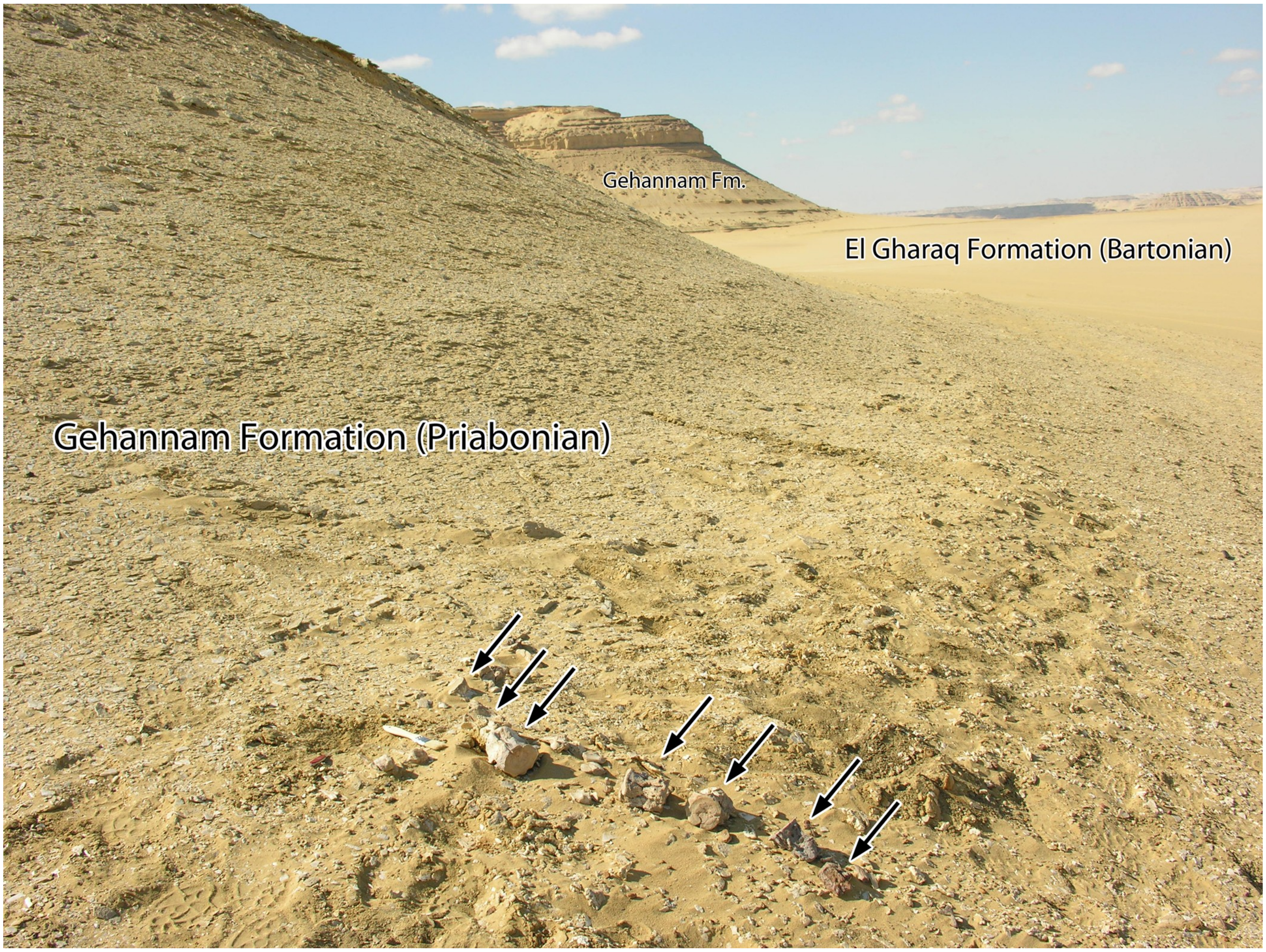


**Fig 3. Type locality of *Aegicetus gehennae* (WH-203) in the Wadi Al Hitan World Heritage Site, Egypt.** Caudal vertebrae aligned in the foreground (arrows; brush for scale) show a series of seven caudal vertebrae of CGM 60584 as they were exposed before excavation. The specimen was found weathering out of indurated glauconite near the base of the Gehannam Formation of late Eocene (Priabonian) age. Plain in the right side of the photograph is hard nummulitic limestone of the El Gharaq Formation of middle Eocene (Bartonian) age.

https://doi.org/10.1371/journal.pone.0225391.g003

"http://zoobank.org/". The LSID for this publication is: urn:lsid:zoobank.org:pub:2-DBF0955A00140D99167485DF2A0B5ED. The electronic edition of this work was published in a journal with an ISSN, has been archived, and is available from the following digital repositories: PubMed Central and LOCKSS.

## Systematic paleontology

**Mammalia** Linnaeus, 1758 [20]

**Cetacea** Brisson, 1762 [21]

**Archaeoceti** Flower, 1883 [22]

**Protocetidae** Stromer, 1908 [23]

**Georgiacetinae** Gingerich et al., 2005 [24]

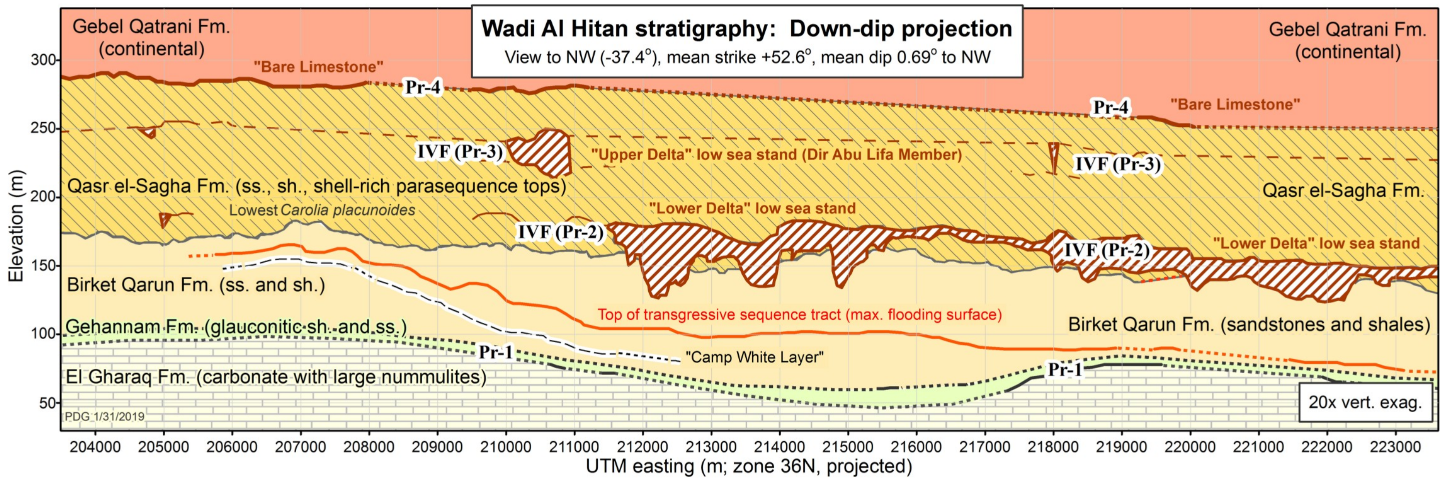


**Fig 4. Stratigraphic cross-section of geological formations exposed in the Wadi Al Hitan World Heritage Site.** Five geological formations are shown, in stratigraphic succession: El Gharaq, Gehannam, Birket Qarun, Qasr el-Sagha, and Gebel Qatrani. View is to the northwest. Formation contacts and incised valley fills (IVF) shown as solid lines were mapped on the ground in three dimensions (UTM easting, UTM northing, and elevation above sea level) using GPS. Dotted lines are interpolated. Average strike and dip were computed by least squares on three-dimensional bedding traces. Lowstand disconformities spanning the Priabonian stage are Pr-1 through Pr-4 [15–16]. Note the disconformity (Pr-1) separating the El Gharaq and Gehannam formations. Horizontal axis is about 20 km, vertical axis is about 300 m, and vertical exaggeration is 20x. Reprinted from [17] under a CC BY 4.0 license, with permission from the Geological Society of America.

https://doi.org/10.1371/journal.pone.0225391.g004

Georgiacetines, like protocetines, have generalized protocetid skulls retaining three incisors in the premaxilla and three molars in the maxilla. Posterior thoracic, lumbar, sacral and proximal caudal vertebrae of georgiacetines are longer relative to anterior thoracic vertebrae than are those of protocetines (see below). Georgiacetines generally have independent sacral vertebrae, with the first bearing auricular processes that do not articulate with ilia of the innominates. In specimens where both are known, the manus is larger than the pes, but both are small in relation to skeletal size compared to the manus and pes of protocetines (see below).

Pappocetinae McLeod and Barnes, 2008 [25] is a subjective junior synonym of Georgiacetinae. Genera included in each subfamily of Protocetidae are listed in a table below (see Discussion). The classification here is generally consistent with most recent phylogenetic analyses of Eocene cetaceans [2, 6–7].

Genus ***Aegicetus*** gen. nov.

urn:lsid:zoobank.org:act:940A5E50-6DD8-48A7-9792-E3CFD2D42EBD

**Type species.** *Aegicetus gehennae* sp. nov.

**Diagnosis.** *Aegicetus* differs from all protocetids for which the cranium is known in having a relatively narrow cranium with short and downwardly-deflected exoccipital processes on the posterior surface of the braincase (see illustration in Discussion).

*Aegicetus* differs from *Pappocetus* [26–29] in being substantially smaller, with cheek teeth about 72–80% as long and a femoral head about 78% in diameter. The metacone on upper $P^{3-4}$ is much better developed in *Aegicetus* than in *Pappocetus*. *Aegicetus* further differs from *Pappocetus* in lacking fusion of left and right dentaries at the mandibular symphysis. The proximal width of the femur (including the femoral head) is a smaller proportion of femoral shaft width (2.3x compared to 2.9x in *Pappocetus*). *Aegicetus* also has a narrower acetabular notch on the innominate.

*Aegicetus* has teeth similar in length and width to those of *Babiacetus* [30–31], but differs from this genus in having higher-crowned cheek teeth. *Aegicetus* retains a more distinct metacone on $P^{3-4}$, and retains larger protocone lobes on $P^3$–$M^3$ than are seen in *Babiacetus*. *Aegicetus* further differs from *Babiacetus* in lacking fusion of left and right dentaries at the mandibular symphysis.

*Aegicetus* is similar to *Georgiacetus* [2, 32] in size, in retaining an open mandibular symphysis, in having a double-rooted $P^1$, and in having relatively long posterior lumbar, sacral, and anterior caudal vertebrae. *Aegicetus* differs from *Georgiacetus* in the narrowness of the cranium and in the orientation of the exoccipital processes (see Discussion). Cheek teeth are very similar, but *Aegicetus* differs in having a lower crowned $P^2$, and an anteroposteriorly narrower protocone lobe on $P^4$. The innominate of *Aegicetus* differs from that of *Georgiacetus* in having: (a) a longitudinal spine on the lateral surface of the ilium, which forms the lower border of a distinctive gluteal fossa; (b) a concave anteromedial surface of the ilium possibly related to connective-tissue attachment to an auricular process of the sacrum; (c) a more gracile ramus of the ischium; (d) a more robust ramus of the pubis; and (e) an anteroposteriorly shorter and dorsoventrally deeper pubic symphysis.

*Aegicetus* differs from the type specimen of *Natchitochia* [6], known from 13 associated vertebrae and three partial ribs, in being substantially smaller and in lacking the synarthrotic connection of the sacrum to the innominates reported for *Natchitochia* [33]. *Aegicetus* differs from the referred specimen of *Natchitochia* in lacking any remnant of pleurapophyseal articulation between successive sacral vertebrae [33], and in lacking the expansion and auricular surface of the proximal ilium. The proximal width of the femur (including the femoral head) is a smaller proportion of femoral shaft width (2.3x compared to 2.8x in *Natchitochia*; measurements from Fig 7 in Uhen [33]).

*Aegicetus* is similar to *Carolinacetus* [34] in vertebral size, and both retain an unfused mandibular symphysis. *Aegicetus* differs from *Carolinacetus* in having a narrower cranium (21.4 cm versus about 28.4 cm in width, measured across lateral margins of the exoccipitals) and it differs in the orientation of exoccipital processes (see Discussion). *Aegicetus* does not have the posterodorsal tongue of the petrosal exposed between the squamosal and exoccipital reported for *Carolinacetus*.

*Crenatocetus* [25] is based on associated left and right partial dentaries and lower cheek teeth of a late middle Eocene georgiacetine from North Carolina. Judging from comparable lower premolar and molar lengths, *Aegicetus* is similar in size to *Crenatocetus*. *Aegicetus* differs in having a broader $P_4$ (18.3 mm compared to 15.0 mm; a proportional difference of 1.22) and a broader $M_2$ (18.1 mm compared to 12.0; a proportional difference of 1.51).

**Etymology.** *Aegis*, shield or protection, and *cetus*, whale (Latin, masculine). Name refers to the distinctive sternum of *Aegicetus*, which forms a broad thoracic shield.

***Aegicetus gehennae*** sp. nov.

urn:lsid:zoobank.org:act:1200478C-70BC-4432-AE47-DB8DDAC0B55C

Figs 6–18, 22

**Holotype.** Egyptian Geological Museum, Cairo [CGM] 60584, partial skeleton with a partial cranium, partial dentition, much of the vertebral column, a nearly complete rib cage, much of the forelimb, and much of the hind limb. The field locality for the type specimen is WH-203 (Figs 2 and 3; at 29.31770˚ N latitude and 30.16994˚ E longitude). Casts of the type specimen are archived as University of Michigan Museum of Paleontology 118210. Links to three-

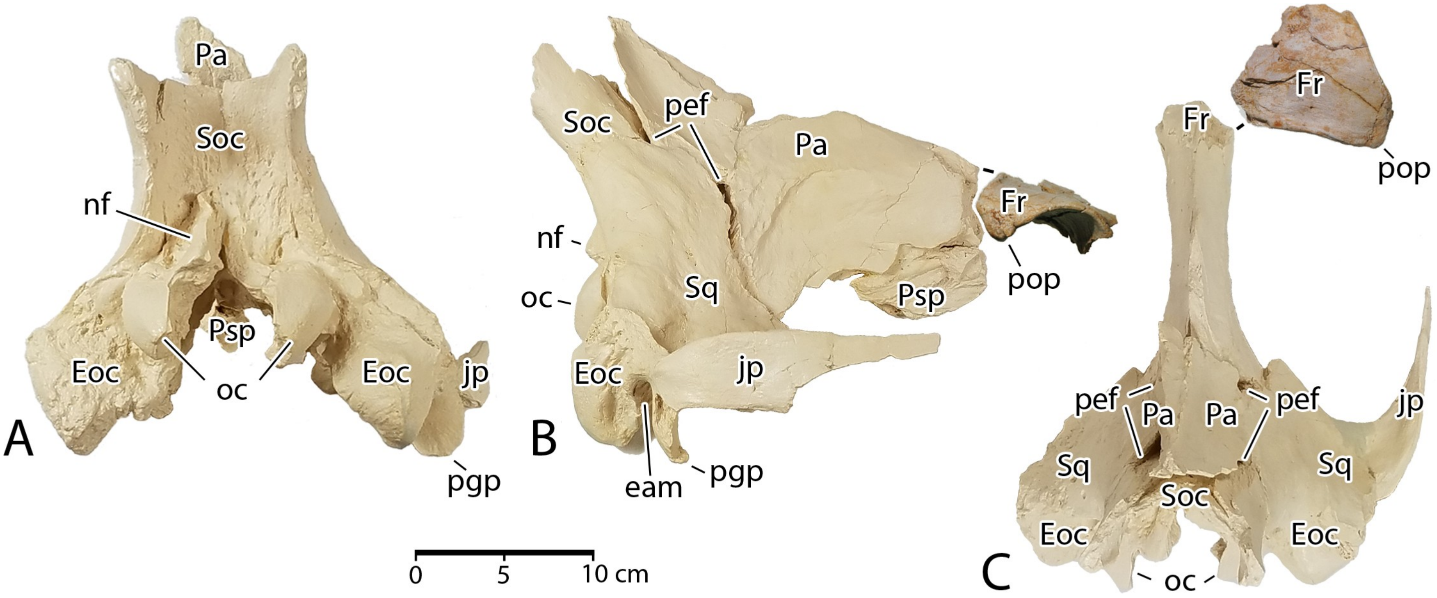


**Fig 7. Cranial elements of *Aegicetus gehennae* type specimen (CGM 60584).** A–C, cranium in posterior, right lateral, and dorsal view. The braincase illustrated here was reassembled using casts of the original bones. Abbreviations: *eam*, external auditory meatus; *Eoc*, exoccipital; *Fr*, frontal; *jp*, jugal process of squamosal; *nf*, nuchal flange of the supraoccipital; *oc*, occipital condyle; *Pa*, parietal; *pef*, parietal emissary foramen; *pgp*, postglenoid process; *pop*, postorbital process; *Psp*, presphenoid; *Soc*, supraoccipital; *Sq*, squamosal.

https://doi.org/10.1371/journal.pone.0225391.g007

dimensional images of vertebrae and other elements of the type skeleton are provided here in Supporting Information (S1 Table).

**Referred specimen.** Egyptian Geological Museum, Cairo [CGM] 60583, an associated but poorly preserved femur and tibia. The field locality for the referred specimen is WH2007-031 (Fig 2; at 29.35658˚ N latitude and 30.15846˚ E longitude). Casts of the referred specimen are archived as University of Michigan Museum of Paleontology 118209.

**Diagnosis.** As for the genus. *A. gehennae* is the only species of *Aegicetus* known at present.

**Type locality and horizon.** Wadi Al Hitan in western Fayum Province, Egypt (see Figs 1 and 2; type locality is at 29.31770˚ N latitude and 30.16994˚ E longitude). The type and referred specimen came from the lower part of the Gehannam Formation of earliest Priabonian age (earliest late Eocene).

**Etymology.** Species name is formed from *gehenna* (Latin), hell (genitive *gehennae*). Named for the type locality, Garet Gehannam (or Gehennam) in the eastern part of Wadi Al Hitan (Fig 2). Garet Gehannam was named for the extremely hot summer temperatures of the surrounding desert.

**Description.** A skeletal map for the type specimen of *Aegicetus gehennae*, CGM 60584, is shown in Fig 5. The cranium is not complete. It is followed by part or all of 39 successive vertebrae. The vertebral formula of cervical–thoracic–lumbar–sacral–caudal vertebrae is 7–15–4–4–9+. The last of the preserved caudals is large and there were probably 11 or 12 additional caudal vertebrae. The rib cage and sternum are nearly complete. The right forelimb and hand are more complete than the left forelimb and hand. The left hind limb and foot are more complete than the right hind limb and foot.

The number of elements of CGM 60584 that are well preserved (95) and the number of elements that are partially preserved (17) can be compared to the total number expected (211), which yields a minimum estimate of skeletal completeness of 112 / 211 = 0.53 or 53%. Alternatively, if we consider the bilateral symmetry of a skeleton, we can compare the number of

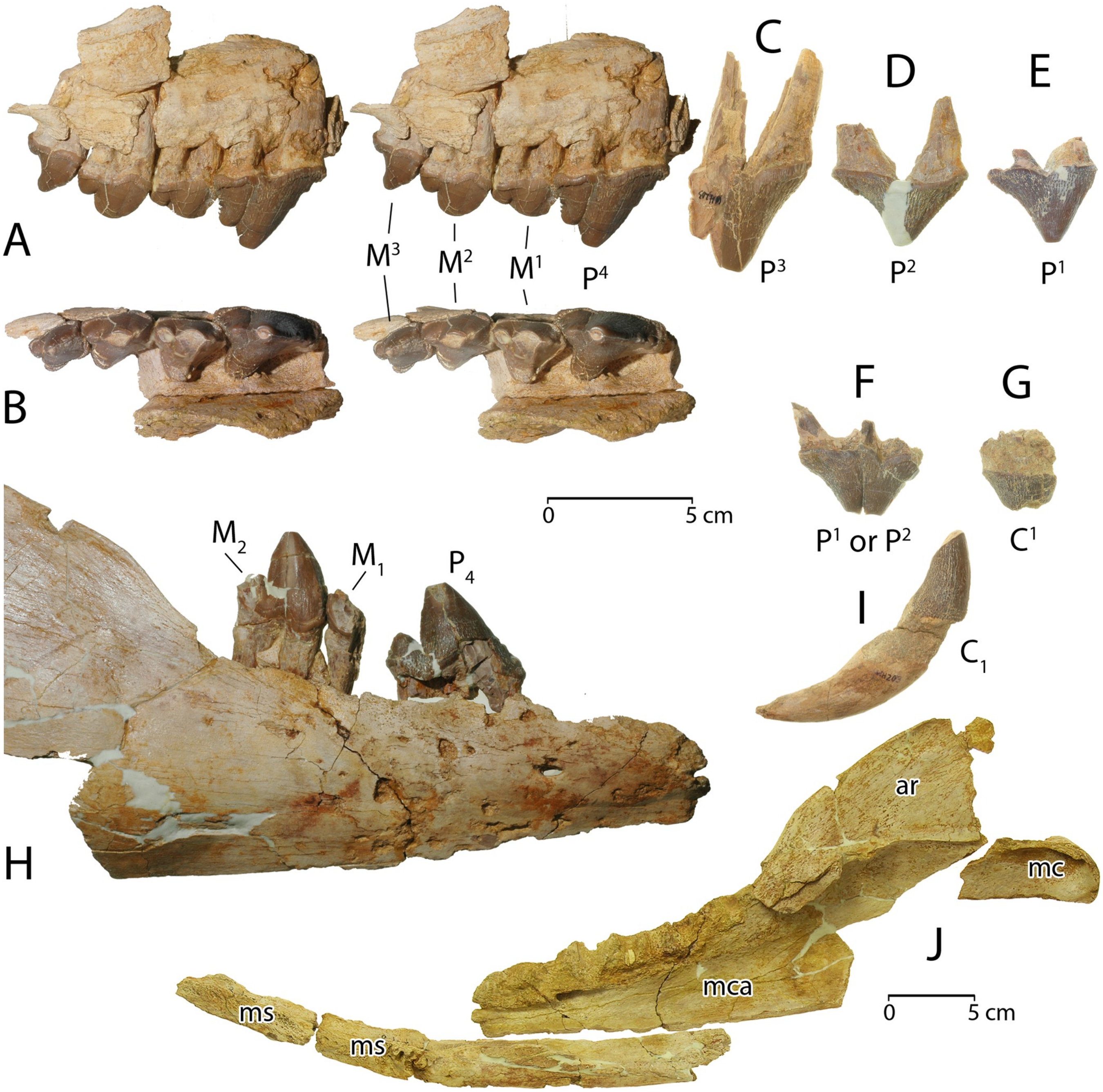


**Fig 6. Dental elements of *Aegicetus gehennae* type specimen (CGM 60584).** A, right maxilla with upper cheek teeth $P^4$ and $M^{1-3}$ (stereophotograph) in right lateral view. B, right maxilla with $P^4$ and $M^{1-3}$ (stereophotograph) in occlusal view. C, right $P^3$ in right lateral view. D, right $P^2$ in right lateral view. E, right $P^1$ in right lateral view. F, teratological left $P^1$ or $P^2$ in left lateral view. G, right $C^1$ in right lateral view. H, partial right dentary with lower cheek teeth $P_4$, $M_1$ talonid, and $M_2$ in right lateral view. I, right lower canine $C_1$ in right lateral view. J. right dentary as preserved without teeth, in four pieces, in left medial view (reduced scale). Abbreviations: *ar*, ascending ramus; *mc*, mandibular condyle; *mca*, mandibular canal; *ms*, mandibular symphysis.

https://doi.org/10.1371/journal.pone.0225391.g006

elements observed and expected after reflecting left elements to the right side. This yields 47 midline elements plus 15 + 14 + 15 = 44 non-midline elements for a total of 91 observed, compared to a total of 59 + 15 + 31 + 30 = 135 expected. By this calculation the ratio of observed to expected is 91 / 135 = 0.67 or 67%.

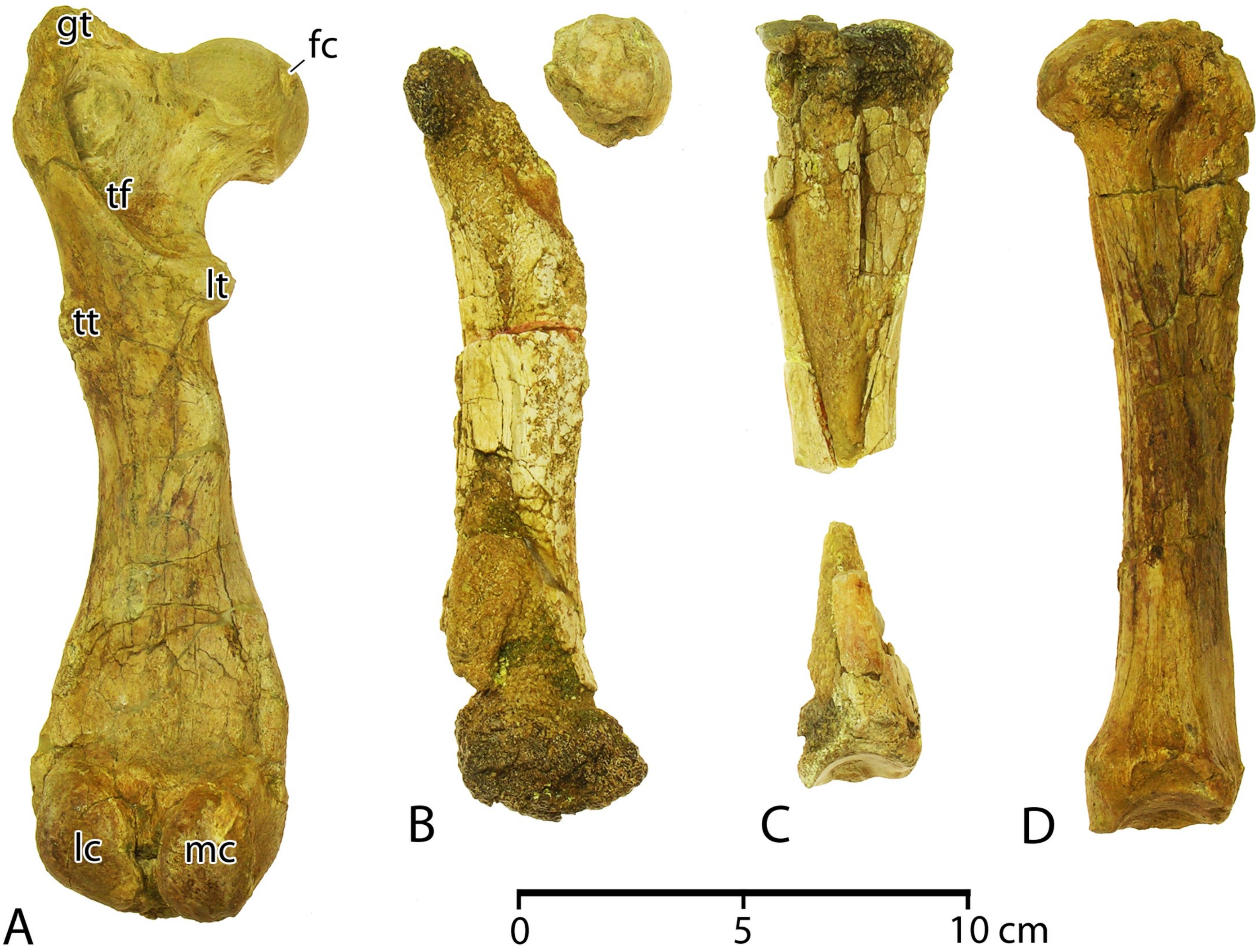


**Fig 18. Sexual dimorphism in *Aegicetus gehennae*.** A, left femur of CGM 60584 interpreted as male, in posterior view. B, weathered left femur of CGM 60583 interpreted as female, in posterior view. C, right tibia of CGM 60583 interpreted as female, in lateral view. D, right tibia of CGM 60584 interpreted as male, in lateral view. Elements interpreted as male average 11% larger in linear dimensions than elements interpreted as female. Abbreviations: *fc*, fovea capitis femoris; *gt*, greater trochanter; *lc*, lateral condyle; *lt*, lesser tuberosity; *mc*, medial condyle; *tf*, trochanteric fossa; *tt*, third trochanter.

https://doi.org/10.1371/journal.pone.0225391.g018

The cranium of CGM 60584 is represented by some thirteen pieces that appear to have been broken or disarticulated and separated to some degree before burial. Substantial pieces were not recovered. Breaks between pieces are not fresh but appear to have been damaged before burial. This is not surprising when we consider that the enclosing sediment is glauconite-rich. Glauconite accumulates slowly during the sediment-starved initial stage of a marine transgression. The cause of the breakage is not known. Vertebrae T14, T15, and S2 have obliquely truncated neural spines or transverse processes that were possibly gouged by the teeth of a predator or scavenger.

Many bones of the skeleton have surfaces bearing circular 3–5 mm scars produced by epibionts. These scars average 2.5–3 mm in diameter, have multiple radiating sulci, and resemble the attachment scars produced by encrusting barnacles [35]. They bear some similarity to the trace fossils *Centrichnus concentricus* [36], and *Anellusichnus undulatus* [37]. The presence of

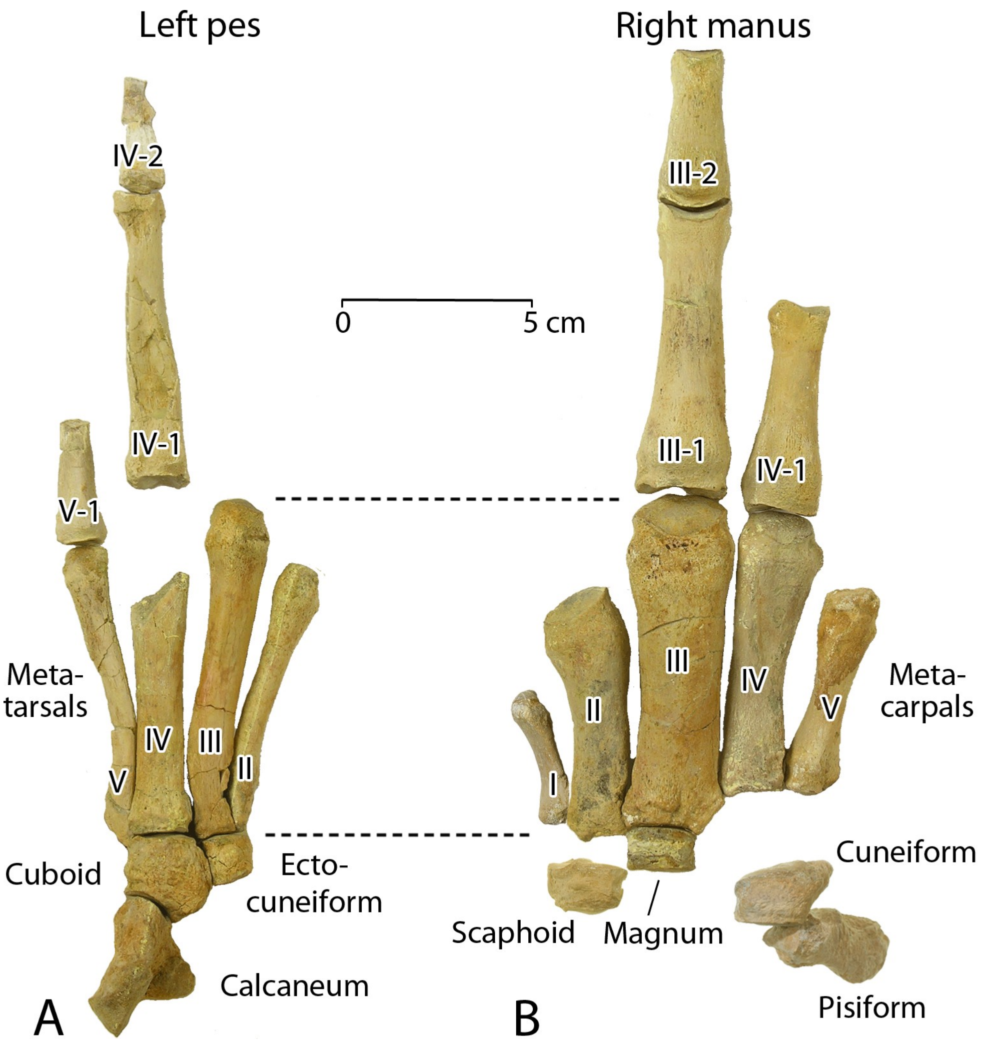


**Fig 22. Pes and manus of the *Aegicetus gehennae* type specimen (CGM 60584) compared at the same scale.** A, left pes in dorsal view. B, right manus in dorsal view. The longest metatarsals are approximately the same length as the longest metacarpals (dashed lines), and the latter are much more robust. Earlier protocetids have a pes substantially longer than the manus, but here the manus and pes are almost equal in length.

https://doi.org/10.1371/journal.pone.0225391.g022

such scars indicates again that sediment accumulation was slow, and bones of the cranium were clearly exposed on the sea floor for a substantial interval of time before burial.

**Dentition**. CGM 60584 includes a rostrum with roots or alveoli for several teeth. The left side preserves alveoli for single-rooted $I^{1-3}$ and a single-rooted $C^1$. The right side preserves alveoli for single-rooted $I^{1-3}$, a single-rooted $C^1$, and the anterior root and posterior alveolus for a double-rooted $P^1$. Isolated crowns are present for right $C^1$ and right $P^{1-3}$ (Fig 6). The crown of right $C^1$ is single-rooted and blunted by breakage and wear (Fig 6G).

Right $P^1$ is double-rooted and relatively long and narrow for a tooth at this position (Fig 6E). It has a simple crown with a single paracone cusp at the apex, and an anterior carina straighter and steeper than the posterior carina. There are no accessory cusps. The crown is surrounded at the base, labially and lingually, by a narrow cingulum. Enamel is smooth near the apex of the paracone but crenulated over much of the crown. Right $P^2$ is similar to $P^1$ but longer mesiodistally (Fig 6D). It differs too in having two small cuspules near the base of the

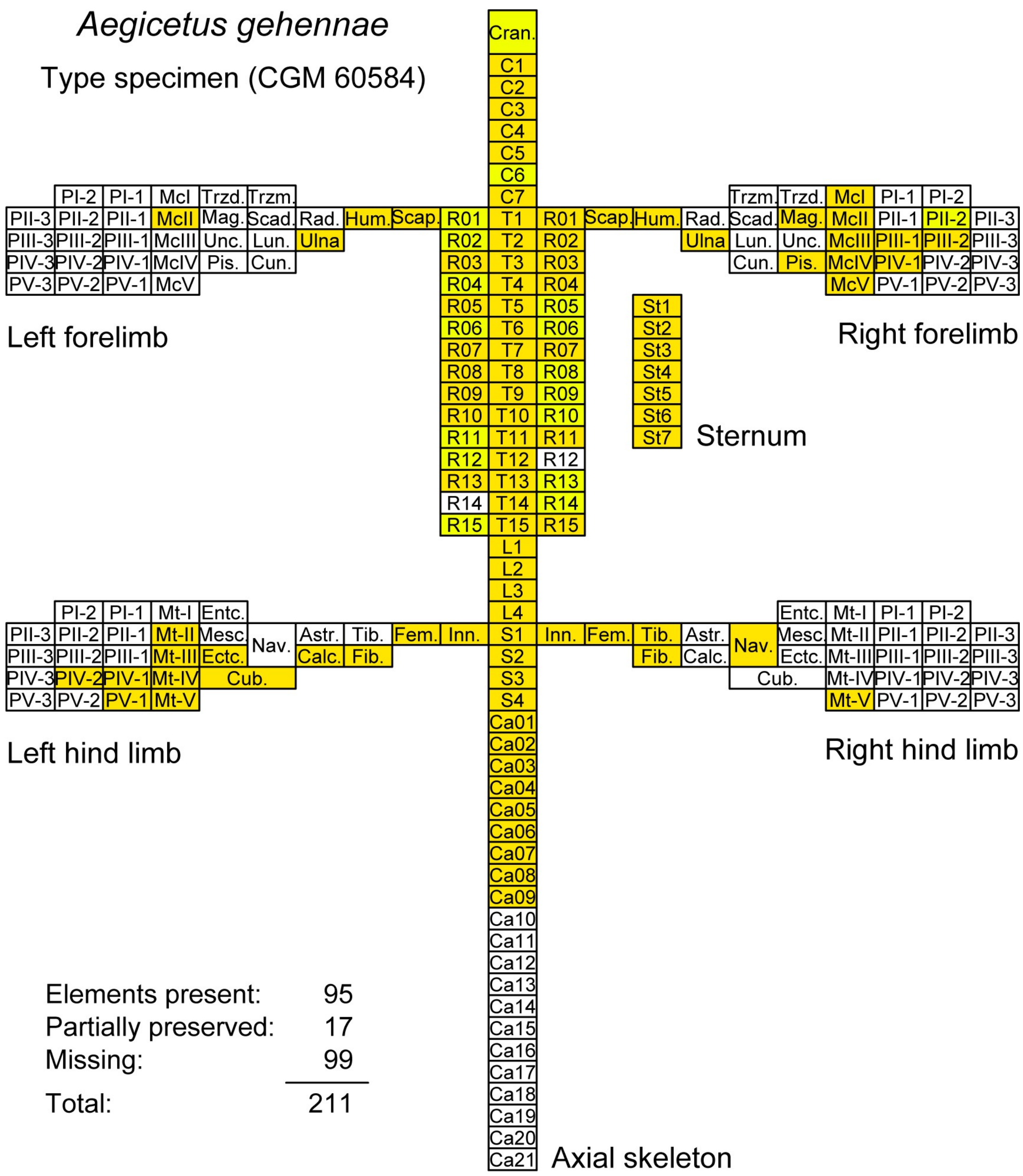


**Fig 5. Skeletal map for the *Aegicetus gehennae* type specimen (CGM 60584).** Elements include the axial skeleton (cranium, cervical, thoracic, lumbar, sacral, and caudal vertebrae, left and right ribs, and sternum), the left and right forelimbs, and the left and right hind limbs. Darker color indicates skeletal elements present in CGM 60584 (95 elements). Lighter color indicates elements partially preserved (17 elements). Open rectangles represent missing elements (99 elements). Missing elements are known or expected based on comparison with the skeleton of *Maiacetus inuus* [5].

https://doi.org/10.1371/journal.pone.0225391.g005

posterior carina. Right $C^1$, $P^1$, and $P^2$ all display some erosion of dentine at the gum line above the base of the crown.

Right $P^3$ is double-rooted (Fig 6C). Part of the crown and posterior root are broken. $P^3$ has a well developed metacone posterior to the paracone, and a posterolingual expansion at the base of the crown similar to the protocone lobe on following teeth. There are no accessory cusps. The crown is surrounded at the base by a narrow cingulum, and enamel is again smooth

near the apex but crenulated over much of the crown. $P^3$ has a conspicuous wear facet perforating the enamel of the lingual cingulum where the apex of the $P_3$ protoconid occluded. $P^3$ and following teeth show none of the dentinal erosion seen on more anterior teeth.

CGM 60584 includes a partial right maxilla with crowns of $P^4$ and $M^{1-3}$ (Fig 6A and 6B links to three-dimensional images of the maxilla are provided here in Supporting Information [S1 Table]). $P^4$ is substantially larger than the following molars, with a robust projecting paracone and smaller metacone near the labial margin of the crown. It has a prominent protocone lobe projecting lingually and slightly posteriorly at the base of the crown. This is narrower anteroposteriorly than the protocone lobe on $P^3$. There is an anterior carina projecting forward from the paracone of $P^4$ with three cusp-like swellings near and at the base of the crown. There is a posterior carina projecting backward from the metacone with several small serrations. The crown of $P^4$ is surrounded by a basal cingulum except anterolingually where occlusion of the protoconid of $P_4$ removed this. Enamel on the surface of the crown is slightly crenulated, but again smoother near the apex of the paracone.

$M^1$ is a triangular tooth with a prominent paracone and smaller metacone posterior to this near the labial or buccal margin of the crown. Both cusps are worn obliquely, removing a substantial part of each. There is a prominent protocone lobe projecting lingually at the base of the crown. $M^1$ has an anterior carina projecting forward from the paracone with a small accessory cusp in the middle of this crest. The crown of $M^1$ is completely surrounded by a basal cingulum. $M^2$ is similar to $M^1$ but differs in having a smaller metacone posterior to the paracone, in having the accessory cusp higher on the anterior carina, and in having a less prominent protocone lobe projecting both lingually and posteriorly relative to the paracone. There is an anterolingual swelling at the base of the crown that makes the crown more rectangular than triangular in occlusal outline. $M^3$ differs from $M^2$ in being smaller and in having a single cusp, the paracone, on the buccal margin of the crown. The metacone is reduced to a swelling near the base of the crown. The protocone lobe is shorter and more posteriorly directed relative to the paracone, and the anterolingual swelling at the base of the crown is more prominent. The latter traits make the crown of $M^3$ fully rectangular. All three upper molars have very slightly crenulated enamel.

There is one left upper tooth (Fig 6F) of uncertain homology. It is developmentally anomalous, seemingly three-rooted, and appears to represent crowns of two upper premolars, $P^1$ and/or $P^2$, fused in duplicate just below the apices of their separate paracones. Measurements for upper teeth are included in Table 1.

The most anterior lower tooth is a right lower canine, $C_1$, which has a simple curving conical crown and a relatively long root (Fig 6I). The apex has been removed by wear, and a narrow band of wear continues down the posterior surface of the crown. There is a distinct carina of enamel running down the anterolingual surface of the crown. Enamel on $C_1$ is crenulated. Dentine is deeply eroded at the gum line below the base of the crown

Right $P_4$, positioned in the right dentary (Fig 6H), has a relatively long narrow crown, with a single high protoconid and a smaller basal talonid cusp. The apex of the protoconid has the enamel perforated by wear. Polished and striated wear facets continue down the labial side of the crown. These match wear on the anterolingual portion of the crown of $P^4$. The anterior carina on $P_4$ is nearly straight, with small cuspules near the base of the crown. The basal talonid cusp is blunt, with wear on the labial surface matching that on the lingual side of the $P^4$ metacone. The only lower tooth from the left side is a partial crown of left $P_4$. This is less complete but otherwise similar to right $P_4$.

$M_1$ is represented by its talonid in a piece of dentary that also has a complete $M_2$ (Fig 6H). Little can be said about the $M_1$ talonid except that it is a single cusp heavily worn on the labial or buccal surface. $M_2$ has a high straight protoconid portion of the crown, which is worn on

**Table 1. Measurements of teeth preserved in CGM 60584, type specimen of *Aegicetus gehennae*.**

| Upper Dentition | | | | Lower Dentition | | | |
|---|---|---|---|---|---|---|---|
| **Position** | **Length** | **Width** | **Height** | **Position** | **Length** | **Width** | **Height** |
| $I^1$ | — | — | — | $I_1$ | — | — | — |
| $I^2$ | — | — | — | $I_2$ | — | — | — |
| $I^3$ | — | — | — | $I_3$ | — | — | — |
| $C^1$ | 27.0 | 16.4* | — | $C_1$ | 20.7 | 16.0 | 33.6* |
| $P^1$ | 39.0 | 15.7 | 27.7 | $P_1$ | — | — | — |
| $P^2$ | 49.0 | 15.2* | 25.1 | $P_2$ | — | — | — |
| $P^3$ | 42.1 | 25.2 | 31.7* | $P_3$ | — | — | — |
| $P^4$ | 40.0 | 26.3 | 27.6 | $P_4$ | 49.8 | 18.3 | 36.1* |
| $M^1$ | 25.3 | 24.5 | — | $M_1$ | — | — | — |
| $M^2$ | 26.3 | 25.0 | — | $M_2$ | 33.5 | 18.1 | 35.5 |
| $M^3$ | 21.5 | 16.8 | 17.8 | $M_3$ | — | — | — |

Measurements for tooth crowns of upper and lower incisors (I), canines (C), premolars (P), and molars (M) are given in mm. Estimates are marked with an asterisk.

https://doi.org/10.1371/journal.pone.0225391.t001

the buccal surface where it occluded with the lingual surface of $M^2$. There is no trace of a paraconid or metaconid. The anterior surface of $M_2$ is divided into a buccal carina and a lingual carina, which together enclose an anterior groove embracing the talonid of $M_1$. Both carinae have a cuspule at the level of the $M_1$ talonid. The talonid on $M_2$ is like that on $P_4$ and $M_1$, a single relatively blunt wedge-shaped cusp. The crown of $M_2$ is surrounded by a narrow basal cingulid. Measurements for lower teeth are included in Table 1.

**Cranium**. The braincase of CGM 60584 was reconstructed by molding and casting the pieces of cranium that were recovered. The casts were then assembled into a single unit (Fig 7). The weight of the original pieces and poorly fitting contact surfaces between the original pieces precluded using them for this assembly. Several features of the assembled braincase are distinctive. First, the braincase of *Aegicetus* was relatively narrow. If we take the occipital condyles (*oc*) as reference points and the bicondylar width across the condyles (10.8 cm in CGM 60584) as a measure of scale, protocetids usually have exoccipitals (*Eoc*) that project laterally from the midline substantially more than the bicondylar width. Here the exoccipital projection from the midline is approximately the same (10.7 cm) as the bicondylar width. Corresponding measurements for *Georgiacetus vogtlensis* are 10.4 cm and 12.0 cm, where the latter distance is substantially greater than the former. Narrowness of the braincase is not due to bilateral compression because the spacing of occipital condyles in the reconstruction matches the spacing of condylar facets on the atlas vertebra.

In most protocetids, indeed most archaeocetes, the exoccipitals project laterally from the foramen magnum (*fm*) at the level of the occipital condyles. However, in *Aegicetus* the exoccipitals are deflected ventrally at an angle of about 45˚ (Fig 7A), and both consequently lie lateral and ventral to the occipital condyles. The jugal process of the squamosal (*jp*) is similarly less flaring than is typical for protocetids. These traits too indicate a relatively narrow braincase. One additional feature worthy of note that is seen in posterior view is the prominent nuchal flange (*nf*) of bone rising above and to the left of the foramen magnum. The nuchal flange on the right side of the occiput is broken but its base is preserved rising above and to the right of the foramen magnum. Protocetids commonly have paired nuchal tubercles in this position, but the well developed nuchal flanges seen here are distinctive.

The conformation of bones of the braincase seen in lateral and dorsal view (Fig 7B and 7C) is typical of protocetids, with the only features worthy of note being: (a) retention of a thin

lamina of bone on the left side representing the posterior process of the periotic, posterior to the external auditory meatus and extending close to the lateral margin of the meatus; and (b) the presence of relatively large anterior and posterior parietal emissary foramina (*pef*) in the parietal-squamosal suture on both sides of the braincase.

A piece of the supraorbital process of the frontal is shown in Fig 7B and 7C that has the form typical of supraorbital processes in protocetids. Due to breakage it no longer contacts the rest of the frontal where this crosses the dorsal midline of the skull (Fig 7C). The supraorbital process has a relatively flat dorsal surface that curves laterally above the orbit. The latter is bordered by a ventrally-projecting postorbital process (*pop*), which is again typical of protocetids.

**Dentary**. Much of the right dentary is preserved, in pieces, in CGM 60584 (Fig 6H and 6J). This is similar to the dentaries of other protocetids and basilosaurids in having a greatly enlarged mandibular canal (*mca*) running much of the length of the bone. The full opening of the mandibular foramen on the posteromedial surface of the dentary is not preserved, but the canal just anterior to this is minimally 7.5 cm deep. The canal shallows anteriorly. The mandibular canal housed blood vessels and nerves supplying soft tissues through mental foramina, but much of the canal was undoubtedly filled with a fat pad involved in sound conduction to the tympanic bulla and middle ear, as it is in modern cetaceans [38]. The ventral surface of the mandibular canal can be traced forward from the mandibular foramen to the most anterior portion of the dentary that is preserved (Fig 6J). One consequence of such a large mandibular foramen and extended canal is to weaken the dentary against compression, and the only parts of the dentary that remain recognizable are those ventral and lateral to the foramen and canal.

The mandibular symphysis (*ms*) was unfused and the rugose surface of the right dentary for articulation with its left counterpart extends posteriorly to the position of $P_2$. The middle portion of the dentary includes impressions of alveoli for $P_3$ through $M_3$ above the mandibular canal, and the posterior portion of the dentary includes the ascending ramus (*ar*) rising above the opening of the mandibular foramen. The mandibular condyle (*mc*) is well preserved on a neck of bone extending posterior to the ascending ramus. The condyle has an anterior articular surface facing dorsally, which wraps smoothly around the condyle to face posteriorly. The lateral surface of the condylar neck is convex and the medial surface is concave. Ventrally this was supported by the angular or posteroventral portion of the dentary (not recovered).

**Tympanic bulla**. Left and right tympanic bullae are preserved in CGM 60584. Both are compressed and damaged to some extent. These are typical in form to bullae of protocetid and basilosaurid archaeocetes [39, 40]. The right tympanic is better preserved than the left, and hence description is focused on the right bulla (Fig 8).

The lateral surface of the tympanic (Fig 8A) is dominated by the lateral eminence (*le*) which covers virtually the entire posterolateral surface posterior to the lateral furrow (*lf*) and base of the sigmoid process (*sp*). The ventral surface (Fig 8B) is divided by an oblique median furrow (*mf*) separating the anterior prominence (*ap*) and lateral or outer posterior prominence (*opp*) from the medial or inner posterior prominence (*ipp*). The latter forms a median eminence (*me*) opposite the lateral eminence (*le*).

The posterior surface of the tympanic (Fig 8C) has a centrally placed contact surface for the exoccipial (*ex*). Both posterior pedicles anchoring the posterior process are broken leaving only a small part of the elliptical foramen (*ef*). The dorsal surface of the tympanic (Fig 8D) is dominated by the massive involucrum (*in*) with a basisphenoid contact surface (*bc*) on the medial side. A portion of the lateral eminence (*le*), the sigmoid process (*sp*), and the anterodorsal crest (*ac*) on the lateral side of the tympanic are broken and compressed into the tympanic cavity (*tc*).

The right tympanic in Fig 8 is 51.8 mm in maximum length, from anterior prominence to outer posterior prominence, and 43.4 mm in maximum width from lateral eminence to medial

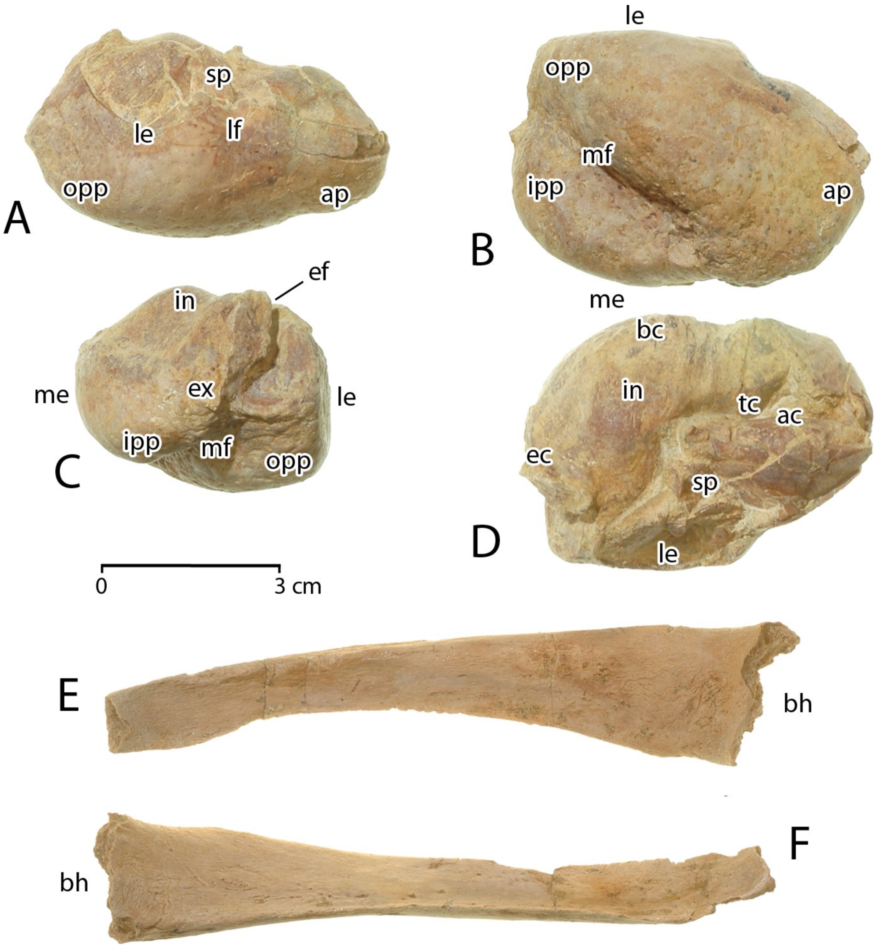


**Fig 8. Tympanic bulla and thyrohyals of *Aegicetus gehennae* type specimen (CGM 60584).** Right tympanic is shown in A, right lateral view; B, ventral view; C, posterior view; and D, dorsal view. The dorsal portion of the lateral eminence and much of the anterodorsal crest are compressed into the tympanic cavity. E, left thyrohyal in dorsal view. F, right thyrohyal in medial view. Abbreviations: *ac*, anterodorsal crest; *ap*, anterior prominence; *bc*, basisphenoid contact; *bh*, basihyal contact; *ef*, elliptical foramen; *ex*, exoccipital contact; *in*, involucrum; *ipp*, inner posterior prominence; *le*, lateral eminence; *lf*, lateral furrow; *me*, median eminence; *mf*, median furrow; *opp*, outer posterior prominence; *sp*, sigmoid process; *tc*, tympanic cavity.

https://doi.org/10.1371/journal.pone.0225391.g008

eminence. The left tympanic of CGM 60584 is 36.5 mm in maximum depth from the apex of the involucrum to the lowest point on the outer posterior prominence, measured parallel to the surface of the lateral eminence.

**Thyrohyals**. The ossified hyoid apparatus of cetaceans includes one midline element (basihyal) and four lateral elements (left and right stylohyals, and left and right thyrohyals). Thyrohyals articulate with the basihyal directly, whereas stylohyals are separated by intervening cartilaginous ceratohyals and epihyals [8, 41]. CGM 60584 includes left and right thyrohyals (Fig 8E and 8F), but the basihyal and stylohyals were not recovered.

Each thyrohyal has an enlarged, roughened proximal base where it connected via cartilage to the basihyal. The body narrows distally, with a rounded lateral surface and a sharply-keeled medial surface. It ends in a small roughened surface indicating that the distal end was cartilaginous. Viewed dorsally, the lateral surface is slightly convex and the medial keel is slightly concave. Viewed medially or laterally the thyrohyal is nearly straight, turning up slightly near the

distal end. The proximal ends of the left and right thyrohyals measure 25.0 × 17.4 mm and 25.7 × 17.6 mm in width and height, respectively. Both are.about 115 mm in length.

**Cervical vertebrae**. CGM 60584 includes seven cervical vertebrae (Fig 9A; links to three-dimensional images of each vertebra are provided here in Supporting Information [S1 Table]). Most are well preserved and all have vertebral epiphyses solidly fused to the centrum. Cervical measurements are listed in Table 2.

Cervical C1 (atlas) is a large, well-preserved, ring-shaped bone lacking any substantial centrum. C1 measures about 88 mm in greatest length anteroposteriorly, 175 mm in greatest width transversely across the alae or transverse processes, and 85 mm in greatest height dorsoventrally. Paired concave anterior surfaces for articulation with occipital condyles of the cranium measure 115 mm transversely across both surfaces, and each articular surface is about 53 mm high dorsoventrally. Paired flat posterior surfaces for articulation with C2 measure 106 mm transversely across both surfaces, and each of these articular surfaces is about 39 mm high dorsoventrally. They border a depression in the centrum remnant for articulation with the dens of C2.

Each of the C1 alae is divided into a larger, higher, anteriorly-concave dorsal portion and a smaller, lower, laterally-projecting transverse process with a narrow notch separating the two. There is no transverse foramen or vertebral canal perforating the left or right ala, and the vertebral artery on each side must have passed below the corresponding transverse process. It appears that the vertebral artery then passed dorsally and medially to reach an 8–10 mm diameter foramen in the medial wall of the ala, passing into a space that is open dorsally before continuing medially through a 9–11 mm diameter intervertebral foramen to reach the neural canal and enter the braincase as a cerebrospinal artery.

Cervical C2 (axis) has the form typical of protocetids, with a prominent dens extending the centrum anteriorly. This is bordered laterally by relatively flat surfaces for articulation with C1. The dorsal surface of the dens has a prominent ridge of bone extending posteriorly the length of the centrum. The ventral surface of the dens is smoothly convex. Posterior to the dens the centrum bears a triangular, ventrally-projecting hypapophysis. There is a prominent, curving, posterolaterally-directed transverse process lateral to the centrum. This forms the ventral and lateral border of the 9–11 mm diameter vertebrarterial canal. Tracing the vertebral artery anteriorly, it seemingly flared laterally and ventrally to pass behind the C1 articular surfaces on C2 and then below the transverse processes of C1.

The neural arch of C2 rises from the left and right posterolateral portions of the dorsal surface of the centrum. Postzygapophyses project from the dorsal laminae of the neural arch, with flat surfaces that face posteriorly, ventrally, and laterally. The neural spine has a total length of about 94 mm. This has an anterior ridge of bone that projects some 15 mm anteriorly from the dorsal laminae and then flattens as it rises dorsally and posteriorly.

Cervical C3 has a robust centrum with a distinct but relatively flat hypapophysis. Transverse processes, if present, are broken. Left and right vertebrarterial canals are some 11 × 15 mm in diameter. The neural arch rises from the posterior portions of the dorsolateral margins of the centrum. Pre- and postzygapophyses arise from the neural laminae. Prezygapophyses are slightly concave and face anteriorly, dorsally, and medially. Postzygapophyses are slightly convex and face ventrally, laterally, and only slightly posteriorly. The neural arch is nearly flat, with no neural spine.

Cervical C4 is very similar to C3, with a robust centrum and a distinct but relatively flat hypapophysis. Transverse processes were present but broken. Left and right vertebrarterial canals were large, but breakage precludes measurement. The neural arch rises from virtually the entire dorsolateral margin on the left and right sides of the centrum. Pre- and postzygapophyses arise from the neural laminae. Prezygapophyses are slightly concave and face dorsally,

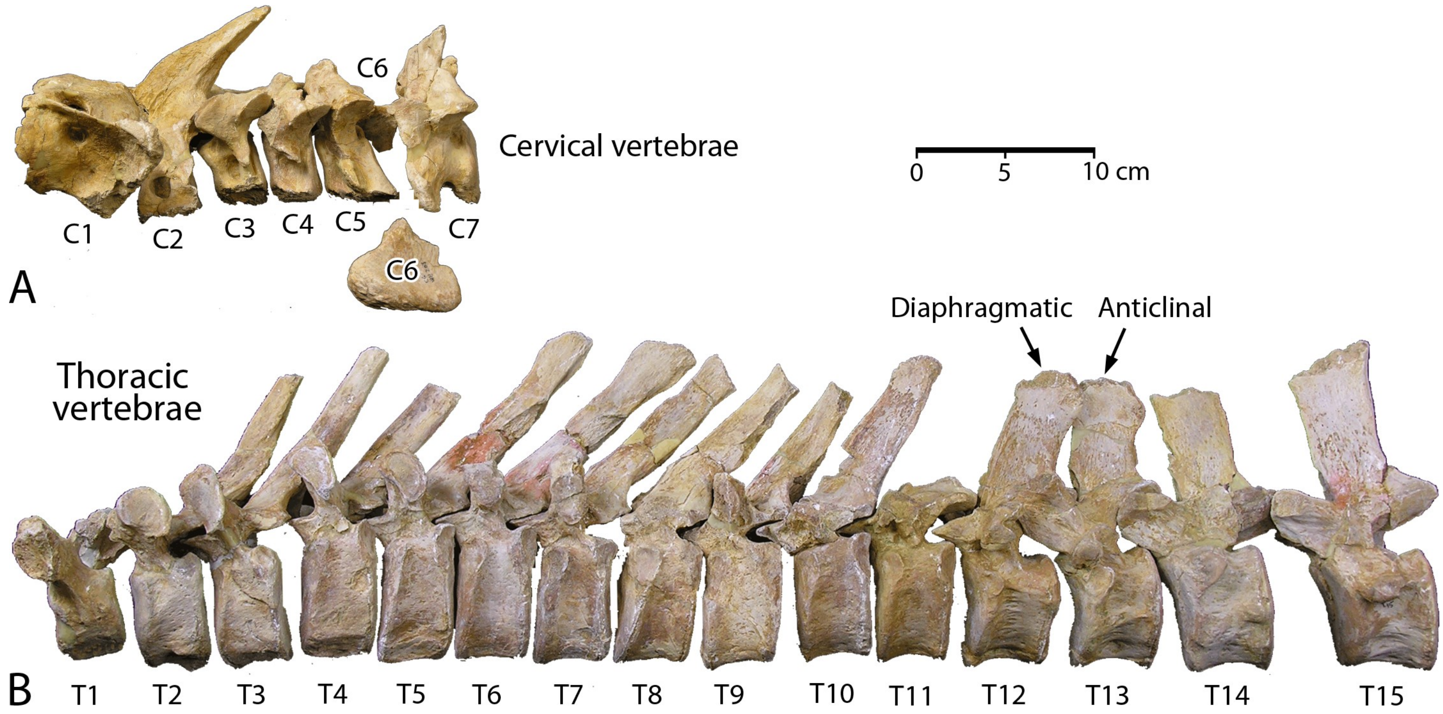


**Fig 9. Cervical and thoracic vertebrae of *Aegicetus gehennae* type specimen (CGM 60584).** A, cervical vertebrae C1 through C7 in left lateral view. B, thoracic vertebrae T1 through T15 in left lateral view. Note that most of the centrum of C6 is missing. T12 is diaphragmatic with dorsally-facing prezygapophyses and laterally-facing postzygapophyses. T13 is anticlinal, separating preceding thoracics with neural spines oriented dorsally and posteriorly from succeeding thoracics with neural spines oriented dorsally and anteriorly.

https://doi.org/10.1371/journal.pone.0225391.g009

medially, and slightly anteriorly. Postzygapophyses are slightly convex and face ventrally, laterally, and slightly posteriorly. The neural arch is relatively flat, with a short but distinct neural spine rising some 12 mm above the neural canal.

There is some uncertainty about identification of cervicals C5 and C6. The more complete of the two is identified as C5 because it articulates well with C4 and it does not articulate well with C7. The centrum of C5 has a roughened ventral surface but no real hypapophysis. Transverse processes were prominent but these are now broken. Vertebrarterial canals measure about 15 × 16 mm. The neural arch rises from the entire dorsolateral margin of the centrum. Pre- and postzygapophyses are similar to those on preceding vertebrae. The neural arch is distinctly arched and has a short but distinct neural spine rising some 17 mm above the neural canal.

The centrum of cervical C6 was not recovered, but C6 is represented by the distal portion of a massive ventrally-positioned transverse process, seemingly from the right side. This has a maximum anteroposterior diameter of 62 mm. C6 is also represented by the right portion of the neural arch with pre- and postzygapophyses.

Cervical C7 is nearly complete. The centrum has a characteristically flattened appearance, flattened anteroposteriorly, which is due to a relatively narrow anterior articular surface that is only 54.5 mm wide and a relatively broad posterior articular surface that is 70.0 mm wide. The posterior surface of the centrum has faint depressions at its ventrolateral margins for articulation with capitula of the first ribs. The centrum has no hypapophysis. Transverse processes are prominent but these are not perforated by vertebrarterial foramina. Pre- and postzygapophyses resemble those of preceding vertebrae. The neural arch rises above the level of the

Table 2. Measurements of vertebrae of CGM 60584, type specimen of *Aegicetus gehennae*.

| Vertebra | Centrum length | Centrum width | Centrum height | Neural canal width | Neural canal height | Vertebra | Centrum length | Centrum width | Centrum height | Neural canal width | Neural canal height |
|---|---|---|---|---|---|---|---|---|---|---|---|
| C1 | 29.3 | | 21.4 | 52.3 | 38.3 | T14 | 46.6 | — | — | — | — |
| C2 | 46.4 | 51.4 | 42.8 | 26.4 | 18.9 | T15 | 50.1 | 69.8 | 62.0 | 23.2 | 17.0 |
| C3 | 30.4 | 51.9 | 42.9 | 35.1 | — | L1 | 58.1 | 71.6 | 65.8 | 23.5 | 17.4 |
| C4 | 29.2 | 51.6 | 41.7 | 36.6 | 19.1 | L2 | 59.3 | 77.4 | 63.3 | 27.8 | 17.1 |
| C5 | 31.8 | 60.0 | 44.4 | 39.2 | 22.5 | L3 | 65.6 | 82.3 | 62.7 | 32.0 | 17.1 |
| C6 | — | — | — | — | — | L4 | 60.4 | 82.1 | 63.8 | 41.7 | 16.6 |
| C7 | 33.2 | 70.0 | 46.5 | 40.9 | 25.0 | S1 | 66.6 | 83.0 | 57.1 | 40.8 | 13.8 |
| T1 | 37.9 | 69.8 | 43.5 | 38.6 | — | S2 | 67.2 | 83.9 | 58.0 | 35.5 | 13.1 |
| T2 | 39.9 | 65.5 | 48.7 | 30.0 | — | S3 | 72.6 | 82.6 | 61.8 | 29.5 | 12.3 |
| T3 | 40.0 | 58.7 | 50.8 | — | 25.0 | S4 | 70.7 | 86.8 | 61.5 | 26.8 | 14.8 |
| T4 | 41.3 | — | — | — | — | Ca1 | 77.1 | 83.4 | 69.0 | 22.3 | 14.5 |
| T5 | 41.1 | — | — | — | — | Ca2 | 75.0 | 84.9 | 67.2 | 23.3 | 15.7 |
| T6 | 39.9 | — | — | — | — | Ca3 | 79.6 | 81.3 | 73.1 | 20.4 | 15.7 |
| T7 | 40.6 | — | — | — | — | Ca4 | 80.0 | 82.8 | 72.8 | 18,0 | 16,3 |
| T8 | 38.9 | — | — | — | — | Ca5 | 81.7 | 76.3 | 74.4 | 16.6 | 14.1 |
| T9 | 38.7 | — | — | — | 22.4 | Ca6 | 84.0 | 80.0 | 65.3 | — | — |
| T10 | 39.6 | 67.4 | 54.6 | — | — | Ca7 | 85.6 | 68.9 | 70.4 | 14.1 | 12.0 |
| T11 | 40.4 | 70.1 | 58.8 | 23.9 | 16.0 | Ca8 | 83.9 | 70.5 | 69.7 | — | — |
| T12 | 44.7 | 71.3 | 56.8 | 25.7 | 17.3 | Ca9 | 81.5 | — | 73.2 | — | — |
| T13 | 47.3 | — | — | — | — | — | — | — | — | — | — |

Measurements, in mm, are given for cervical (C), thoracic (T), lumbar (L), sacral (S), and caudal (Ca) vertebrae.

https://doi.org/10.1371/journal.pone.0225391.t002

zygapophyses and the arch is capped by a neural spine rising minimally 33.4 mm above the neural canal.

**Thoracic vertebrae**. CGM 60584 includes 15 thoracic vertebrae (Fig 9B; links to three-dimensional images of each vertebra are provided here in Supporting Information [S1 Table]). Thoracics are identified as such by the presence of capitular and tubercular facets for articulation of associated ribs. Most are well preserved, and all have vertebral epiphyses solidly fused to the centrum. Augmentation of the number of thoracics from 13 in older and more primitive protocetids [1, 4–5] to 15 in *Aegicetus gehennae* reflects the serial addition of ribs to what were formerly anterior lumbar vertebrae. Thoracic measurements are listed in Table 2.

Thoracic T1 is distinctive in having a broad and relatively low vertebral centrum, making the anterior and posterior surfaces broadly elliptical. The centrum has capitular facets for rib heads at the lateral margins of both its anterior and posterior surfaces. Pedicles and laminae for the neural arch are robust, relatively short anteroposteriorly, and elongated transversely. These rise from the anterior margin of the centrum. Pre- and postzygapophyses extend anteriorly and posteriorly from the lower part of each lamina. Prezygapophyses face dorsally, medially, and slightly anteriorly. Postzygapophyses face ventrally and slightly laterally and posteriorly. Laminae extend laterally below each prezygapophysis to support a tubercular facet for rib R1. Tubercular facets face laterally, ventrally, and slightly anteriorly, and each is surmounted by a small but distinct metapophysis. The neural arch of T1 is broken so nothing can be said about it. T1 measures 150 mm in maximum breadth across the tubercular facets.

Thoracic T2 has a vertebral centrum similar in height to that of T1, but the centrum is narrower transversely. The anterior surface is narrowly elliptical, but the posterior surface is more

triangular in outline. There are again capitular facets for rib heads at the lateral margins of the anterior and posterior surfaces of the centrum. Pedicles and laminae for the neural arch are robust, relatively short anteroposteriorly, and elongated transversely. These rise from the anterior margin of the centrum. Pre- and postzygapophyses extend anteriorly and posteriorly from the upper part of each lamina. Prezygapophyses face dorsally and slightly medially and anteriorly. Postzygapophyses face ventrally and slightly medially and posteriorly. Laminae extend laterally from the zygapophyses as transverse processes, and each ends in a laterally and slightly ventrally and anteriorly facing tubercular facet for rib R2. The tubercular facets are positioned at about the level of the zygapophyses, and metapophyses are again small. The neural arch is complete, and the neural spine is virtually complete. The base of the neural spine is broad, linking left and right postzygapophyses. The neural spine narrows dorsally to become robust and triangular in cross section. This robust triangular portion extends about two-thirds of the length of the spine, beyond which the spine is narrower in cross section. T2 measures 126 mm in maximum breadth across the tubercular facets. The neural spine was about 130 mm in length measured from the anterior margin of the neural arch above the neural canal.

Thoracic T3 has a vertebral centrum that is only slightly wider than its height. Anterior and posterior surfaces have the shape of rounded triangles. Capitular facets for rib heads are positioned at the dorsolateral margins of the anterior and posterior surfaces of the centrum. Pedicles and laminae for the neural arch rise from the anterior margin of the centrum and extend posteriorly and laterally toward the posterior capitular facets. Pre- and postzygapophyses extend anteriorly and posteriorly from the upper part of each lamina. Prezygapophyses face dorsally and slightly laterally and anteriorly. Postzygapophyses face ventrally and slightly medially and posteriorly. Transverse processes rise dorsally and laterally from the laminae. Each ends in a laterally and slightly ventrally and anteriorly facing tubercular facet for rib R3. The tubercular facets are positioned above the level of the zygapophyses, and the metapophyses now rise some 10–12 mm above each tubercular facet. The neural arch is complete, and the neural spine is virtually complete. Here again the base of the neural spine is broad, linking the postzygapophyses, The neural spine is triangular in cross section near the neural arch and it then becomes narrower in cross section. T3 is about 110 mm in maximum breadth across the tubercular facets. The neural spine is about 127 mm in length as preserved, measured from the anterior margin of the neural arch above the neural canal, and it was probably closer to 140 mm in length when complete.

Thoracic T4 appears to have been very similar to T3 in size and form, but detailed comparison is difficult because the centrum is compressed laterally, distorting its shape. The tubercular facets for R4 are again positioned above the level of the zygapophyses, and the metapophyses again rise some 10–12 mm above each tubercular facet. The neural arch is complete and the neural spine is partially preserved. The base of the neural spine is again broad, linking the postzygapophyses, It is triangular in cross section through much of its preserved length. T4 is about 95 mm in maximum breadth across the tubercular facets (which do not seem to have been affected by compression). The neural spine is about 120 mm in length as preserved, measured from the anterior margin of the neural arch above the neural canal, and it was probably closer to 150 mm in length when complete.

Thoracic T5 appears to have been very similar to T3 and T4 in size and form, but detailed comparison is again difficult because the centrum is compressed laterally, distorting its shape. The tubercular facets for R5 are again positioned above the level of the zygapophyses, and the metapophyses again rise some 10 mm above each tubercular facet. The neural arch is complete. The base of the neural spine is again broad, linking the postzygapophyses, It is triangular in cross section through much of its preserved length, narrowing dorsally. As preserved, T5 measures about 86 mm in maximum breadth across the tubercular facets. The neural spine of T5 is

complete. It measures 165 mm in length, measured from the anterior margin of the neural arch above the neural canal.

Thoracic T6 is virtually complete but the centrum is again compressed laterally, distorting its shape. It appears to have been similar to T3, T4, and T5 in size and form. The tubercular facets for R6 are again positioned above the level of the zygapophyses, and the metapophyses again rise some 10 mm above each tubercular facet. The neural arch is complete. The base of the neural spine is again broad, linking the postzygapophyses, It is triangular in cross section through much of its preserved length, narrowing dorsally. As preserved, T6 is about 85 mm in maximum breadth across the tubercular facets. The neural spine of T6 is complete. It measures 167 mm in total length, measured from the anterior margin of the neural arch above the neural canal. The distal (dorsal) end of the neural spine is expanded anteroposteriorly. The distal end measures 30.7 mm in anteroposterior length and 8.5 mm in transverse width.

Thoracic T7 is virtually complete but the centrum is compressed laterally, distorting its shape. It appears to have been similar to T3–T6 in size and form. Prezygapophyses are a little closer together than those on preceding thoracics. The tubercular facets for R7 are a little lower, positioned at the level of the zygapophyses. Metapophyses rise some 12–15 mm above each tubercular facet. The neural arch is complete. The base of the neural spine is broad, linking the postzygapophyses. It is triangular in cross section through much of its preserved length, narrowing dorsally. As preserved, T7 is about 87 mm in maximum breadth across the tubercular facets. The neural spine of T7 is missing the distal portion. It measures 145 mm in length, as preserved, and the total length was probably about the same as that for T6.

Thoracic T8 is virtually complete but the centrum is compressed laterally, distorting its shape. It appears to have been similar to T3–T7 in size and form. The tubercular facets for R8 are at the level of the zygapophyses. The metapophysis preserved on the right side rises some 12 mm above the corresponding tubercular facet. The neural arch is complete. The base of the neural spine is broad, linking the postzygapophyses. It is triangular in cross section through much of its preserved length, narrowing dorsally. The maximum breadth of T8 cannot be estimated because the left transverse process and its tubercular facet are broken. The neural spine of T8 is missing its distal portion. The spine measures 136 mm in length, as preserved, but the total length was greater when the spine was complete.

Thoracic T9 is virtually complete but the centrum is again compressed laterally, distorting its shape. It appears to have been similar to T3–T8 in size and form. The tubercular facets for R9 are at the level of the zygapophyses. Metapophyses rise some 12 mm above the tubercular facets. The neural arch is complete. The base of the neural spine is broad, linking the postzygapophyses. It is triangular in cross section through much of its preserved length, narrowing dorsally. As preserved, T9 measures about 89 mm in maximum breadth across the tubercular facets. The neural spine of T9 is missing the distal tip. It measures 114 mm in length, as preserved, which appears to be close to the total length.

Thoracic T10 is virtually complete and the centrum appears little distorted. The vertebra appears to have been similar to T3–T9 in size and form. Tubercular facets for R10 are at the level of the zygapophyses. The metapophyses rise some 12–13 mm above the tubercular facets. The neural arch is complete. The base of the neural spine is broad, linking the postzygapophyses. The neural spine is triangular in cross section through much of its preserved length, narrowing dorsally. As preserved, T10 measures about 92 mm in maximum breadth across the tubercular facets. The neural spine of T10 is complete, and it measures 123 mm in dorsoventral length.

Thoracic T11 has a complete centrum and neural arch, but the neural spine is missing. The centrum appears little distorted. Prezygapophyses face almost directly upward and postzygapophyses face almost directly downward. Tubercular facets for R10 are at the level of the

zygapophyses. The metapophyses rise some 18 mm above the tubercular facets, and these show incipient bifurcation into anterior and posterior moieties. The neural arch is complete and resembles those of preceding vertebrae. As preserved, T11 measures about 99 mm in maximum breadth across the tubercular facets.

Thoracic T12 is virtually complete and the centrum is little distorted. T12 differs from preceding thoracics in having a centrum that is less high dorsoventrally and broader transversely. Prezygapophyses face dorsally and slightly anteriorly, but postzygapophyses differ from those of preceding thoracics in facing laterally as much as they do ventrally. These differences in orientation of the pre- and postzygapophyses indicate that T12 is the diaphragmatic vertebra. Tubercular facets for R12 are small, below the level of the zygapophyses, and extend toward their corresponding capitular facets. Metapophyses rise some 28 mm above the tubercular facets, and the metapophyses are clearly bifurcated anteriorly and posteriorly. The neural arch is complete. The base of the neural spine is broad, anchoring the postzygapophyses posteriorly. The neural spine is triangular in cross section and robust through its entire length. Small alae flare posterolaterally near the apex of the neural spine. As preserved, T12 measures about 94 mm in maximum breadth across the tubercular facets. The neural spine of T12 is complete, and it measures 102 mm in total length dorsoventrally. The distal end of the spine measures 37.1 mm in anteroposterior length and 21.0 mm in transverse width.

Thoracic T13 is virtually complete and the centrum is little distorted. The centrum of T13 resembles that of T12 in being relatively low dorsoventrally and broad transversely. Prezygapophyses face dorsally, medially, and slightly anteriorly, and postzygapophyses face laterally as much as they do ventrally. Tubercular facets for R13 are small, well below the level of the zygapophyses, and almost confluent with their corresponding capitular facets. Metapophyses are relatively short but now rise above the prezygapophyses instead of the tubercular facets. The neural arch is distorted but complete. The base of the neural spine is broad, anchoring the postzygapophyses posteriorly. The neural spine rises above the centrum almost vertically, indicating that T13 is the anticlinal vertebra. The neural spine itself is narrowly triangular and robust in cross section through its entire length. Here, as in T12, small alae flare posterolaterally near the apex of the neural spine. As preserved, T13 is about 89 mm in maximum breadth across the tubercular facets. The neural spine of T13 is complete, and it measures 85 mm in dorsoventral length. The distal end of the spine measures 38.9 mm in anteroposterior length and 13.9 mm in transverse width.

Thoracic T14 is virtually complete and the centrum is little distorted. T14 resembles T12 and T13 in having a centrum that is relatively low dorsoventrally and broad transversely. Prezygapophyses face dorsally, medially, and slightly anteriorly, and postzygapophyses face laterally as much as they do ventrally. Tubercular and capitular facets for R14 are confluent and project laterally a short distance from the anterolateral surface of the centrum. Metapophyses are robust and rise dorsally from the lateral surface of the prezygapophyses. The neural arch is distorted but complete. The base of the neural spine is broad, anchoring the postzygapophyses posteriorly. The neural spine is inclined anteriorly above the centrum, indicating that T14 is post-anticlinal. The neural spine itself is long anteroposteriorly and compressed laterally, but robust in cross section through its entire length. There are no alae flaring posterolaterally near the apex of the neural spine. As preserved, T14 measures about 86 mm in maximum breadth across the rib facets. The neural spine of T14 is complete, and it measures 85 mm in dorsoventral length. The distal end of the spine measures 36.8 mm in anteroposterior length and 11.7 mm in transverse width. This distal end has an obliquely truncated surface gouged in a way that suggests a shark or other predator or scavenger may have removed cartilage or cartilaginous bone.

Thoracic T15 is virtually complete and the centrum is little distorted. T15 resembles T12–T14 in having a centrum that is relatively low dorsoventrally and broad transversely. Prezygapophyses face more medially than dorsally, and postzygapophyses face more laterally than ventrally. Facets for articulation with rib R15 are on a transverse process extending some 24 mm laterally from the lateral surface of the centrum. The remaining features of T15 resemble those of T14 closely. As preserved, T15 measures about 104.9 mm in maximum breadth across the transverse processes and rib facets. The neural spine of T15 is complete, measuring 95 mm in total length dorsoventrally. The distal end of the spine is 46.2 mm in anteroposterior length and 13.7 mm in transverse width. Here again the distal end has an obliquely truncated surface gouged in a way that suggests a shark or other scavenger may have removed cartilage or cartilaginous bone.

**Lumbar vertebrae**. CGM 60584 includes four lumbar vertebrae, all well preserved (Fig 10A; links to three-dimensional images of each vertebra are provided here in Supporting Information [S1 Table]). All have vertebral epiphyses solidly fused to the centrum. Reduction in the number of lumbars—from six in older and more primitive protocetids [1, 4–5], to the four present in *Aegicetus gehennae*—appears to reflect the serial addition of ribs to what were formerly anterior lumbar vertebrae. Lumbar measurements are listed in Table 2.

Lumbar L1 has a cylindrical centrum, compressed laterally, with bases of relatively small transverse processes arising from the lateral margins of the centrum. The transverse processes themselves are broken and missing, so their full size is unknown. The neural arch rises from the anterior three-quarters of the dorsolateral surface of the centrum. Prezygapophyses extend anteriorly from the neural arch, positioned above and lateral to the neural canal, with surfaces facing medially and slightly dorsally. Metapophyses cap the prezygapophyses. Postzygapophyses extend posteriorly from the neural arch, positioned above and lateral to the neural canal, with surfaces facing laterally and slightly ventrally. Width across the transverse processes cannot be determined for L1. The neural spine is complete. It measures 110 mm in total length dorsoventrally. The dorsal end of the spine measures 49.8 mm in anteroposterior length and 19.0 mm in transverse width.

Lumbar L2 is similar to L1 but the centrum is not compressed laterally. Transverse processes are present, arising from the lateral margins of the centrum. Width across the transverse processes is about 160 mm. The neural spine is complete. It measures 110 mm in total length dorsoventrally. The dorsal end of the spine measures 43.6 mm in anteroposterior length and 16.5 mm in transverse width.

Lumbar L3 is similar to L1 and L2, but the centrum is broader and the transverse processes more robust. Width across the transverse processes is 185 mm. The neural spine is complete. It measures 95 mm in total length dorsoventrally. The dorsal end of the spine measures 28.6 mm in anteroposterior length and 11.5 mm in transverse width.

Lumbar L4 is similar to the preceding lumbars. The centrum is broad like that of L3, but the transverse processes are less robust. Width across the transverse processes is about 204 mm (estimated by doubling the length on the right side). The neural spine is complete. It measures 102 mm in total length dorsoventrally. The dorsal end of the spine measures 38.0 mm in anteroposterior length and 8.7 mm in transverse width.

**Sacral vertebrae**. CGM 60584 includes four sacral vertebrae (Fig 10B; links to three-dimensional images of each vertebra are provided here in Supporting Information [S1 Table]). All are well preserved and all have vertebral epiphyses solidly fused to the sacrum. The first sacral has expanded auricular processes at the ends of the transverse processes for articulation with left and right innominates. The following three vertebrae are called sacrals because of their homology with the sacral vertebrae of older and more primitive protocetids, some of which retained a fused four-centrum sacrum [1, 4–5]. Sacral measurements are listed in Table 2.

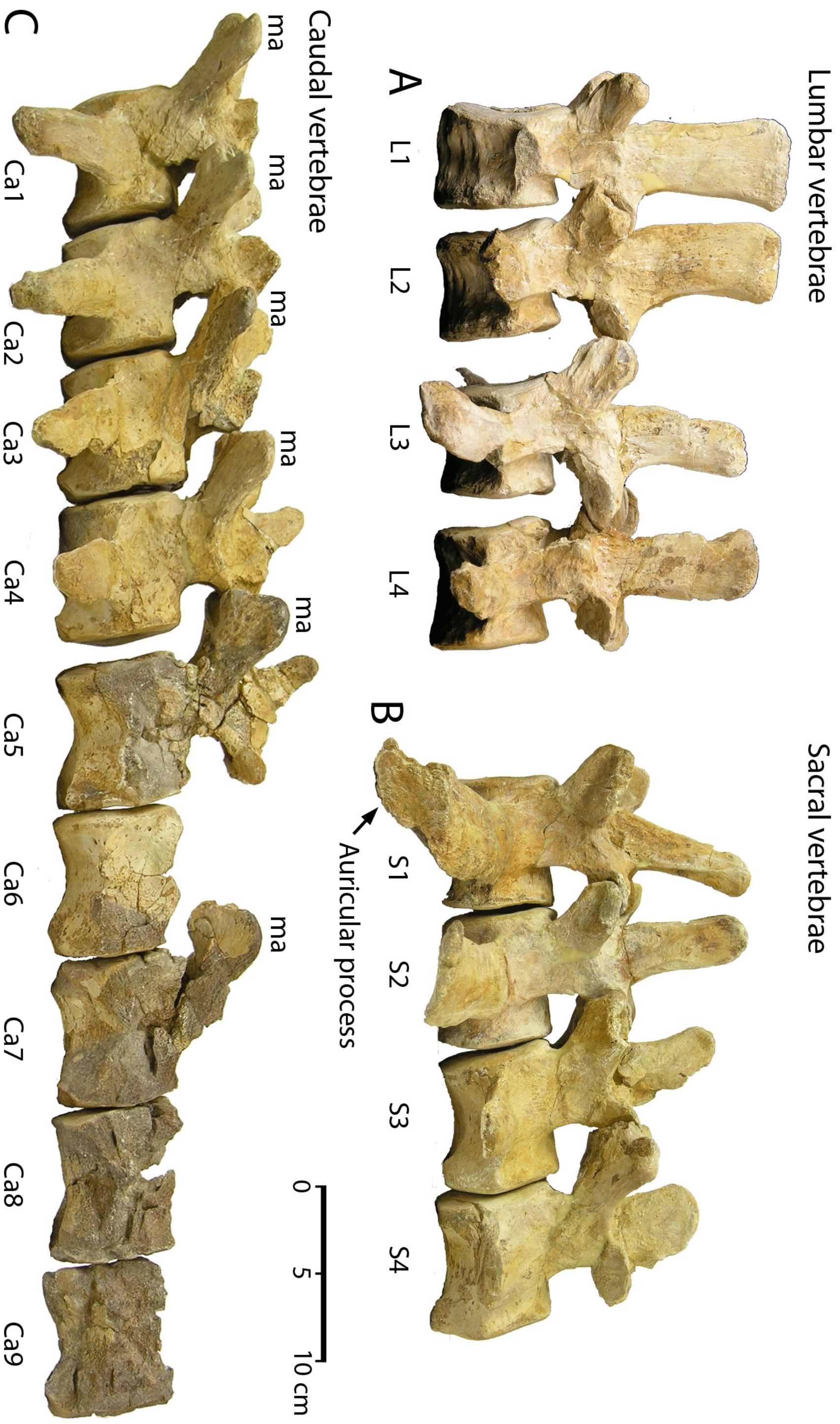


**Fig 10. Lumbar, sacral, and caudal vertebrae of *Aegicetus gehennae* type specimen (CGM 60584).** A, lumbar vertebrae L1 through L4 in left lateral view. B, sacral vertebrae S1 through S4 in left lateral view. C, caudal vertebrae Ca1 through Ca9 in left lateral view. Note the prominent auricular process on S1, the lack of fusion, and the absence of secondary articulations on all sacral vertebrae. All of the well preserved anterior caudal vertebrae have exceptionally large metapophyses (*ma*).

https://doi.org/10.1371/journal.pone.0225391.g010

Sacral S1 has a centrum in the form of a broadened cylinder. The anterior surface is elliptical in outline, but the posterior surface is more hexagonal with flattened dorsal and ventral margins. Massive left and right transverse processes project laterally, anteriorly, and slightly ventrally from lateral surfaces of the centrum. Each ends in an expanded auricular process with a flat, parasagittal articular surface. The neural arch rises from the anterior two-thirds of the dorsolateral surface of the centrum. Prezygapophyses extend anteriorly from the neural arch, positioned above and lateral to the neural canal, with slightly concave surfaces facing medially and dorsally. Metapophyses extend dorsally and laterally some 12 mm from the prezygapophyses. Postzygapophyses extend posteriorly from the neural arch, positioned above and lateral to the neural canal, with slightly convex surfaces facing laterally and ventrally. Width across the transverse processes is 240 mm. The neural spine is complete, relatively gracile, and slightly inclined posteriorly. It measures 95 mm in total length dorsoventrally, measured from the anterior margin of the neural arch above the neural canal. The dorsal portion of the spine measures 20.7 mm in anteroposterior length and 7.2 mm in transverse width.

Sacral S2 is similar to S1 but lacks the robust auricular process. It differs in having a slightly broader centrum. The posterior surface of the centrum is again hexagonal in outline, with flattened dorsal and ventral margins. There are swellings at the corners of the ventral margin where one might expect articulation with a chevron or haemal arch, but it is not clear that these are real articular surfaces. The transverse processes are curved like those on S1, but here they are flatter and more gracile. The right transverse process has an obliquely truncated surface gouged in a way that suggests a shark or other scavenger may have removed cartilage or cartilaginous bone. Width across the transverse processes is again 240 mm. The neural spine is complete, relatively gracile, and vertical in orientation. It measures 90 mm in total length dorsoventrally. The dorsal portion of the spine measures 26.2 mm in anteroposterior length and 7.3 mm in transverse width.

Sacral S3 is closely similar to S2. It differs in having slightly longer and more gracile transverse processes, and in having more prominent metapophyses. Swellings are again present on the posteroventral margin of the centrum where a chevron or haemal arch might be expected. Width of S3 across the transverse processes is estimated at about 250 mm (doubling the projection of the right transverse process). The neural spine is again complete, relatively gracile, and slightly inclined anteriorly. It measures 75 mm in total length dorsoventrally. The dorsal portion of the spine measures 22.2 mm in anteroposterior length and 6.1 mm in transverse width.

Sacral S4 is similar to S3. It has similarly gracile transverse processes, but these are a little longer. The metapophyses are more prominent, like those on succeeding caudal vertebrae. The metapophyses project some 20 mm dorsal and lateral to the prezygapophyses. Swellings are present on the posteroventral margin of the centrum where a chevron or haemal arch might be expected. Width across the transverse processes is estimated at about 270 mm (doubling the projection of the right transverse process). The neural spine is again complete, relatively gracile, and slightly inclined anteriorly. It rises 65 mm dorsoventrally above the neural canal. The dorsal portion of the spine measures 35.4 mm in anteroposterior length and 6.0 mm in transverse width.

**Caudal vertebrae**. CGM 60584 includes nine of a probable 20 or 21 caudal vertebrae (Fig 10C; links to three-dimensional images of each vertebra are provided here in Supporting Information [S1 Table]). Ca1 through Ca5 are well preserved, but Ca6–Ca9 lack part or all of their neural arch and zygapophyses. All nine of these caudals have vertebral epiphyses solidly fused to the sacrum. Measurements are listed in Table 2.

Caudal Ca1 has a centrum in the form of a broadened cylinder like those of preceding lumbar vertebrae. The anterior surface is elliptical in outline, but the posterior surface is more hexagonal with flattened dorsal and ventral margins. Robust left and right transverse processes

project laterally and slightly ventrally from lateral surfaces of the centrum. The left transverse process is complete, and it ends in a slightly expanded textured surface. The neural arch rises from the anterior two-thirds of the dorsolateral surface of the centrum. Prezygapophyses extend anteriorly from the neural arch, positioned above and lateral to the neural canal, with slightly concave surfaces facing medially and dorsally. Broad, relatively flat metapophyses extend dorsally, laterally, and anteriorly some 25 mm beyond the prezygapophyses. The size of the prezygapophyses and metapophyses taken together is enhanced by their co-ossification along the midline dorsal and anterior to the neural canal. The massive prezygapophyses and metapophyses extend well above and anterior to the centrum. Postzygapophyses are broken on this vertebra. There are distinct facets for articulation of a chevron or haemal arch present at the corners of the posteroventral margin of the centrum, and it is clear that this caudal had an associated chevron. The width across the transverse processes is 240 mm (doubling the projection of the right transverse process). The neural spine is broken near its base and thus its dimensions cannot be measured.

Caudal Ca2 is a little better preserved than Ca1, and it is very similar to Ca1 in form. The centrum is a broadened cylinder, with an elliptical anterior outline and a more hexagonal posterior outline. Robust left and right transverse processes project laterally as before, but also slightly posteriorly, and the distal ends are distinctly downturned. The neural arch, prezygapophyses, and metapophyses are similar to those of Ca1, and again the size of the metapophyses is enhanced by their co-ossification along the midline dorsal and anterior to the neural canal. Postzygapophyses are partially preserved, facing laterally and slightly ventrally. There are again distinct facets for articulation of a chevron present at the corners of the posteroventral margin of the centrum. The width across the transverse processes is 248 mm. The neural spine is broken above its base and its dimensions cannot be measured.

Caudal Ca3 is similar to Ca1 and Ca2. The centrum is cylindrical, with a circular anterior outline and a hexagonal posterior outline. Robust left and right transverse processes project laterally as before but also slightly posteriorly. The distal ends are again distinctly downturned. The neural arch, prezygapophyses, and metapophyses are similar to those of Ca1 and Ca2, with the massive prezygapophyses and metapophyses extending well above and anterior to the centrum. The neural canal is relatively small and circular in cross section. Postzygapophyses are broken on this vertebra. There are again distinct facets for articulation of a chevron present at the corners of the posteroventral margin of the centrum. The width across the transverse processes is 224 mm. The neural spine is broken near its base and its dimensions cannot be measured.

Caudal Ca4 is similar to the preceding caudals. The centrum is again cylindrical, with a circular anterior outline and a hexagonal posterior outline. Robust left and right transverse processes project laterally as before, but also slightly posteriorly. The distal ends are distinctly downturned. The neural arch, prezygapophyses, and metapophyses are similar to those of Ca1 through Ca3, with the massive prezygapophyses and metapophyses again extending well above and anterior to the centrum. The neural canal is relatively small and circular in cross section. A postzygapophysis is preserved on the right side, where it appears to be smaller than those on preceding caudals. There are again distinct facets for articulation of a chevron present at the corners of the posteroventral margin of the centrum. The width across the transverse processes is about 220 mm (doubling the projection of the left transverse process). The neural spine is small and delicate, measuring about 40 mm long at its base, with the spine narrowing rapidly to 20 mm. The spine rises about 40 mm above the dorsal surface of the neural arch, and it is only about 6 mm in transverse thickness.

Caudal Ca5 is similar to the preceding caudals, but differs in having perforated transverse processes. As before, the centrum is cylindrical, with a circular anterior outline and a

hexagonal posterior outline. Robust left and right transverse processes project laterally and slightly posteriorly. The distal ends are again distinctly downturned. The neural arch, prezygapophyses, and metapophyses are similar to those of Ca1 through Ca4, with massive prezygapophyses and metapophyses again extending well above and anterior to the centrum. The neural canal is relatively small and circular in cross section. Postzygapophyses are well preserved and face laterally and slightly downward. There are again distinct facets for articulation of a chevron present at the corners of the posteroventral margin of the centrum, but these are closer together than those on preceding vertebrae. The width across the transverse processes is only about 180 mm (doubling the projection of the right transverse process). The left and right transverse processes of Ca5 are perforated by a 7 mm diameter arterial foramen located centrally near the base of each transverse process. The neural spine is like that on Ca4 but here it rises about 50 mm above the dorsal surface of the neural arch.

Caudal Ca6 has a centrum similar to those of preceding caudals. It is cylindrical, with a circular anterior outline and a hexagonal posterior outline. Transverse processes were well developed but possibly not as robust as those on preceding caudals. Portions of the neural arch, prezygapophyses, and metapophyses that presumably belong to Ca6 are preserved as a separate element, weathered, that no longer articulates with the centrum. The neural canal was narrow, but little remains to indicate its cross-sectional shape. There are distinct facets for articulation of a chevron present at the corners of the posteroventral margin of the centrum, and these are again relatively close together. The transverse processes are broken so nothing can be said about their length. The medial margin of an arterial foramen remains, located centrally near the base of each transverse process.

Caudal Ca7 is similar to Ca5, but differs in having a centrum slightly smaller in diameter. As before, the centrum is cylindrical, with a circular anterior outline and a hexagonal posterior outline. Transverse processes are not preserved, but it appears that the medial border of an arterial foramen remains on the better-preserved right side of the centrum. The neural arch, prezygapophyses, and metapophyses are similar to those of Ca5, with the massive prezygapophyses and metapophyses again extending well above and anterior to the centrum. The neural canal is relatively small and circular in cross section. Postzygapophyses are not preserved. Chevron facets are present, close together, on the posteroventral margin of the centrum.

Caudal Ca8 is represented by a centrum only, which is similar to the centrum of Ca7. As before, this is cylindrical, with a circular anterior outline. Here it appears that the posterior outline was circular as well. Transverse processes are not preserved, but the medial border of an arterial foramen remains on the better-preserved right side of the centrum. Here as on Ca7 it is not clear whether the transverse process was simply perforated by passage of the artery, or whether the transverse process was divided in two by a transverse arterial cleft as occurs in some archaeocetes. The neural arch, prezygapophyses, and metapophyses are not preserved. Chevron facets are present, close together, on the posteroventral margin of the centrum.

Caudal Ca9 is represented by a partial centrum, very weathered, that resembles the centrum of Ca8. The medial border of an arterial foramen or arterial cleft remains on the better-preserved left side of the centrum. A single chevron facet is present close to the midline on the left side of the posteroventral margin of the centrum. There were undoubtedly two chevron facets, again positioned close together.

Portions of one or two additional vertebrae were found on the surface when CGM 60584 was first found, but these are too weathered to provide any useful information.

**Caudal chevron**. Anterior caudal vertebrae can be recognized by the presence of articular facets for chevron bones forming haemal arches enclosing and protecting the caudal artery. As described here, caudals Ca1 through Ca9 of CGM 60584 are known to have had chevrons. However, only one of these chevrons was recovered (Fig 11).

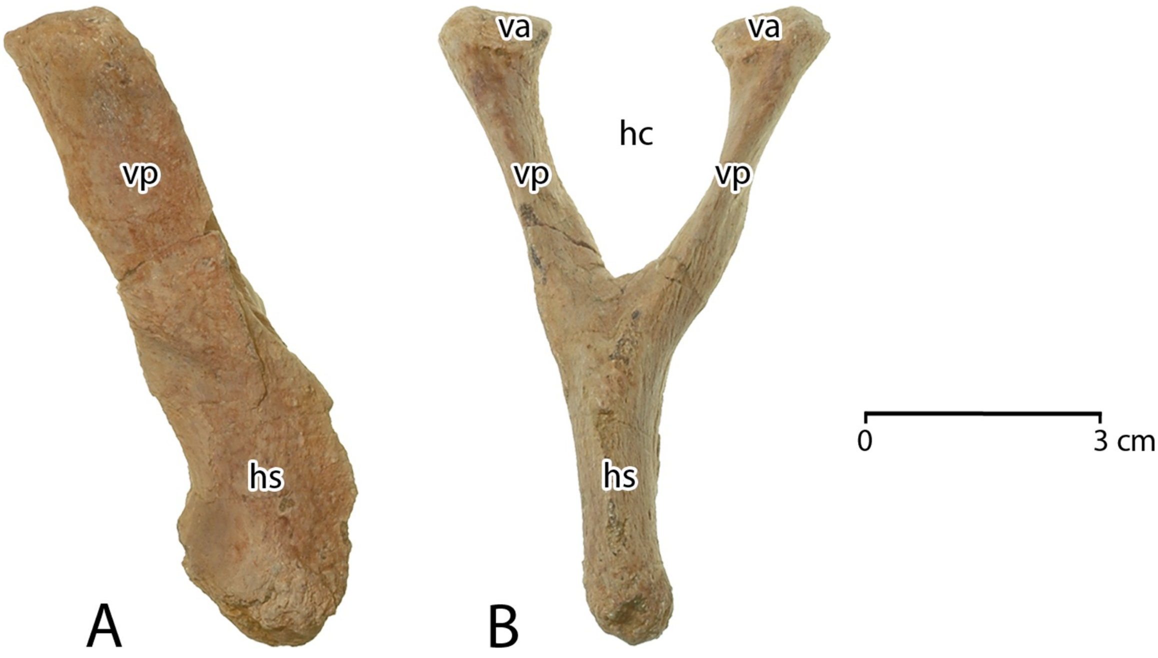


**Fig 11. Caudal chevron of *Aegicetus gehennae* type specimen (CGM 60584).** Specimen is shown in A, left lateral view; and B, posterior view. Abbreviations: *hc*, haemal canal; *hs*, haemal spine; *va*, vertebral articulation; *vp*, vertebral process.

https://doi.org/10.1371/journal.pone.0225391.g011

The chevron preserved as part of CGM 60584 is Y-shaped, with a pair of robust vertebral processes (*vp*) ascending anteriorly and vertically to form a haemal arch articulating with the corresponding vertebra. The haemal arch surrounds an open haemal canal (*hc*). An even more substantial haemal spine (*hs*) descends vertically from the haemal arch. The anterior surface of the haemal spine is partially concave, and the posterior surface is distinctly keeled. The vertebral articulations (*va*) on this chevron have articular surfaces centered 32 mm apart. This matches the distance between chevron facets on caudals Ca1, Ca2, and Ca3. More posterior caudals have more closely spaced chevron facets, so the chevron preserved here must be a first, second, or third chevron.

The chevron as a whole is 87.5 mm long dorsoventrally and 52.0 mm wide transversely. The haemal canal is 40 mm long dorsoventrally and 26.3 mm in maximum width transversely.

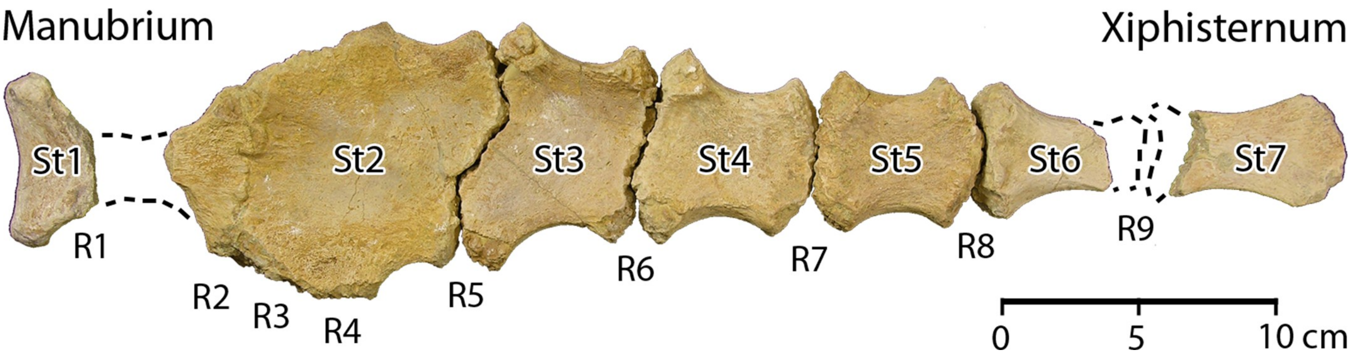


**Fig 12. Sternum of the *Aegicetus gehennae* type specimen (CGM 60584).** A, Virtually complete sternum showing sternebrae St1 (manubrium) through St7 (xiphisternum), all in dorsal view. Note the exceptionally broad ventral shield formed by the laterally expanded sternebrae, especially St2. Inferred insertions for left ribs 1 through 9 are labeled R1 through R9.

https://doi.org/10.1371/journal.pone.0225391.g012

The haemal spine is 47.5 mm long dorsoventrally, measured from the haemal canal to the ventral tip of the chevron, and the spine has a maximum length of 24.7 mm anteroposteriorly.

**Sternum**. There are seven sternal elements in *Aegicetus gehennae* (CGM 60584; Fig 12), which is the number in many artiodactyls [42]. In *Aegicetus* each element is separated from the next by a cartilaginous synchondrosis, with only the most anterior of these, between St1 and St2, being co-ossified. Measurements of individual elements are given in Table 3.

The body of St1 is missing but it was clearly narrower than the preserved proximal or distal ends. Distinct bosses resembling rib heads are present on anterolateral projections of St1. Their surfaces are smooth and not rugose like those of more distal insertions of costal cartilages.

The body of St2 is relatively flat and unusually broad, forming the shield that gives *Aegicetus* its name. The cranial surface of St2 is co-ossified with the caudal surface of St1, and there are rugose insertions on the left and right sides of this co-ossification for what are interpreted to be insertions of costal cartilages of the left and right second ribs (R2). Following these, each side of St2 has rugose swellings that appear to represent insertions for two additional ribs (interpreted as R3 and R4). There is then a concave indentation of smooth bone on each side of St2, which is rounded ventrally and slightly keeled dorsally. St2 is joined to St3 by an asymmetrical zig-zag synchondrosis that has rugose depressions on each side for insertion of the costal cartilage of a rib (interpreted as R5).

The bodies of St3, St4, and St5 are similar in length and similar in having depressions for costal cartilages at each corner. The ribs involved are interpreted as R5, R6, R7, and R8. The sternal elements themselves have bodies increasing progressively in thickness from front to back, and decreasing progressively in width. Each has a concave lateral margin that is rounded ventrally and slightly keeled dorsally.

St6 articulates with St5 in the normal way, but the body of St6 is longer and much thinner compared to its width. The caudal end is missing, but there were presumably rugose concavities on the lateral surfaces of the caudal end for insertion of one or more costal cartilages (interpreted as representing rib R9, and possibly R10 and R11). The most caudal portion of St6 preserved is narrower than the most cranial portion of St7 preserved, and these cannot be parts of one sternebra.

The cranial end of the xiphisternum, St7, is missing. The caudal end of the xiphisternum is relatively thin, and it flares laterally where it clearly expanded into a broader xiphoid cartilage. It is slightly concave dorsally and slightly convex ventrally, with a small but distinct swelling of bone on the ventral midline.

**Table 3. Measurements of the sternebrae in CGM 60584, type specimen of *Aegicetus gehennae*.**

| Position | Length | Proximal width | Distal width |
|---|---|---|---|
| **St1** | — | 63.3 | 61.8 |
| **St2** | 73.6 | 100.5 | 85.0 |
| **St3** | 64.8 | 89.5 | 70.0 |
| **St4** | 63.6 | 71.5 | 57.6 |
| **St5** | 61.2 | 58.8 | 54.8 |
| **St6** | — | 49.6 | — |
| **St7** | — | — | 41.8 |

Measurements, in mm, are given for each sternebra (St).

https://doi.org/10.1371/journal.pone.0225391.t003

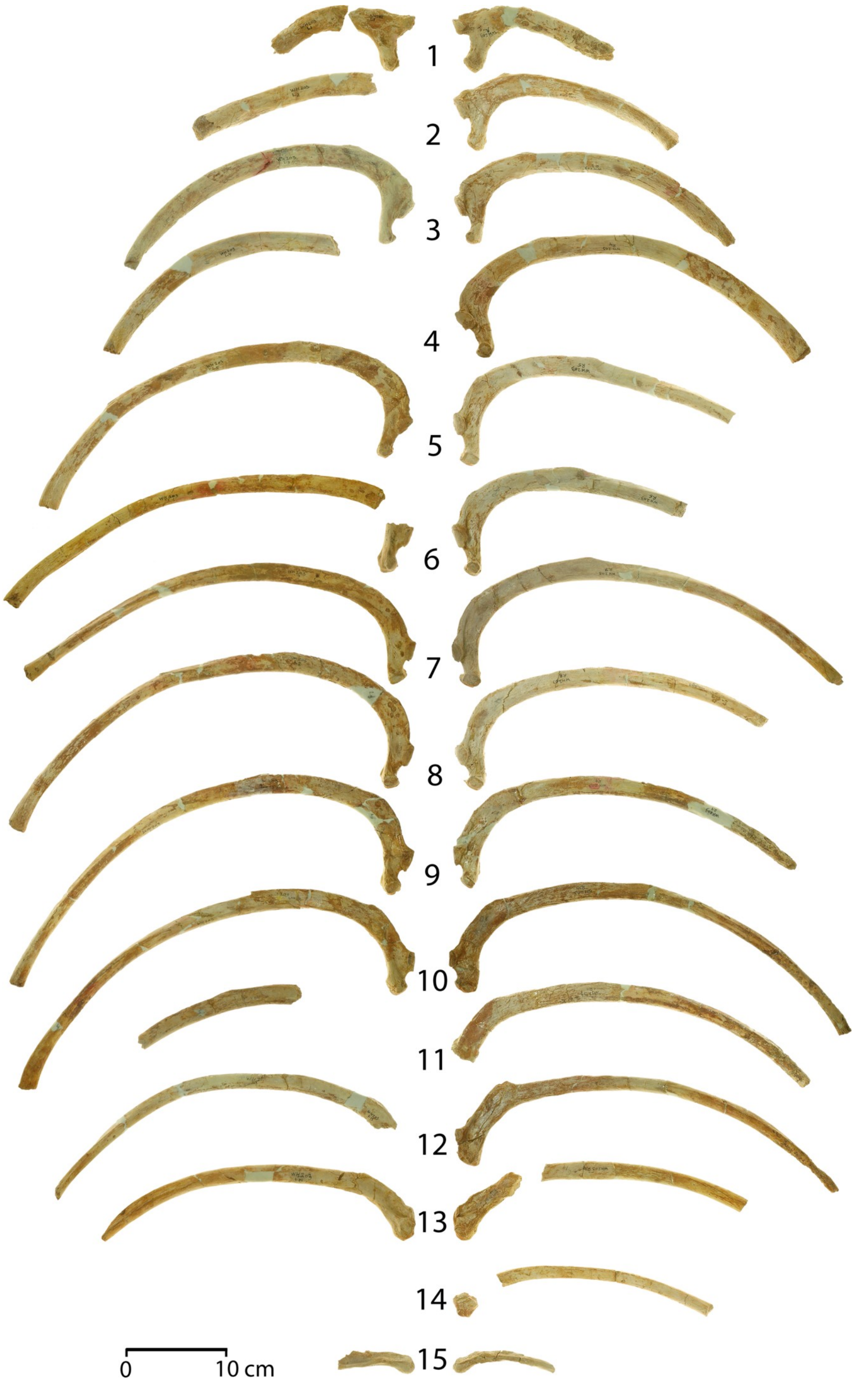


**Fig 13. ribs R1 through R15 in the *Aegicetus gehennae* type specimen (CGM 60584).** Ribs are shown in posterior view. All ribs are represented except left R14. Note that all of the ribs are slender and none is thickened or pachyostotic.

https://doi.org/10.1371/journal.pone.0225391.g013

**Ribs**. Twenty-nine ribs are present in CGM 60584 (Fig 13), 14 from the left side and 15 from the right side. The one rib not represented is left rib R14. Measurements are listed in Table 4.

All of the ribs of *Aegicetus gehennae* are relatively slender. The most robust are left and right ribs R1 and R2. The longest on each side is the rib R9, and the shortest is R15. Articular

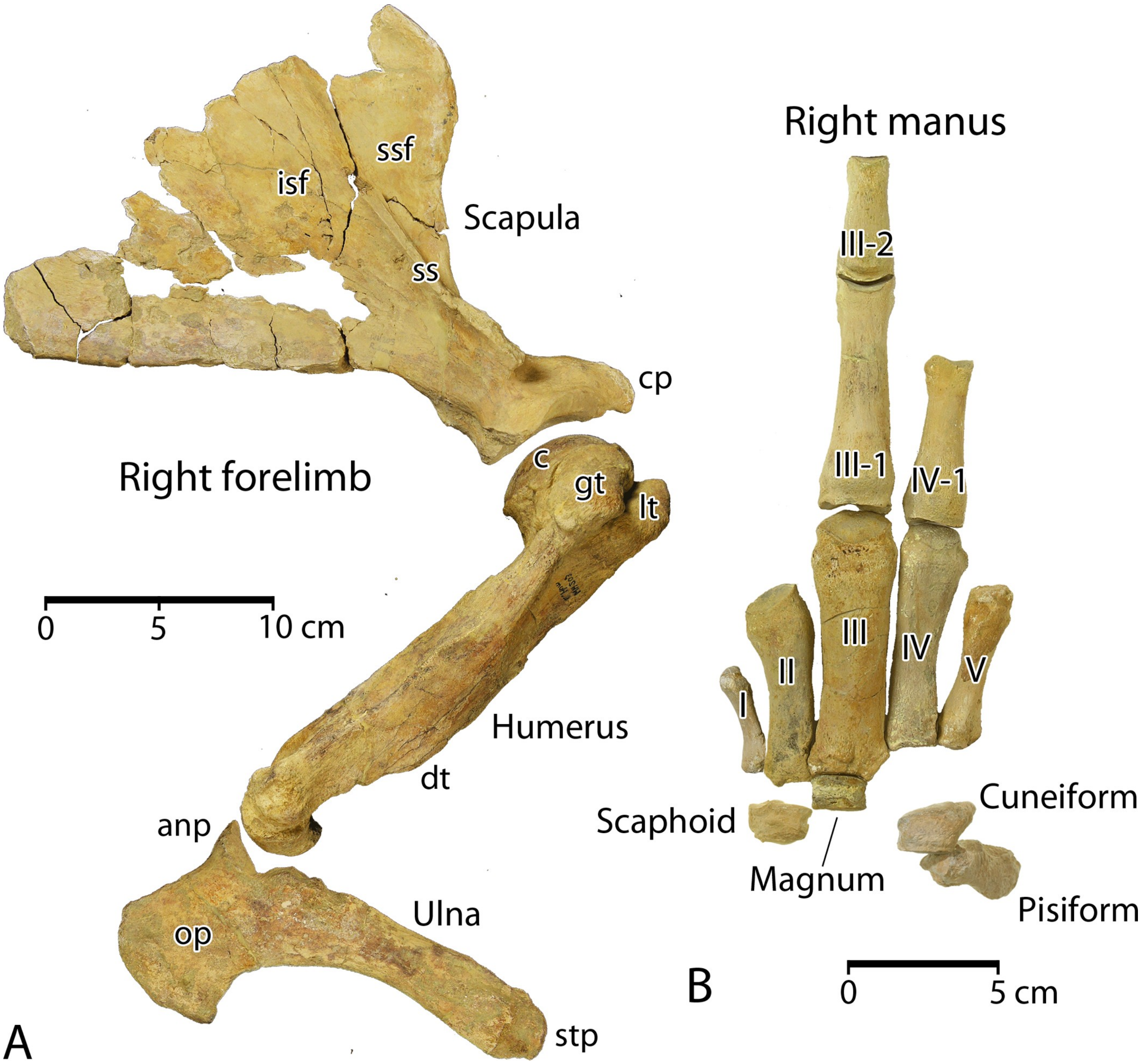


**Fig 14. Right forelimb and manus of *Aegicetus gehennae* type specimen (CGM 60584).** A, right forelimb with scapula, humerus, and ulna (reversed from left side) in right lateral view. B, partial right manus with the scaphoid, magnum, cuneiform, pisiform, metacarpals I through V, and associated proximal and middle phalanges in dorsal or right lateral view. Note dorsoventral expansion of the blade of the scapula, especially the infraspinous fossa (*isf*) and the prominent olecranon process (*op*) on the ulna. A and B have different scales. Abbreviations: *anp*, anconial process; *c*, caput; *cp*, coracoid process; *dt*, deltoid tuberosity; *gt*, greater tuberosity; *isf*, infraspinous fossa; *lt*, lesser tuberosity; *op*, olecranon process; *ss*, scapular spine; *ssf*, supraspinous fossa; *stp*, styloid process.

https://doi.org/10.1371/journal.pone.0225391.g014

facets on the capitulum are doubled on ribs R1 through R14, reflecting articulation with preceding and succeeding vertebrae. Ribs R1 through R14 also have a distinct rib tubercule with an articular facet separate from any on the capitulum. The greatest separation of the capitular and tubercular facets is on rib R1, and this separation decreases from R1 through R14. The anteroposterior diameter of the ribs, measured at the midshaft, increases from R1 to a maximum at R6, and this diameter then decreases slightly. In contrast, the mesiolateral diameter of

Table 4. Measurements of the ribs in CGM 60584, type specimen of *Aegicetus gehennae*.

| Left ribs | | | | | | Right ribs | | | | | |
|---|---|---|---|---|---|---|---|---|---|---|---|
| **Rib** | **Arc length** | **Chord length** | **C–T sep.** | **MS a–p** | **MS m–l** | **Rib** | **Arc length** | **Chord length** | **C–T sep.** | **MS a–p** | **MS m–l** |
| **R1** | — | — | 49.0 | 12.1 | 23.6 | **R1** | 242.0 | 151.0 | 43.9 | 13.4 | 22.1 |
| **R2** | — | — | — | 13.3 | 22.6 | **R2** | 310.0 | 215.0 | 41.3 | 13.2 | 21.8 |
| **R3** | 417.0 | 275.0 | 40.9 | 12.5 | 23.9 | **R3** | 383.0 | 270.0 | 37.2 | 14.8 | 23.0 |
| **R4** | — | — | — | 14.0 | 20.6 | **R4** | 490.0 | 332.0 | 39.9 | 13.7 | 22.2 |
| **R5** | 533.0 | 360.0 | 38.4 | 16.3 | 20.2 | **R5** | — | — | 40.6 | 16.5 | 18.4 |
| **R6** | — | — | 38.0 | 17.3 | 18.1 | **R6** | — | — | 39.1 | 18.5 | 18.3 |
| **R7** | 502.0 | 385.0 | 34.4 | 15.3 | 18.0 | **R7** | — | — | 35.2 | 16.4 | 16.7 |
| **R8** | 572.0 | 395.0 | 34.8 | 15.6 | 19.4 | **R8** | — | — | 34.7 | 16.0 | 16.9 |
| **R9** | 581.0 | 408.0 | 33.8 | 15.2 | 18.0 | **R9** | — | — | 31.0 | 15.8 | 16.5 |
| **R10** | 550.0 | 407.0 | 33.1 | 15.0 | 18.0 | **R10** | 535.0 | 405.0 | 32.7 | 14.1 | 14.6 |
| **R11** | — | — | — | 14.6 | 16.2 | **R11** | — | — | — | 14.4 | 17.6 |
| **R12** | — | — | — | 13.3 | 16.1 | **R12** | 475.0 | 390.0 | 33.9 | 15.7 | 15.1 |
| **R13** | — | — | 20.8 | 14.2 | 17.6 | **R13** | — | — | 24.4 | 13.4 | 16.4 |
| **R14** | — | — | — | — | — | **R14** | — | — | 21.8 | 13.8 | 12.4 |
| **R15** | — | — | 0.0 | 16.4 | 9.9 | **R15** | 114.0 | 102.0 | 0.0 | 13.7 | 8.1 |

Measurements, in mm, are given for ribs (R). Arc length and chord length are reported for complete ribs only, measured from the capitulum to the distal extremity. Capitulum–tuberculum separation (C–T sep.) is measured from the center of the articular surface for each. Midshaft anteroposterior length (MS a–p) and midshaft mediolateral length (MS a–p) are measured at or near the midshaft for each rib.

https://doi.org/10.1371/journal.pone.0225391.t004

the ribs, again measured at the midshaft, decreases from R1 through R14, paralleling the decrease observed in separation of the capitulum and tuberculum. The distal ends of the ribs are largest on R2–R5, which are the four interpreted as connecting to sternebra St2.

**Forelimb**. The forelimb of *Aegicetus gehennae* (CGM 60584) includes left and right scapulae, left and right humeri, and left and right ulnae (Figs 14A, 15; links to three-dimensional images of the left humerus and ulna are provided here in Supporting Information [S1 Table]). Radii were not recovered. Measurements of forelimb elements are listed in Table 5. The humerus is a little longer than the scapula, and the ulna is much shorter than either the scapula or humerus.

The right scapula (Fig 14A) is broad and flat. It is notably broader anteroposteriorly than it is long proximodistally. A narrow ridge of bone, the scapular spine (*ss*), separates the supraspinous fossa (*ssf*) from the infraspinous fossa (*isf*). The former is narrower than the latter, comprising only 60 mm of the total anteroposterior breadth. The infraspinous fossa comprises 165 mm of the total breadth. The scapular blade narrows from 225 mm in breadth proximally to a neck of only 39 mm before flaring to form a broader distal glenoid cavity and massive coracoid process (*cp*). The latter measures 74 mm in the plane of the scapular blade. The glenoid cavity itself is shallow and measures approximately 54 mm long anteroposteriorly and 45 mm wide mesiolaterally. The scapular spine (*ss*) rises to a massive spinous process (*sp*), well-preserved on the left scapula (Fig 15), which continues distally to form the acromion. The two together, spinous process and acromion, rise some 30 mm above the scapular blade and form a prominent boss that is 100 mm in length proximodistally.

The right humerus is complete. The caput (*c*) or head is convex and measures 61 mm proximodistally by 55 mm transversely, which is much larger than the glenoid cavity on the scapula. A larger articular surface on the humerus means that it could move through a substantial range of motion relative to the scapula. The greater tuberosity (*gt*) rises above the head on the anterolateral side of the humerus. The lesser tuberosity (*lt*) is smaller and positioned

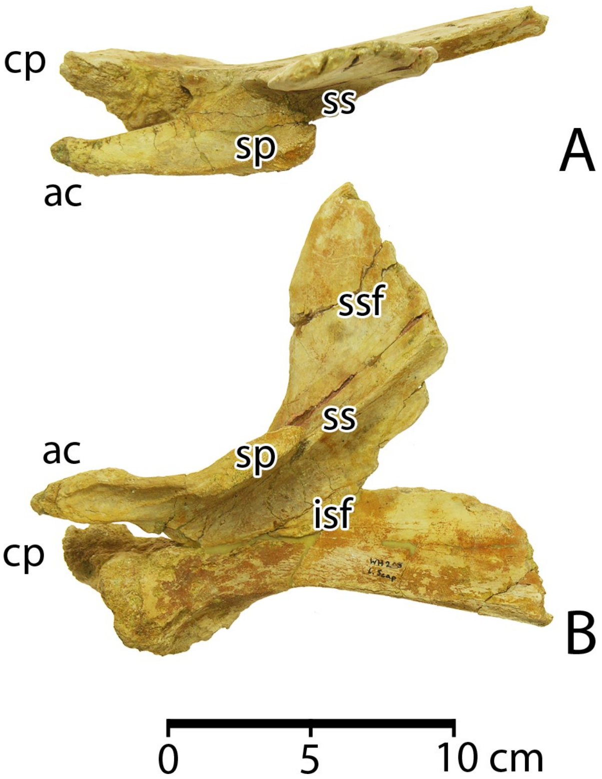


**Fig 15. Left scapula of *Aegicetus gehennae* type specimen (CGM 60584).** A, partial left scapula in dorsal view. B, partial left scapula in left lateral view. Note elongated acromion and distinct notch separating the posterior portion of the acromial process from the scapular spine. Abbreviations: *ac*, acromion; *cp*, coracoid process; *isf*, infraspinous fossa; *ss*, scapular spine; *sp*, spinous process; *ssf*, supraspinous fossa.

https://doi.org/10.1371/journal.pone.0225391.g015

anteromedially with respect to the head. The humeral shaft is robust, with the deltoid tuberosity (*dt*), marking the distal extent of the deltopectoral crest, located low on the anterior margin of the shaft. The trochlea for articulation of the ulna is oriented at a 45˚ angle to the rest of the humerus, meaning that the ulna flared laterally relative to the orientation of the humerus. There is no supratrochlear foramen perforating the distal end of the humerus, and there is no entepicondylar foramen.

The left ulna is complete (reversed in Fig 14), with a deep trochlear notch for articulation with the humerus. When articulated the ulna had an extensive range of motion from full extension in line with the humerus to full flexion bringing it approximately perpendicular to the humerus. The ulna is dominated by a very large olecranon process (*op*) extending almost perpendicularly from the proximal body of the bone. The body of the ulna is shallower, flaring only slightly near the robust styloid process (*stp*).

**Manus**. The manus of *Aegicetus gehennae* (CGM 60584) includes several identifiable bones from the left side, 12 identifiable bones from the right side (Fig 14B), and several unidentifiable bones. Measurements of manus elements are included in Table 5.

Four carpal bones can be identified in the right wrist. The scaphoid is a crescent-shaped bone with a single crescentic facet on the proximal surface and distinct facets for the trapezoid and trapezium on the distal surface. There is robust plantar process and the dorsomedial surface is smoothly curved. The magnum is a slightly wedge-shaped rectangular solid with proximal and distal articular facets. The latter matches the proximal facet on Mc-III precisely. The cuneiform is nearly the mirror image of the scaphoid, but it is more circular and has a single

Table 5. Measurements of bones of the forelimb and manus in CGM 60584, type specimen of *Aegicetus gehennae*.

| Element | P–d len. | Prox. A–p | Prox. M–l | MS a–p | MS m–l | Dist. A–p | Dist. M–l |
|---|---|---|---|---|---|---|---|
| Scapula | 210.0 | 225.0 | — | — | — | 57.0 | 45.0 |
| Humerus | 238.0 | 61.4 | 64.2 | 32.7 | 42.4 | 45.7 | 55.1 |
| Radius | 118.0* | — | — | — | — | — | — |
| Ulna | 138.7 | 80.8 | 29.8 | 24.9 | 15.7 | 32.1 | 19.6 |
| Trapezium | — | — | — | — | — | — | — |
| Scaphoid | 15.3 | — | — | 33.7 | 18.0 | — | — |
| Lunate | — | — | — | — | — | — | — |
| Cuneiform | — | — | — | — | — | — | — |
| Trapezoid | — | — | — | — | — | — | — |
| Magnum | 12.2 | — | — | 30.4 | 19.4 | — | — |
| Unciform | 19.9 | — | — | — | 24.1 | — | — |
| Pisiform | 51.6 | 17.7 | 17.0 | 12.6 | 19.9 | 12.8 | 20.1 |
| Mc-I | 37.2 | 16.8 | 10.4 | 11.0 | 6.7 | 16.5 | 9.0 |
| Mc-II | 66.5 | 19.1 | 17.0 | 15.3 | 13.7 | 22.6 | 18.9 |
| Mc-III | 88.3 | 28.0 | 26.4 | 21.2 | 16.5 | 29.0 | 23.8 |
| Mc-IV | 75.9 | 18.3 | 19.5 | 16.4 | 9.6 | 27.3 | 16.9 |
| Mc-V | 58.3 | 18.5 | 13.8 | 12.6 | 10.6 | 16.9 | 15.5 |
| M ph. I-1 | — | — | — | — | — | — | — |
| M ph. II-1 | — | — | — | — | — | — | — |
| M ph. III-1 | 72.7 | 24.5 | 27.8 | 17.0 | 17.3 | 19.4 | 16.0 |
| M ph. IV-1 | 54.5 | 21.9 | 21.1 | 13.8 | 13.3 | 16.8 | 11.2 |
| M ph. V-1 | — | — | — | — | — | — | — |
| M ph. I-2 | — | — | — | — | — | — | — |
| M ph. II-2 | — | — | — | — | — | — | — |
| M ph. III-2 | 41.6 | 19.7 | 16.9 | 14.7 | 11.8 | 14.0 | 10.1 |
| M ph. IV-2 | — | — | — | — | — | — | — |
| M ph. V-2 | — | — | — | — | — | — | — |

Measurements, in mm, include functional length proximodistally (P–d len.); proximal, midshaft, and distal anteroposterior or dorsoplantar length perpendicular to the proximal-distal length (Prox. a–p, MS a–p, and Dist. a–p, respectively); and proximal, midshaft, and distal mesiolateral width perpendicular to the proximal-distal length (Prox. m–l, MS m–l, and Dist. m–l, respectively). Abbreviations: *Mc*, metacarpal; *M ph*., manual phalanx. Estimates are marked with an asterisk.

https://doi.org/10.1371/journal.pone.0225391.t005

distal facet for the unciform. The proximal facet is divided for articulation with both the distal ulna and the proximal pisiform. The pisiform is a nondescript elongated bone with paired proximal articular surfaces for the unciform and the distal ulna.

Metacarpals increase in size from Mc-I to Mc-II to Mc-III and then decrease in size from Mc-III to Mc-IV to Mc-V. Mc-I is the smallest metacarpal. It has a rugose base with a single, smooth, flat, proximal facet for articulation with the trapezium in the wrist. The shaft is curved, and there is a single, convex, distal articular facet for a proximal phalanx. Mc-II is one of the three large, robust, central metacarpals. It has a large triangular proximal facet for articulation with the trapezoid in the wrist, a concave lateral facet for articulation with the base of Mc-I, and lateral facets for articulation with the magnum and Mc-III. The shaft curves slightly disto-medially, and there is an enlarged distal end with a curved facet for articulation with the corresponding proximal phalanx.

Mc-III is a massive, relatively-straight metacarpal with a nearly rectangular proximal articulation for the magnum. Mc-III is the longest and most robust metacarpal. It is flanked laterally by a triangular facet for articulation with the unciform. Medially there is close-fit surface for

articulation with Mc-II, and laterally there is a similar close-fit surface for articulation with Mc-IV. The distal articulation is convex for the corresponding proximal phalanx. Plantar to this there is a median keel that separated two relatively large sesamoid bones in life.

Mc-IV is nearly as long as Mc-III, but it is less robust. It has a triangular proximal facet for the cuneiform that mirrors the triangular Mc-II facet for the trapezoid. Medially there is a contact facet for articulation with Mc-III. Laterally there is a depression to receive a projection of Mc-V. The body of Mc-IV curves distolaterally, and it is flattened distally. There is a convex articulation for the corresponding proximal phalanx, and the median keel separating sesamoids flares laterally. Mc-V is larger than Mc-I, but smaller than all three central metacarpals. It has a small flat surface for articulation with the unciform and the aforementioned projection for contact with Mc-IV. The body is relatively straight and the distal articulation for the corresponding proximal phalanx nearly flat.

The only proximal phalanges identified with confidence are the very large phalanx III-1 and the smaller phalanx IV-1. Both have concave proximal articulations, with plantar notches for the plantar keel on the distal ends of Mc-III and Mc-IV. Both are swollen dorsally just distal to their metacarpal articulation, and both have paired swellings on their plantar surface where they were bound to the intervening sesamoids. Both proximal phalanges taper distally to a saddle-shaped articulation for the corresponding middle phalanx. There is no evidence for interosseous muscles or webbing connecting adjacent proximal phalanges. The only middle phalanx identified with confidence is III-2, which has a concave proximal articulation that fits the saddle-shaped distal articulation on III-1. Phalanx III-2 tapers distally and ends in a simple convex oval surface for articulation with phalanx III-3.

**Hind limb**. The hind limb of *Aegicetus gehennae* (CGM 60584) includes left and right innominates, left and right femora, a left tibia, and parts of both fibulae (Fig 16A; a link to three-dimensional images of the left innominate, left femur, and right tibia are provided here in Supporting Information [S1 Table]). Measurements of hind limb elements are listed in Table 6.

The innominate (Fig 16A) is a large bone, measuring 417 mm in total length. Ilium (*Il*, length 235 mm), ischium (*Is*, length 185 mm), and pubis (*Pu*, length 155 mm) meet near the center of the acetabulum (*ac*) where they are solidly co-ossified. The acetabulum is circular, 36 mm in diameter, and some 13 mm deep. A 38 mm diameter sphere fits the curvature of the articular surfaces. The acetabular fossa is 6–7 mm deep within the acetabulum and opens posteroventrally into the obturator foramen (*of*) through a well-developed acetabular notch.

The ilium is a rod-like bone that is 27.1 mm wide mediolaterally and 72.0 mm deep dorsoventrally at its anterior or proximal end. It then narrows to a minimum of 20.8 mm in width and 26.6 mm in depth before flaring again at the acetabulum. The ventral iliac spine (*is*) is preserved but the dorsal iliac crest is damaged. A prominent ridge of bone runs along the ventral margin of the lateral surface of the proximal ilium, delimiting a gluteal fossa (*gf*). The medial surface of the left ilium is smooth, and there is no indication of an auricular surface for articulation with an auricular process of the first sacral vertebra.

The ischium forms the dorsal portion of the distal innominate. The ischial ramus is rod-like above the obturator foramen (*of*), where it is oval in cross section and measures minimally 15.3 mm in width and 19.3 mm in depth. The ischium flares distally into a broad table of bone with the ischiatic tuberosity (*it*) forming the distolateral corner and part of the distal edge of the innominate. Bone of the distal ischiatic arch is not well preserved and some bone is missing that connected the ischium to the pubis in life, closing the obturator foramen.

The pubis is rod-like below the obturator foramen, but differs from the ischium in having a prominent ventral keel of bone throughout its length. The pubic ramus measures minimally 16.9 mm in width and 25.0 mm in depth. Distally the pubis flares into a sheet of bone. The pubis has an oval, slightly-roughened articular surface for its opposite, and this symphysis

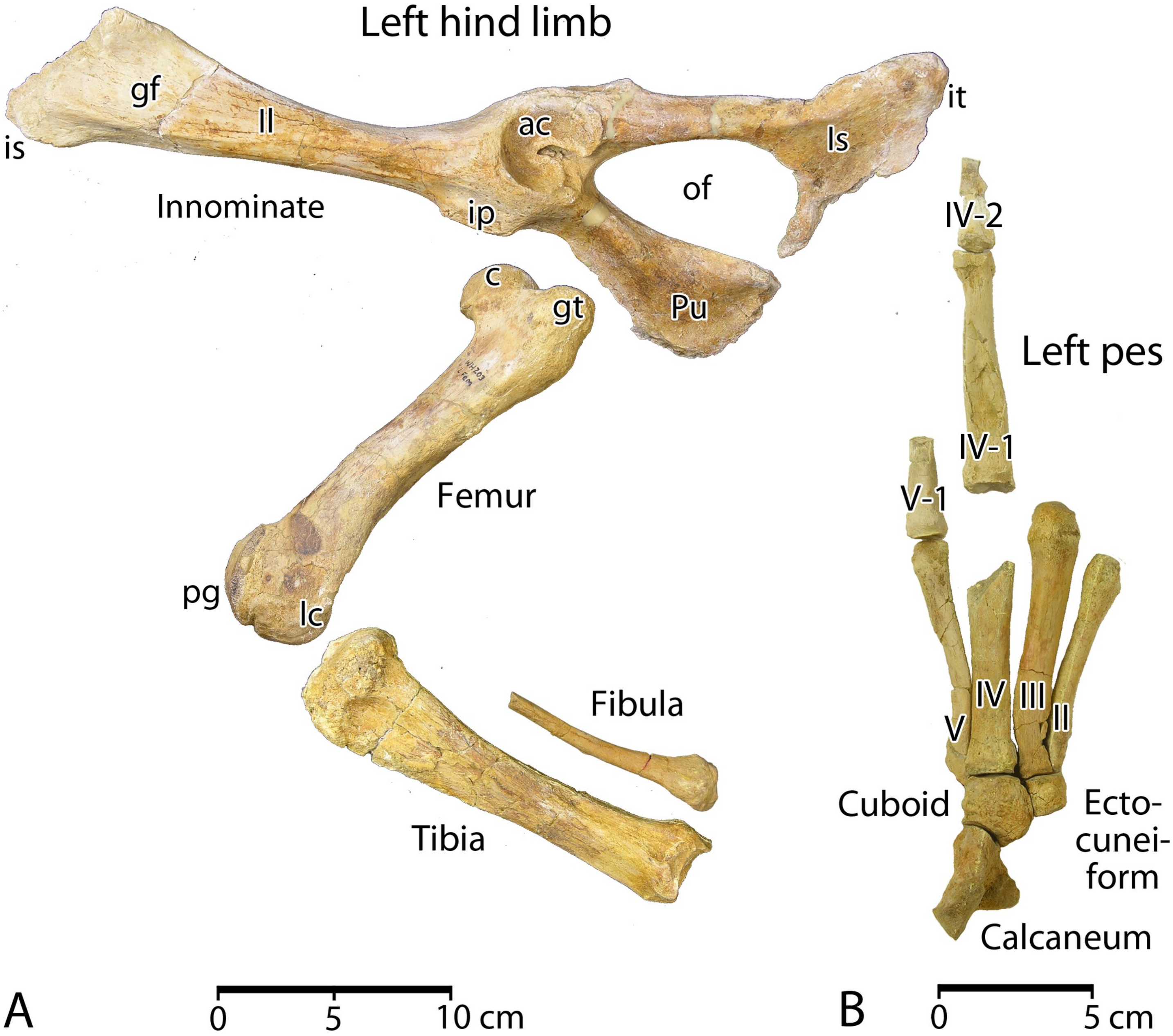


**Fig 16. Left hind limb and pes of *Aegicetus gehennae* type specimen (CGM 60584).** A, innominate, femur, tibia (reversed from right side), and partial fibula of the left hind limb in left lateral view. B, calcaneum, cuboid, ectocuneiform, metatarsals II through V, and associated proximal and middle tarsal phalanges. A and B have different scales. Note that there is no posterolateral notch in the cuboid for the anterior process of the calcaneum, a notch found in artiodactyls and earlier protocetids. Abbreviations: *ac*, acetabulum; *c*, caput; *gf*, gluteal fossa; *gt*, greater trochanter; *lc*, lateral condyle; *Il*, Ilium; ip, iliopectineal eminence; *Is*, ischium; *is*, iliac spine; *it*, ischial tuberosity; *of*, obturator foramen; *pg*, patellar groove; *Pu*, pubis.

https://doi.org/10.1371/journal.pone.0225391.g016

borders the medial surface of the ventral margin of the pubis. The pubic symphysis is about 54.0 mm long anteroposteriorly and 22 mm deep dorsoventrally.

The femur is robust, especially at the distal end. The caput (*c*) or head of the femur is spherical and relatively small, measuring 38 mm in maximum diameter. It has a well developed fovea capitis femoris (*fc* in Fig 18 below) for a round ligament posterior to and a little dorsal relative to the center of the articular surface. There is a prominent greater trochanter (*gt*)

**Table 6. Measurements of bones of the hind limb and pes in CGM 60584, type specimen of *Aegicetus gehennae*.**

| Element | P–d len. | Prox. A–p | Prox. M–l | MS a–p | MS m–l | Dist. A–p | Dist. M–l |
|---|---|---|---|---|---|---|---|
| Innominate | 417.0 | — | — | — | — | — | — |
| Femur | 208.0 | 35.9 | 67.6 | 30.7 | 35.9 | 59.5 | 49.0 |
| Tibia | 178.0 | 52.2 | 45.1 | 26.3 | 21.6 | 35.4 | 34.2 |
| Fibula | — | — | — | 8.3 | 8.7 | 23.4 | 14.5 |
| Astragalus | — | — | — | — | — | — | — |
| Calcanium | 36.3 | — | — | — | — | — | — |
| Cuboid | 19.9 | — | — | 32.4 | 24.2 | — | — |
| Navicular | 7.1 | — | — | 30.2 | 16.9 | — | — |
| Ectocuneiform | 12.6 | — | — | 18.8 | 13.6 | — | — |
| Mesocuneiform | — | — | — | — | — | — | — |
| Entocuneiform | — | — | — | — | — | — | — |
| Mt-I | — | — | — | — | — | — | — |
| Mt-II | 76.6 | 15.2 | 9.8 | 7.2 | 8.5 | 13.4 | 12.1 |
| Mt-III | 93.5 | 28.0 | — | 9.4 | 12.1 | 17.7 | 19.4 |
| Mt-IV | — | 26.7 | 17.0 | 11.1 | 13.1 | — | — |
| Mt-V | 83.7 | 12.7 | 7.3 | 8.2 | 5.3 | 15.7 | 12.4 |
| P ph. I-1 | — | — | — | — | — | — | — |
| P ph. II-1 | — | — | — | — | — | — | — |
| P ph. III-1 | — | — | — | — | — | — | — |
| P ph. IV-1 | 77.1 | 16.0 | 17.8 | 7.7 | 12.7 | 9.7 | 13.6 |
| P ph. V-1 | — | 13.3 | 14.5 | 7.3 | 8.4 | — | — |
| P ph. I-2 | — | — | — | — | — | — | — |
| P ph. II-2 | — | — | — | — | — | — | — |
| P ph. III-2 | — | — | — | — | — | — | — |
| P ph. IV-2 | — | 11.0 | 13.3 | 4.8 | 9.6 | — | — |
| P ph. V-2 | — | — | — | — | — | — | — |

Measurements, in mm, include proximal–distal length (P–d len.); proximal, midshaft, and distal anteroposterior or dorsoplantar length perpendicular to the proximal-distal length (Prox. a–p, MS a–p, and Dist. a–p, respectively); and proximal, midshaft, and distal mesiolateral width perpendicular to the proximal-distal length (Prox. m–l, MS m–l, and Dist. m–l, respectively). Abbreviations: *Mt*, metatarsal; *P ph*., pedal phalanx.

https://doi.org/10.1371/journal.pone.0225391.t006

lateral to and rising above the femoral head. The lesser trochanter (*lt*) on the medial surface of the femur is smaller but also prominent. Both trochanters are connected by a trochanteric crest on the posterior surface of the femur. This crest encloses a relatively large and deep trochanteric fossa (*tf*). The trochanteric fossa opens posteromedially behind the femoral head. The body of the femur thickens distally to support a wide patellar groove anteriorly, and large medial and lateral femoral condyles (*mc* and *lc*) posteriorly. The entire patellar surface is about 46.5 mm long proximodistally and 24.5 mm wide mesiolaterally. The medial condyle is 32.7 mm long proximodistally and 21.3 mm wide mesiolaterally. The lateral condyle is similar in size at 32.7 mm long and 25.0 mm wide. Comparison of orientation of the femoral head and greater trochanter with orientation of the femoral condyles shows some 30° in torsion in the long axis of the femur, which forces femoral condyles to face posterolaterally rather than posteriorly.

The tibia is a little shorter than the femur, and it differs in being straight and untorted. The tibial plateau has a central intercondylar eminence flanked by articular facets for the medial and lateral condyles of the femur. The tibial crest is prominent on the anterior surface of the tibia, and this extends from the tibial plateau down the upper half of the tibial body. The distal

end of the tibia has an astragalar facet that is nearly square. This is bordered medially by a relatively small medial malloleus and laterally by a well-developed lateral facet for articulation with the distal fibula. Low ridges of bone mark the anterior and posterior borders of the astragalar facet. The facet itself is smoothly curved, indicating that the body of the astragalus was similarly curved with a shallow proximal trochlea.

The right fibula (not shown) has a poorly preserved articulation for the proximal tibia and much of a relatively thin, cylindrical midshaft. The left tibia has an enlarged distal end and a portion of a similarly thin cylindrical midshaft. The distal end of the left fibula has a smooth, flat articular surface on the medial side of the shaft for articulation with the tibia, and a larger curved facet on the distal end for articulation with the curved fibular facet of the calcaneum.

**Pes**. The pes of *Aegicetus gehennae* (CGM 60584) includes several identifiable bones from the left side, 10 identifiable bones from the right side (Fig 16B), and several unidentifiable bones. The astragalus of CGM 60584 was not found. Measurements of pes elements are included in Table 6.

The body of the calcaneum is shown in Fig 17A and 17B. The calcaneal tuber is missing due to breakage. The calcaneum is unusual in having the distal cuboid facet (*cu*) on the side of the body opposite the proximal fibular facet (*fi*; see Fig 17D). The calcaneal tuber occupies the posterior surface of the calcaneum between the proximal fibular facet and the distal cuboid facet. Artiodactyls and primitive protocetids, in contrast, have the distal cuboid facet opposite the calcaneal tuber, and the fibular facet, oval in outline and convex, occupies the anterior or dorsal surface of the calcaneum between the two.

The fibular facet is confluent with the ectal facet (ec) for articulation with the astragalus. The ectal facet is on the medial surface of the calcaneum. It is similarly oval in outline, but nearly flat. The sustentacular facet (*su*) is small, projecting from the medial surface of the calcaneum for articulation with the astragalus. Calcanea of protocetids often have a third more-anterior facet on the medial surface for articulation with the astragalus, but in *Aegicetus* this third astragalar (*as*) facet is farther forward and on the cuboid rather than the calcaneum. The cuboid facet (*cu*) on the calcaneum is actually two confluent, slightly-convex, and more-or-less circular facets. These are as wide as the calcaneum and cuboid, and oriented transversely. The calcaneum is not inserted into a notch in the posterolateral surface of the cuboid as it is in artiodactyls and other protocetid archaeocetes.

The cuboid (Fig 17B) is a rectangular solid with its longest axis paralleling the longest axis of the calcaneum. Calcaneal facets on the cuboid are two confluent, slightly concave, and more-or-less circular facets matching their cuboid facet (*cu*) counterparts on the calcaneum. The cuboid does not have the posterolateral notch for the calcaneum seen in artiodactyls and other protocetid archaeocetes. There are two astragalar facets (*as*) on the posteromedial surface of the cuboid for articulation with the head of the astragalus. The lower of the two is confluent with the navicular facet (*na*) on the medial surface of the cuboid. An ectocuneiform facet (*ecf*) is present on the medial surface just anterior to the astragalar and navicular facets. The distal surface of the cuboid has a large, deep metatarsal facet (*mt*) for articulation with the base of metatarsal IV, and a smaller facet dorsal and lateral to this (not shown) for articulation with metatarsal V.

In addition to the left tarsal bones shown in Figs 15B and 16, CGM 60584 preserves a right navicular (Fig 16C). This is a proximodistally short crescentic bone with a smoothly convex medial surface and a small distally-projecting bony process at the base. A large smoothly-curved facet covers most of the proximal surface for articulation with the astragalus. The distal surface has facets for articulation with the ectocuneiform above and mesocuneiform below. The lateral surface of the navicular has a small facet near the base for articulation with the cuboid.

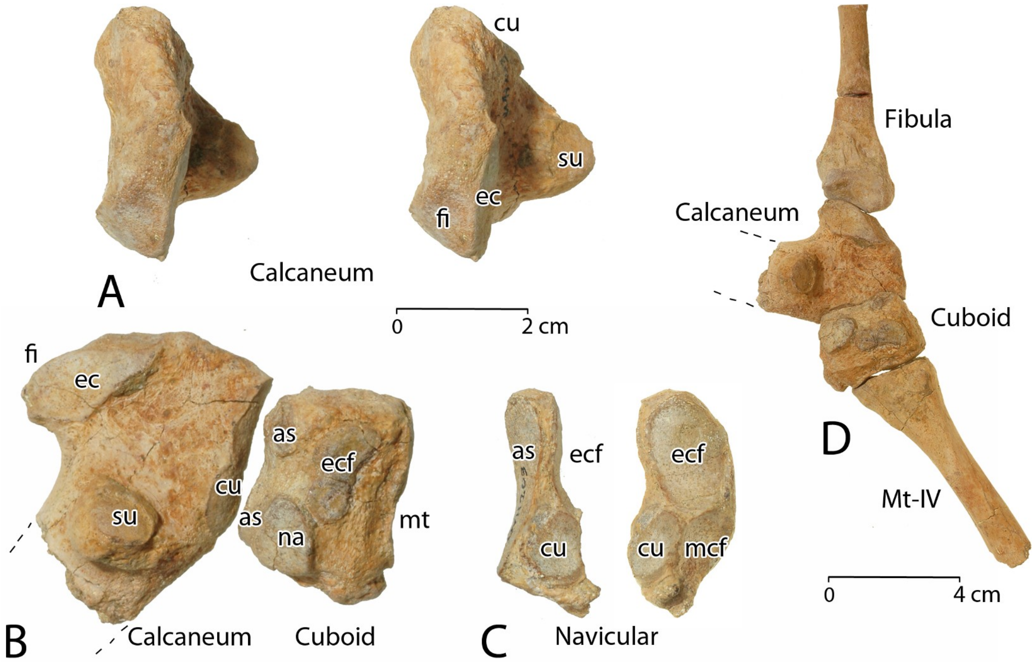


**Fig 17. Tarsal bones of *Aegicetus gehennae* type specimen (CGM 60584).** A, left calcaneum in anterodorsal view (stereophotographs). B, left calcaneum and cuboid in medial view, positioned as in normal mammals where the cuboid is anterior to the calcaneum. C, right navicular in medial and anterior views. D, left fibula, calcaneum, cuboid, and metatarsal Mt-IV in medial view. Articulation of these four elements, flexed at the ankle joint between the fibula and calcaneum, shows near alignment of the pes with the lower leg in *Aegicetus*. Dashed lines show the orientation of the missing calcaneal tuber. There is no posterolateral notch in the cuboid for the anterior process of the calcaneum, a notch found in artiodactyls and earlier protocetids. Abbreviations: *as*, astragalar facet; *ec*, ectal facet for articulation with the astragalus; *ecf*, ectocuneiform facet; *cu*, cuboid facet; *fi*, fibular facet; *mcf*, mesocuneiform facet; mt, metarsal facet for Mt-IV; *na*, navicular facet; *su*, sustentacular facet for articulation with the astragalus.

https://doi.org/10.1371/journal.pone.0225391.g017

The ectocuneiform (Fig 16B) is a short cylindrical bone with flat ends and one flat side. Each bears an articular facet. The flat proximal surface articulates with the navicular, and the flat distal surface articulates with the base of metatarsal Mt-III. The remaining flat surface is the lateral surface, where the articular facet matches that on the cuboid. In addition, there is a small facet at the base of the Mt-III facet on the ectocuneiform that is presumably for the mesocuneiform (not found).

The most medial of the metatarsals, Mt-II (Fig 16B), is a long, relatively narrow bone with a wedge-shaped proximal base. The base has three articular facets. The largest of these facets, on the lateral surface of the base, is a contact facet for articulation with Mt-III. The smallest articular surface, a convexity on the proximal end of Mt-II, probably contacted the mesocuneiform. The third articular facet, a narrow facet on the medial surface of the base seems to indicate the presence of at least a rudimentary hallucial Mt-I (not found). The midshaft of Mt-II is cylindrical and flattened where pressed against Mt-III. The distal end has a smoothly-rounded articular surface for the corresponding proximal phalanx (not found). The position and curvature of the distal articular facet indicates that there was limited flexion and extension of the proximal phalanx relative to the long axis of Mt-II.

Metatarsal Mt-III (Fig 16B) is one of the two longest and largest metatarsals. It has a transversely narrow and dorsoventrally deep proximal base with a prominent flexor tubercle. The proximal surface has a slightly concave articular facet for the ectocuneiform. The medial surface is damaged at the base, obscuring the articular surface for Mt-II. The lateral surface at the

base of Mt-III has two distinct facets, well separated, for articulation with Mt-IV. The midshaft of Mt-III is elliptical in cross section, where it is wider than deep. Mt-III has an expanded distal end with a smoothly-rounded articular surface for the corresponding proximal phalanx, like that on Mt-II, but here there is a prominent median keel on the plantar portion of the articular surface, which was clearly flanked by sesamoids in life. Here again, it appears that there was limited flexion and extension of the corresponding proximal phalanx (not found) relative to the long axis of Mt-III.

Metatarsal Mt-IV (Fig 16B) is the second of the two largest metatarsals. It is broken, but was probably longer than Mt-III. Mt-IV, like Mt-III, has a transversely-narrow and dorsoventrally-deep proximal base with a prominent flexor tubercle. The proximal surface has a slightly concave articular facet for articulation with the cuboid. The medial surface at the base of Mt-IV has two distinct facets, well separated, for articulation with Mt-III. The lateral surface at the base is concave with a contact surface for Mt-V. The midshaft is elliptical in cross section, and again wider than deep. The distal end of Mt-IV is missing.

Metatarsal Mt-V (Fig 16B) is a long, relatively narrow bone, mirroring Mt-II in this respect. The proximomedial surface of Mt-V has a small slightly convex articular facet for articulation with the cuboid, and there is a longer medial contact surface for Mt-IV. The midshaft is elliptical in cross section, and here narrower than it is deep. The distal end of Mt-V has a smoothly-rounded articular surface for the corresponding proximal phalanx. Here again, the position and curvature of the distal articular facet indicates that flexion and extension of the proximal phalanx was limited relative to the long axis of Mt-V.

The only complete proximal phalanx is IV-1 (Fig 16B), which is long, narrow, and dorsoplantarly thin. The proximal end is shallowly concave to receive the distal end of Mt-IV. The distal end of phalanx IV-1 has a saddle-shaped articulation for phalanx IV-2. The proximal end of phalanx V-1 is like that of phalanx IV-1, and the midshaft was similarly narrow and thin. There is no evidence for interosseous muscles or webbing that might have connected adjacent proximal phalanges. The only middle phalanx is IV-2, which is broken in ways that preclude any detailed description.

When the left fibula, calcaneum, cuboid, and metatarsal Mt-IV of *Aegicetus gehennae* are articulated (Fig 16D), it is clear that the foot was habitually oriented in near alignment with the lower leg. There is little of the ankle flexion and extension required of a foot mediating movement on a rigid terrestrial substrate, and there is little of the ankle flexion and extension required for foot-powered swimming.

## Sexual dimorphism

*Aegicetus gehennae* is known from two specimens, the initial specimen CGM 60583 found at locality WH2007-031 (Fig 2), and the holotype CGM 60584 found at locality WH-203 (Fig 2). CGM 60583 is an extremely-weathered, associated partial skeleton, for which the only elements worth collecting were a left femur and a right tibia (Fig 18). Where preserved, epiphyses of CGM 60583 are fused indicating that the specimen was mature. The holotype is the unusually complete skeleton described and illustrated here. Here too epiphyses are fused indicating that the specimen was mature. These are conservatively grouped together in the same species because they are similar in size and form, because they were found within a few kilometers of each other in the same strata, and because they are the only protocetid specimens among hundreds of partial skeletons of archaeocetes known from Wadi Al Hitan.

The femur of CGM 60583 is 184 mm in length, measured from the notch between the femoral head and greater trochanter to the medial condyle. Maximum diameter of the femoral head is 32.9 mm, and maximum transverse diameter of the femoral midshaft is 32.1 mm.

Comparable measurements for CGM 60584 are 200 mm, 37.3 mm, and 37.3 mm, respectively. The only measurement that can be made reliably on the tibia is the width of the facet on the distal tibia for articulation with the astragalus: 22.5 mm. The comparable measurement for CGM 60584 is 24.5.

Measurements of the holotype CGM 60584 are consistently larger than measurements of the referred specimen CGM 60583. The differences range from 9% larger to 16% larger, with a median difference of 11%. Differences on a natural-log scale range from 0.08 to 0.15 with a median of 0.11 natural log units. These percentage and natural-log differences are closely similar to the differences found in comparing male and female *Maiacetus inuus* (12% and 0.11 natural log units [5]). Thus it is reasonable, given the evidence at hand, to interpret CGM 60853 as a female and CGM 60584 as a male of the same genus and species.

## Body weight

The lengths, widths, and heights of core vertebral centra, considered together, provide conservative estimates for the body weights of archaeocete cetaceans [43]. A focus on core vertebrae (generally thoracic through anterior lumbar vertebrae) minimizes the influence that more specialized, and often more variable, portions of the skeleton have on body weight. Cranial size and tooth size is influenced by diet as well as body weight. Forelimb size, hind limb size, and tail size are all influenced by locomotor specializations. A focus on core vertebrae has the additional advantage of enabling estimation when skeletons are less than complete. Here the overall body weight of *Aegicetus gehennae* is estimated as a median of individual weights, each predicted from multiple regression of weight on centrum length, width, and height, for successive core vertebrae.

Vertebral profiles for eight extant cetacean species and five extant pinniped species are illustrated in Fig 19. Each has an associated body weight. Focus on the core portion for skeleton, enclosed in dashed lines in Fig 19, avoids the highly variable size of cervical vertebrae associated with neck length, and the highly variable size of posterior lumbar, sacral, and caudal vertebrae associated with overall skeletal length. Baseline data involved in construction of Fig 19 were provided by Gingerich [43]. When combined with vertebral measurements for male *Aegicetus gehennae* (Table 2), we calculate a series of estimates beginning with 1,043 kg based on the length, width, and height of thoracic T1, and continuing through 1,026 kg based on the length, width, and height of lumbar L4 (Fig 19). The median for these 11 estimates is 891 kg. This estimate, 890 kg when rounded, is substantially greater than estimates for early middle Eocene protocetids such as *Artiocetus clavis* (400 kg), *Rodhocetus balochistanensis* (410 kg), and *Maiacetus inuus* (390 kg) [5]. An estimate of 890 kg for *Aegicetus gehennae* is a little less than estimates for body weights of late Eocene basilosaurids such as *Zygorhiza kochii* (1,000 kg) and *Dorudon atrox* (1130 kg [43]).

# Discussion

*Aegicetus gehennae* is important for four reasons: (1) *A. gehennae* extends the stratigraphic range of Protocetidae into the earliest Priabonian age of the geological time scale (earliest late Eocene). (2) *A. gehennae* is the most complete representative of Georgiacetinae within Protocetidae, increasing our understanding of the morphology of constituent georgiacetines. (3) *A. gehennae* groups with other protocetids on a spectrum of semiaquatic mammalian skeletal proportions, but it was more aquatic than most protocetids in lacking any firm connection of the pelvis and hind limbs to the vertebral column. Finally, (4) posterior thoracic through anterior caudal elongation indicates that *A. gehennae* swam by mid-body through tail undulation. Each of these is considered in turn.

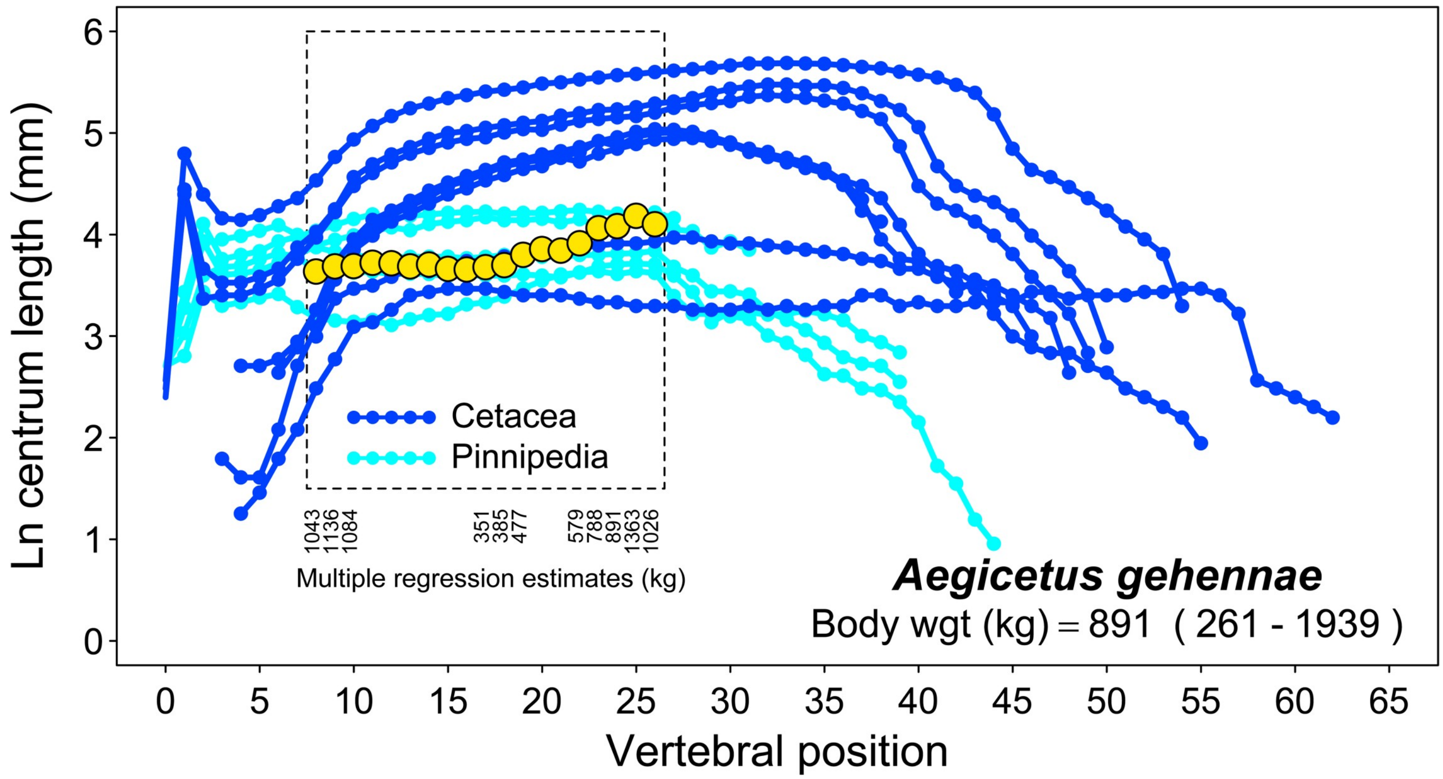


**Fig 19. Body weight estimate for *Aegicetus gehennae*.** The preferred estimate of 891 kg (rounded to 890 kg) is the median of 11 independent multiple regressions of body weight on centrum length, width, and height for vertebrae 8 through 26 (T1 through L4; Table 2). Colored lines are traces of vertebral centrum length for eight extant cetacean and five extant pinniped taxa forming the reference set (corresponding centrum widths and heights are not shown). Yellow circles show centrum lengths for *A. gehennae*. Dashed rectangle encloses core vertebrae of interest for body weight prediction. Note that the ordinate is on a proportional scale (original measurements are in mm). See [43] for further explanation and documentation.

https://doi.org/10.1371/journal.pone.0225391.g019

## Stratigraphic range of Protocetidae

Twenty-two species of Protocetidae are now known, 13 species of Protocetinae, one species of Makaracetinae, and 8 species of Georgiacetinae (Table 7). Protocetinae and Makaracetinae are all Lutetian in age, early middle Eocene. Most Georgiacetinae are Bartonian in age, late middle Eocene. The one exception is *Aegicetus gehennae* described here, which extends the range of Georgiacetinae into the early Priabonian.

The *Aegicetus gehennae* type locality east of Garet Gehannam in the Wadi Al-Hitan World Heritage Site (Fig 2) is near the base of the Gehannam Formation glauconitic mudstones of Priabonian, late Eocene, age. It is just above the top of the El Gharaq Formation nummulitic limestone of Bartonian, middle Eocene, age (Fig 3). These formations are separated by a disconformity related to the global end-Bartonian and beginning-Priabonian low sea stand (Pr-1 of Fig 4). *Aegicetus gehennae* is the latest-surviving protocetid known at present.

## *Aegicetus* and the morphology of Georgiacetinae

The type specimen of *Aegicetus gehennae* is the most complete georgiacetine skeleton known, and one of the most complete of all protocetids. The most complete georgiacetine known previously is that of *Georgiacetus vogtlensis* described by Hulbert and others [2, 32]. *G. vogtlensis* has a virtually complete cranium, both dentaries, 23 vertebrae, a caudal chevron, 12 ribs, one sternebra, and left and right innominates. By comparison *A. gehennae* has a partial cranium,

**Table 7. Temporal and geographic distribution of 21 genera and 22 named species of Eocene Protocetidae.**

| Genus and species | Reference | Holotype | Age | Type locality | N latitude | E longitude |
|---|---|---|---|---|---|---|
| **Protocetinae** | | | | | | |
| *Protocetus atavus* | Fraas, 1904: 201 [44] | SMNS 110847 | M. Lutetian | Gebel Mokattam (Egypt) | 30.03528 | 31.27222 |
| *Indocetus ramani* | Sahni and Mishra, 1975: 18 [45] | LUVP 11034 | L. Lutetian | Rato Nala (India) | 23.50550 | 68.68750 |
| *Rodhocetus kasranii* | Gingerich et al., 1994: 844 [1] | GSP-UM 3012 | E. Lutetian | Bozmar Nadi (Pakistan) | 30.77333 | 70.44611 |
| *Takracetus simus* | Gingerich et al., 1995b: 300 [46] | GSP-UM 3041 | E. Lutetian | Takra (Pakistan) | 30.14392 | 70.36388 |
| *Gaviacetus razai* | Gingerich et al., 1995b: 305 [46] | GSP-UM 3095 | M. Lutetian | Basti Ahmed (Pakistan) | 30.12581 | 70.36526 |
| *Artiocetus clavis* | Gingerich et al., 2001b: 2242 [4] | GSP-UM 3458 | E. Lutetian | Kunvit (Pakistan) | 30.09565 | 69.78858 |
| *Rodho. Balochistanensis* | Gingerich et al., 2001b: 2242 [4] | GSP-UM 3485 | E. Lutetian | Kunvit (Pakistan) | 30.08902 | 69.79403 |
| *Qaisracetus arifi* | Gingerich et al., 2001a: 295 [3] | GSP-UM 3410 | L. Lutetian | Ander Dabh Shumali (Pak.) | 30.90983 | 70.22950 |
| *Maiacetus inuus* | Gingerich et al., 2009: 4 [5] | GSP-UM 3475 | E. Lutetian | Kunvit (Pakistan) | 30.09630 | 69.79080 |
| *Aegyptocetus tarfa* | Bianucci and Gingerich, 2011: 1175 [35] | MSNTUP I-15459 | L. Lutetian | Khashm el-Raqaba (Egypt) | 28.45100 | 31.83400 |
| *Kharodacetus sahnii* | Bajpai and Thewissen, 2014: 6 [47] | VPL 1021 | L. Lutetian | Rato Nala (India) | 23.52861 | 68.68556 |
| *Dhedacetus hyaeni* | Bajpai and Thewissen, 2014: 10 [47] | IITR-SB 2870 | L. Lutetian | Dhedadi (India) | 23.77833 | 68.78778 |
| *Peregocetus pacificus* | Lambert et al., 2019: 1 [7] | MUSM 3580 | L. Lutetian | Media Luna (Peru) | −14.60408 | −75.91347 |
| **Makaracetinae** | | | | | | |
| *Makaracetus bidens* | Gingerich et al., 2005: 197 [24] | GSP-UM 3570 | Late Lutetian | Kunvit (Pakistan) | 30.11203 | 69.80356 |
| **Georgiacetinae** | | | | | | |
| *Pappocetus lugardi* | Andrews, 1920: 309 [26] | NHML M 11414 | Bartonian | Ameke (Nigeria) | 5.55540 | 7.51520 |
| *Babiacetus indicus* | Trivedy and Satsangi, 1984: 322 [30] | GSI 19647 | E. Bartonian | Babia Hill (India) | 23.70000 | 68.77500 |
| *Georgiacetus vogtlensis* | Hulbert et al., 1998: 912 [2] | GSM 350 | E Bartonian | Plant Vogtle (U.S.A.) | 33.14861 | −81.75580 |
| *Natchitochia jonesi* | Uhen, 1998: 664 [6] | USNM 16805 | E. Bartonian | Natchitoches (U.S.A.) | 31.69290 | −93.08000 |
| *Carolinacetus gingerichi* | Geisler et al., 2005: 6 [34] | ChM PV 5401 | M. Bartonian | 'Cross Quarry' (U.S.A.) | 33.35000 | −80.33222 |
| *Crenatocetus rayi* | McLeod and Barnes, 2008: 76 [25] | USNM 392014 | Bartonian | New Bern Q. (?) (U.S.A.) | 35.14583 | −77.08417 |
| *Tupelocetus palmeri* | Gibson et al., 2019: 2 [48] | ChM PV6950 | Bartonian | Martin Marietta Q. (U.S.A.) | 33.34444 | −80.23027 |
| *Aegicetus gehennae* | Gingerich et al., this study | CGM 60584 | E. Priabonian | Wadi Al Hitan (Egypt) | 30.16994 | 29.31770 |

Three subfamilies are represented: Protocetinae, Makaracetinae, and Georgiacetinae (table updated from [24]).

https://doi.org/10.1371/journal.pone.0225391.t007

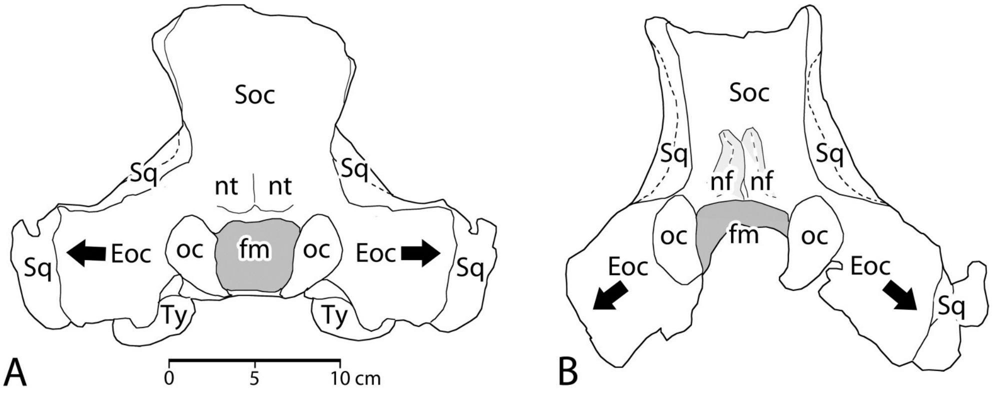


**Fig 20. Occiput of *Georgiacetus vogtlensis* (A) compared to that of *Aegicetus gehennae* (B).** Note the exoccipitals projecting laterally (arrows) from the foramen magnum in *G. vogtlensis*, and exoccipitals projecting ventrolaterally (arrows) from the foramen magnum in *A. gehennae*. *A. gehennae* has distinct nuchal flanges above the foramen magnum not found in *G. vogtlensis*. Abbreviations: *Eoc*, exoccipital; *fm*, foramen magnum; *nf*, nuchal flange; *nt*, nuchal tubercle; *oc*, occipital condyle; *Soc*, supraoccipital; *Sq*, squamosal; *Ty*, tympanic.

https://doi.org/10.1371/journal.pone.0225391.g020

one dentary, 39 vertebrae, a caudal chevron, 29 ribs, 7 sternebrae, part of a left and much of a right forelimb, left and right innominates, and much of a left and part of a right hind limb.

*Babiacetus indicus*, *Georgiacetus vogtlensis*, and *Carolinacetus gingerichi* are the three georgiacetines for which a cranium was known previously [2, 31, 34]. That of *Georgiacetus* is the most complete and representative. Comparison of a posterior view of the cranium of *Georgiacetus* with that of *Aegicetus* (Fig 20) illustrates one important way that *Aegicetus* differs from other known georgiacetines. *Georgiacetus*, *Babiacetus*, and *Carolinacetus* all have broad crania with exoccipitals (*Eoc*) projecting laterally from the occipital condyles. *Aegicetus* in contrast has a narrower cranium with exoccipitals projecting ventrolaterally.

*Aegicetus gehennae* as a georgiacetine differs in vertebral formula from all known protocetines. *Aegicetus* has a vertebral formula of 7 cervicals, 15 thoracics, 4 lumbars, 4 sacrals, and 9 + caudals. This is known because the vertebral column of *Aegicetus* was found largely articulated in the field. In contrast, protocetines for which the vertebral column is known, *Rodhocetus kasranii*, *Qaisracetus arifi*, and *Maiacetus inuus*, all have a formula of 7:13:6:4:~21 [1, 3, 5]. *Georgiacetus vogtlensis* has been described as having a vertebral formula of 7:13:8:4:? [2], but this is based on incomplete scatters of vertebrae representing three individuals, with some of the vertebrae poorly preserved [32]. The counts of 13 thoracic and eight lumbar vertebrae have always been questionable. No sacral vertebra of *G. vogtlensis* was found with auricular processes, so the count of four sacrals also seems questionable.

Hulbert [32] recognized a salient feature of *Georgiacetus vogtlensis* to be differential elongation of lumbar, sacral, and caudal vertebrae relative to more anterior vertebrae, which he showed by comparing a length-of-vertebrae profile for *G. vogtlensis* to those for *Protocetus atavus* and *Rodhocetus kasranii*. A similar profile is shown in Fig 21, constructed from measurements of *Aegicetus gehennae* listed in Table 2. This is compared to a vertebral length profile for a complete skeleton of the protocetine *Maiacetus inuus* based on measurements in Gingerich et al. [5]. The vertebral column of *M. inuus* is more complete and better preserved than those of *G. vogtlensis*, *P. atavus*, and *R. kasranii*. The profile in Fig 21 confirms that *A. gehennae* as a representative georgiacetine differs from *M. inuus* as a representative protocetine in having a lumbus, sacrum, and proximal cauda with vertebral centra elongated relative to centra of more anterior vertebrae.

Hulbert [32] also identified an important difference in the innominate of *Georgiacetus vogtlensis* compared to innominates described previously for protocetines. *Georgiacetus* has a relatively smooth medial surface of the iliac blade, with no auricular facet for articulation with an auricular process of the sacrum. No sacral vertebra with auricular processes was recovered with the *Georgiacetus* skeleton, and Hulbert inferred that the skeleton of *Georgiacetus* did not have such a vertebra. *Aegicetus gehennae* is similar to *G. vogtlensis* in having a relatively smooth medial surface of the iliac blade with no auricular facet for articulation with an auricular process of the sacrum, but *A. gehennae* retains a first sacral vertebra with well-developed auricular processes (Fig 10B). The principal purpose of auricular processes on sacral vertebrae is to anchor the innominates and transmit thrust from the hind limbs to the rest of the body. It seems clear that this function was maintained even if we do not know exactly how the innominates were attached to the auricular processes.

Forelimbs and hind limbs of *Aegicetus gehennae* differ in detail from forelimbs and hind limbs of protocetines described to date [4–5, 7], but the most important functional difference in *Aegicetus* appears to be the large size of the manus compared to the pes, or, stating this another way, the small size of the pes compared to the manus (Fig 22). The ratio of Mc-III metacarpal length to Mt-III metatarsal length is 0.94 in *A. gehennae*, and comparable ratios are 0.64, 0.73, and 0.76 for *Rodhocetus balochistanensis* [4–5], *Maiacetus inuus* [5], and *Peregocetus pacificus* [7]. Ratios of Mc-III and Mt-III diameters would tell a similar story.

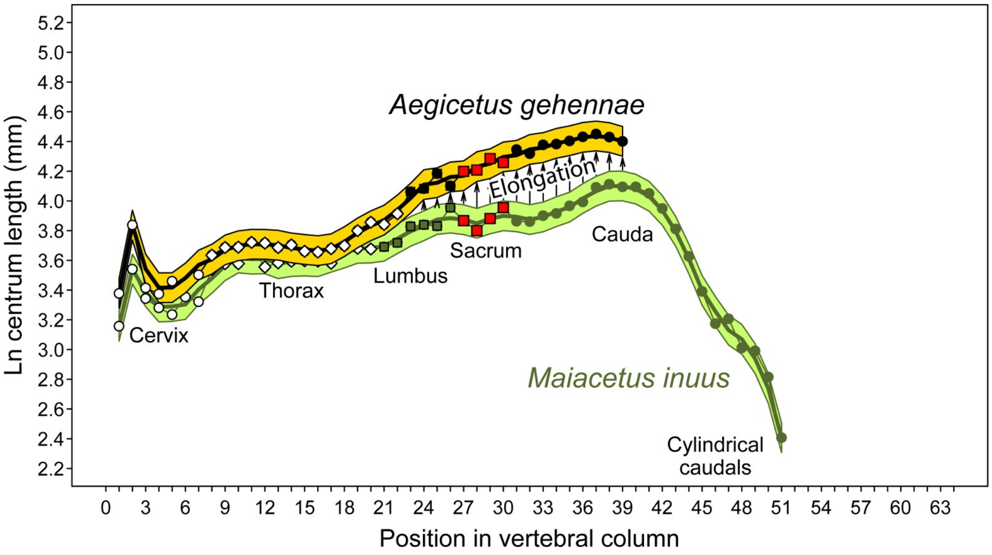


**Fig 21. Vertebral length profile for late Eocene *Aegicetus gehennae* type specimen (CGM 60584; gold) compared to that for early middle Eocene *Maiacetus inuus* (GSP-UM 3551; green).** Vertical axis is proportional, with the natural logarithm of centrum length plotted on the ordinate. Colored bands are inferred 95% confidence intervals for centrum length (3-point running average). *Aegicetus gehennae* is larger. In addition, note that posterior lumbar, sacral, and anterior caudal vertebrae are disproportionately elongated relative to expectation based on the overlap of cervical and thoracic color bands. Posterior caudal vertebrae have not been found for *A*. *gehennae* so it is not known whether they were cylindrical like those of *M*. *inuus* or dorsoventrally flattened like those of contemporary basilosaurids. Symbols: open circles, cervical vertebrae; open diamonds, thoracics; solid squares, lumbars; red squares, sacrals or vertebrae homologous with sacrals; solid circles, caudals.

https://doi.org/10.1371/journal.pone.0225391.g021

*Aegicetus gehennae* differs from earlier protocetids in having long bones of the fore and hind limbs shorter than expected for an animal of its size (Fig 23). Fig 22 shows that robustness of the metapodials, in addition to length, is also quite different in the manus and pes of *A*. *gehennae*. Large forelimbs and large hands are found in fully-aquatic basilosaurids (e.g., *Basilosaurus* [9] and *Dorudon* [8]), where forelimbs and hands function as hydrofoils controlling the

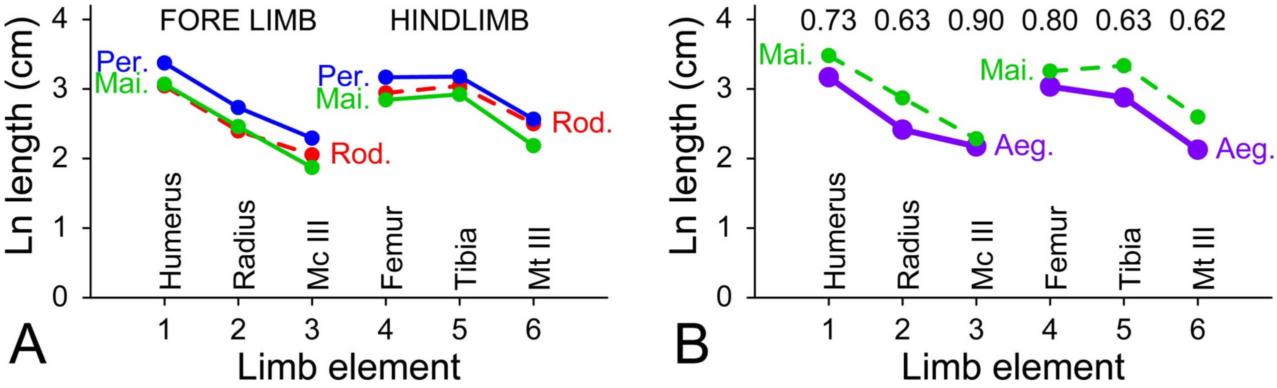


**Fig 23. Forelimbs and hind limbs of the protocetids *Rodhocetus balochistanensis* (*Rod.*) [4], *Maiacetus inuus* (*Mai.*) [5], *Peregocetus pacificus* (*Per.*) [7], and *Aegicetus gehennae* (*Aeg.*) compared in terms of proportion.** A, comparison of *Rodhocetus*, *Maiacetus*, and *Peregocetus*. Note the proportional similarity (parallel trajectories) of *Maiacetus* and *Peregocetus*. *Rodhocetus* has proportionally longer Mc III and Mt III than either *Maiacetus* or *Peregocetus*. B, comparison of *Aegicetus* to a hypothetical *Maiacetus* (or *Peregocetus*) of the same body weight (*Maiacetus*, dashed lines, was rescaled by adding the difference in ln values for the cube-roots of *Aegicetus* and *Maiacetus* body weights). Note that all elements of *Aegicetus* are smaller than expected if it were to be proportioned like *Maiacetus* (by proportions listed across the top of the panel: 0.73, 0.63, etc.). The radius, tibia, and Mt III lengths of *Aegicetus* are the most reduced, and Mc III length of *Aegicetus* is the least reduced.

https://doi.org/10.1371/journal.pone.0225391.g023

direction of movement in water. The same taxa have small hind limbs and small feet, where their function is greatly reduced [8, 49].

There are some interesting angles involved in articulation of the innominate, femur, and tibia-fibula. The long axis of the femur has an angle of about 30˚ relative to a plane circumscribing the articular surface of the femoral head, which suggests that the femur was habitually deflected laterally at about this angle from the sagittal plane. The femoral condyles face more or less posteriorly relative to the long axis of the femur. This is not surprising, but torsion of the femoral shaft means that the articular surface formed by the femoral condyles adds an additional angle of 20–30˚ lateral to that expected from the position of the femoral head and greater trochanter. Thus the tibia too was habitually deflected laterally by an angle approaching 30˚ relative to the sagittal plane. These angles are similar to those observed in the protocetines *Rodhocetus* and *Maiacetus*. They are not angles expected for an animal supporting its weight on land, but may represent compensating angles that stabilize yaw through medial deflection during alternate hind limb paddling.

A difference worth noting for *Aegicetus gehennae* is angulation of the pes relative to the lower leg. In *Rodhocetus balochistanensis* [4], *Maiacetus inuus* [5], and *Peregocetus pacificus* [7] the calcaneum is elongated and the articular surface for the cuboid is more or less opposite the calcaneal tuber. The fibular process of the calcaneum and the facet for articulation of the fibula rise perpendicular to the long axis of the calcaneum [4, 5, 7]. In *A*. *gehennae* the fibular process rises perpendicular to a long axis defined by the calcaneal tuber, but the articular facet for the cuboid is opposite the fibular process and not opposite the calcaneal tuber (Fig 17D). The fibular facet in *Aegicetus* is also shorter and flatter than those of earlier protocetids, indicating, first, that there was much less flexion and extension of the pes relative to the lower leg, and second, that the pes was habitually aligned with the lower leg. The pes of *Aegicetus*, as a representative georgiacetine, could not be flexed the way it was during the recovery stroke of pelvic paddling in protocetines. Limited flexion necessarily limited the efficiency of pelvic paddling in *Aegicetus*. Limited flexion and extension of the pes is also an argument against terrestrial locomotion in *Aegicetus* and georgiacetines.

## Skeletal proportions of *Aegicetus*, other protocetids, and semiaquatic mammals

Principal components analysis provides a way to compare the skeletal proportions of *Aegicetus* and other early whales to those of extant semiaquatic mammals [50]. The latter display a range of sizes, from the Eurasian water shrew *Neomys fodiens* at 15–20 g to the common African hippo *Hippopotamus amphibious* at 1300–1500 kg. This range of sizes is embodied in the range of scores for the first principal component (PC-I in Fig 3, expressing 94% of between-group variance) and the uniformity of PC-I eigenvector coefficients or skeletal-element loadings [50].

Extant semiaquatic mammals also display a range of forms. Semiaquatic mammals that are more terrestrial typically have a long ilium, long femur, and relatively short manual and pedal phalanges. Semiaquatic mammals that are more aquatic, typically have a short ilium, short femur, and relatively long manual and pedal phalanges. These differences are embodied in the range of scores for the second principal component (PC-II in Figs 3–4 of [50], expressing some 4% of between-group variance) and the spectrum of PC-II eigenvector coefficients or skeletal-element loadings.

Some extant semiaquatic mammals have shorter forelimbs and longer hind limbs, and others have longer forelimbs and shorter hind limbs. These differences are embodied in the range of scores for the third principal component (PC-III inFig 4 of [50], expressing about 1% of

between-group variance) and the spectrum of PC-III eigenvector coefficients or skeletal-element loadings.

The contrasts in scores and loadings change slightly when three iconic artiodactyl and archaeocete taxa are added to represent the transition of whales from land to sea. The artiodactyl and archaeocete taxa are: (1) the anthracothere artiodactyl *Elomeryx armatus* in Figs 5–6 of [50]—representing, broadly, terrestrial artiodactyls; (2) the protocetid archaeocete *Rodhocetus kasranii*, combined with *R*. *balochistanensis* in Figs 5–6 of [50]—representing transitional semiaquatic protocetids; and (3) the basilosaurid archaeocete *Dorudon atrox* in Figs 5–6 of [50]—representing fully-aquatic basilosaurids [50]. Fig 6 of [50] is reproduced here, with red lines added to show the inferred transition from an *Elomeryx*-like ancestral artiodactyl to *Rodhocetus* representing early protocetids and then *Dorudon* representing basilosaurids (Fig 24).

In an earlier study *Maiacetus* was shown to be a little more aquatic, substantially more lumbus-dominated, and less hind-limb dominated than *Rodhocetus* [5]. Here we add *Aegicetus gehennae* to assess its skeletal proportions relative the skeletal proportions of extant semi-aquatic mammals and other early whales. All three Eocene protocetids, *Rodhocetus*, *Maiacetus*, and *Aegicetus* cluster near the middle of the extant semiaquatic range. *Rodhocetus* is close to the extant Russian desman *Desmana*, while *Aegicetus* and *Maiacetus* are close to the extant giant river otter *Pteroneura brasiliensis*.

Fig 24 must be interpreted with caution. It represents *Aegicetus* as more terrestrial than *Maiacetus*, which cannot be correct because the pelvis of *Aegicetus* appears to be loosely attached to the sacrum while that of *Maiacetus* is firmly attached. Without a firm connection of the pelvis and hind limbs to the sacrum it is doubtful that *Aegicetus* could support its weight on land. Hence *Aegicetus* was more aquatic than most or all early protocetids. Fig 24 shows *Aegicetus* and *Maiacetus* to be equally balanced in terms of lumbus and hind limb proportions. *Aegicetus* has two fewer lumbar vertebrae than *Maiacetus*, but the hind limb is also reduced some 11% relative to expectation based on *Maiacetus*. Placing *Aegicetus* and *Maiacetus* in the broad framework of *Elomeryx*, *Rodhocetus*, *Dorudon*, plus 50 extant semiaquatic mammals seemingly suppresses some important differences between them.

## Transition from foot-powered to tail-powered swimming

*Aegicetus* is a georgiacetine protocetid. In the description and comparison to *Aegicetus* to protocetine protocetids, outlined above, we found: (1) the lumbar, sacral, and caudal vertebrae of *Aegicetus* are differentially elongated relative to more anterior vertebrae; (2) sacral vertebrae of *Aegicetus* are no longer fused as a rigid platform to support of the hind limbs; (3) the ilia of the innominates of *Aegicetus* lack any direct articulation with auricular processes of the sacrum; (4) the forelimb and manus of *Aegicetus* are enlarged relative to body size; (5) the hind limb and pes of *Aegicetus* are reduced relative to body size; and (6) the pes of *Aegicetus* is constrained to close alignment with the lower leg. The first of these differences suggests that the lumbus and tail of *Aegicetus* and other georgiacetines were more important in locomotion than they were in protocetines. The second, third, fifth, and sixth differences suggest that *Aegicetus*, and probably georgiacetines in general, were less able to support their body weight on land and less able to employ their hind limbs in propulsion. The fourth difference suggests that the forelimb and manus of *Aegicetus* and georgiacetines may have assumed greater importance as a stabilizing hydrofoil.

In a recent review Fish [51] identified five stages of evolution in the locomotor mode of aquatic mammals: (1) quadrupedal paddling; (2) bipedal paddling in the form of alternate pectoral paddling or alternate pelvic paddling; (3) simultaneous pelvic paddling; (4) dorsoventral flexion of the body and tail producing undulatory swimming; and (5) lift-based oscillation of

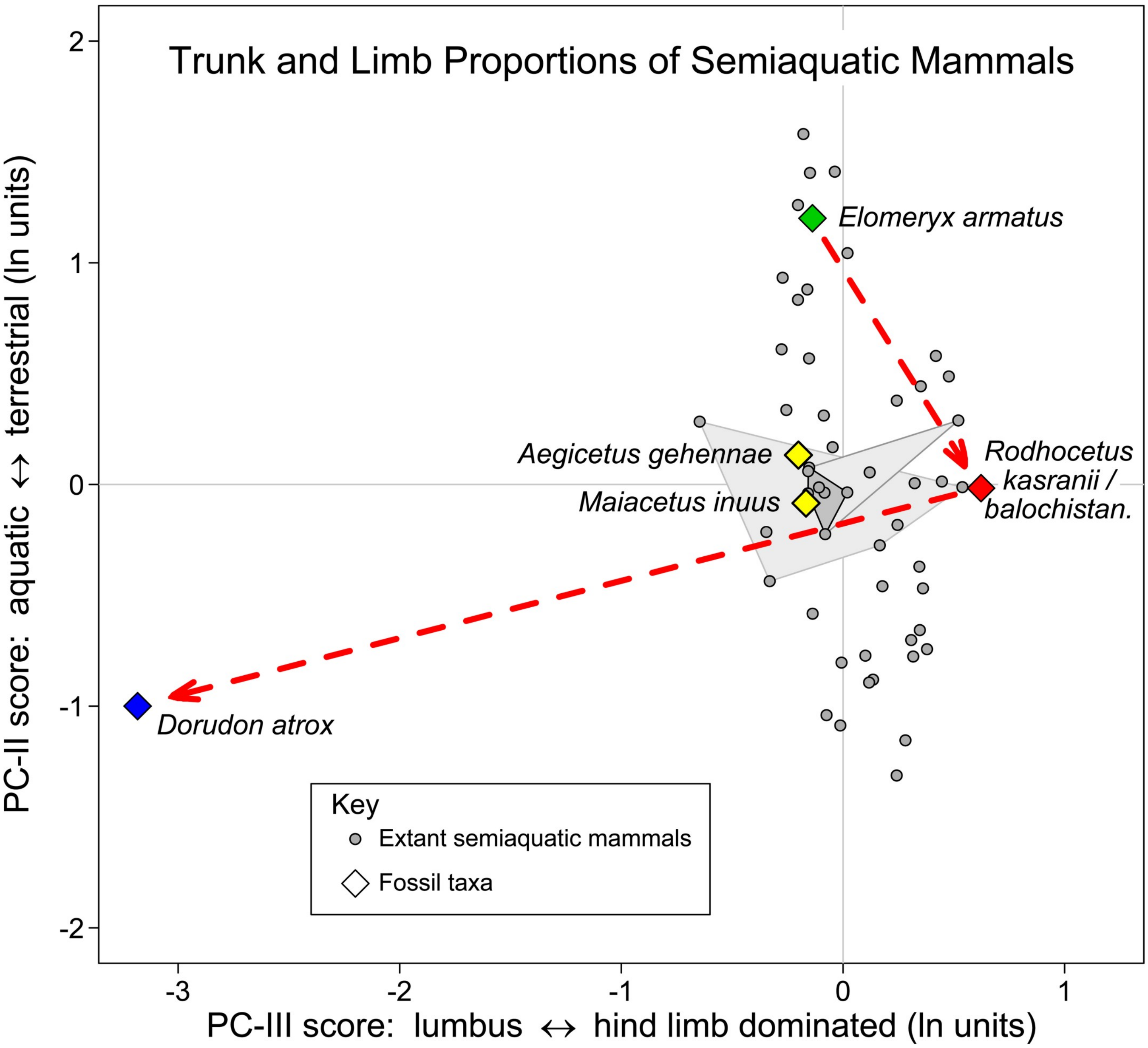


**Fig 24. Transition of whales from land to sea (red arrows).** Principal components plot shows trunk and limb skeletal proportions for the artiodactyl *Elomeryx armatus*, the protocetids *Rodhocetus kasranii / balochistanensis*, the basilosaurid *Dorudon atrox*, and a representative sample of 50 extant semiaquatic mammals. *Aegicetus gehennae* and *Maiacetus inuus* are similarly aquatic to *Rodhocetus* (PC-II) but less specialized as hind-limb swimmers (PC-III). Layered convex polygons (light gray) represent aquatic insectivores, all otters, and river otters, respectively. *Aegicetus* and *Maiacetus* fall near the extant giant otter (*Pteronura brasiliensis*) in this representation of skeletal proportions. Background and comparative data are provided in [50]. Dashed red line shows the generalized path through the morphospace followed during the transition from terrestrial mammals (green diamond) to protocetids (red and yellow diamonds) to basilosaurids (blue diamond). Modern cetaceans are lumbus-dominated and would fall to the left of the chart if they retained all of the skeletal elements measured here.

https://doi.org/10.1371/journal.pone.0225391.g024

the fore limbs, hind limbs, or tail. Fish ([51], p. 1289) indicated that the archaeocete *Ambulocetus* "had limited undulatory swimming ability" and "utilized simultaneous pelvic paddling in concert with spinal undulation similar to otters." Fish ([51], p. p. 1290) interpreted the early protocetid *Rodhocetus* as having an elongated vertebral column for undulation, and associated the basilosaurid *Dorudon* with caudal oscillation.

Fish's summary chart ([51]: Fig 5) outlined a more complete association of archaeocete taxa with stages in the evolution of cetacean swimming. Pakicetids including *Pakicetus* itself were interpreted as bridging quadrupedalism on land with quadrupedal paddling in water. *Ambulocetus* was interpreted as bridging quadrupedal paddling with alternate pelvic paddling. Early protocetids (*Rodhocetus*, *Maiacetus*) were interpreted as bridging alternative pelvic paddling with simultaneous pelvic paddling. Later protocetids (unspecified) were interpreted as bridging simultaneous pelvic paddling with body and tail undulation. Finally, modern whales were interpreted as bridging undulation with caudal oscillation.

A reorganized and more explicit interpretation of Fish's sequence of stages in the evolution of cetacean swimming is outlined here in Table 8. Representative taxa, *Pakicetus*, *Maiacetus*, etc., are described in terms of age, morphological and performance changes from a previous stage, the environment they inhabited, and their mode of locomotion. Note that the 'simultaneous pelvic paddling' stage of locomotor evolution proposed by Fish [51] is missing from Table 8. The unidentified 'later protocetid' that Fish linked to simultaneous pelvic paddling is plausibly represented by *Aegicetus gehennae* described here. However, *Aegicetus* already has the posterior thoracic through caudal elongation associated with mid-body-through-tail undulation. It cannot have been a simultaneous pelvic paddler because its pelvis and hind limbs no longer articulated with the sacrum and vertebral column.

Vertebral length profiles of middle Eocene *Maiacetus inuus* and middle-to-late Eocene *Aegicetus gehennae* previously shown in Fig 21 are compared to profiles for late Eocene *Dorudon atrox* and Recent *Orcinus orca* in Fig 25. Elongation of lumbar, sacral, and caudal vertebrae observed in the transition from a *Maiacetus*-like form to *Aegicetus* continued in the transition from an *Aegicetus*-like form to *Dorudon*. *Basilosaurus* had even greater vertebral elongation [9, 49]. *Dorudon* has elongated posterior thoracic, lumbar, sacral, and caudal vertebrae, indicating that propulsion in *Dorudon*, like that in *Basilosaurus* [52], was probably provided in part by mid-body-through-tail undulation. The profile for *Orcinus* illustrates the reduction in number of vertebrae and shortening of the torso characteristic of modern whales propelled by caudal oscillation with a tail fluke [53].

*Dorudon atrox* has flattened caudal vertebrae at the end of its tail, which Uhen [8] interpreted as evidence of a tail fluke. However, the flattened caudals of *Dorudon* occupy a smaller proportion of vertebral column length (ca. 4.1%) than the flattened caudals associated with a fluke in modern whales (Fig 26). Flattened terminal caudal vertebrae range from ca. 5.5% to 10.5% of vertebral column length (mean 7.5%) in a large sample of modern whales ($N = 36$). *Basilosaurus isis*, like *D. atrox*, has flattened caudal vertebrae at the end of its tail, but in *Basilosaurus* these occupy an even smaller proportion of vertebral column length (ca. 3.1%). It is questionable whether such short flukes on the bodies of long archaeocetes could provide enough surface area to power a large volume and mass through water [54].

Buchholtz [52] wrote of *Basilosaurus* "the plateau of dimensionally uniform elongate vertebrae signals the presence of an undulatory wave of constant amplitude," which "supports interpretation of an anguilliform swimming pattern with a dorsoventral orientation" (Buchholtz p. 346). Further, she wrote "successive undulatory waves of the long torso must have generated most of the propulsive force." Flattening of a short segment at the end of a long body like that of *Basilosaurus* may have evolved as a stabilizer or to enhance maneuverability rather than provide propulsive power. *Dorudon* has shorter vertebrae than those of *Basilosaurus* but *Dorudon* conceivably represents a similar locomotor pattern. *Dorudon* is more like later Neoceti, modern whales, in having shorter vertebrae and a proportionally slightly longer area of flattened caudals.

Bebej and Smith [55] studied the lumbar vertebrae of early whales and found that the lumbar portion of the vertebral column was relatively stable in the earliest archaeocetes (*Pakicetus*,

**Table 8. Summary of changes in morphology, performance, environment, and locomotor mode during the Eocene evolution of cetacea.**

| Family Group | Representative Taxon | Age | Morphological Changes | Performance Changes | Environment | Predominant Locomotor Mode |
|---|---|---|---|---|---|---|
| Delphinidae | ***Orcinus orca*** | Recent | Shortened compact body | Lift-based propulsion | Fully aquatic | Constrained dorsoventral |
| | | | Development of tail fluke | | | caudal oscillation |
| | | | Loss of external hind limbs | | | |
| Basilosauridae | ***Dorudon atrox*** | 36 Ma | Elongated lumbus and tail | Reduced drag | Fully aquatic | Mid-body through tail |
| | | | Reduced hind limbs | Increased efficiency | | dorsoventral and lateral (?) |
| | | | Flattened tail at tip | | | undulation |
| Protocetidae | ***Aegicetus gehennae*** | 38 Ma | Elongated lumbus and tail | Reduced drag | Fully aquatic | Mid-body through tail |
| (Georgiacetinae) | | | Enlarged forelimbs | Increased efficiency | | dorsoventral and lateral (?) |
| | | | Loss of pelvic articulation | | | undulation |
| Protocetidae | ***Maiacetus inuus*** | 46 Ma | Enlarged hind feet | Drag-based propulsion | Semiaquatic | Alternate pelvic paddling with |
| (Protocetinae) | | | Interdigital webbing | | | caudal stabilization |
| | | | Tail as inertial stabilizer | | | |
| Pakicetidae | ***Pakicetus attocki*** | 48 Ma | Larger body size | Drag-based propulsion | Semiaquatic | Quadrupedal paddling with |
| | | | Elongated phalanges | | | caudal stabilization |
| | | | Interdigital webbing | | 7 | |
| Dichobunidae | ***Diacodexis ilicis*** | 56 Ma | Small body size | Ricochetal cursor | Terrestrial | Terrestrial quadruped |
| (Diacodexeinae) | | | Elongated metapodials | | | with dorsoventral |
| | | | Hoofed terminal phalanges | | | flexion and extension |

https://doi.org/10.1371/journal.pone.0225391.t008

*Ambulocetus*, *Remingtonocetus*), with a significant increase in mobility appearing in early protocetids (*Rodhocetus*, *Maiacetus*). More advanced protocetids (*Qaisracetus*, *Georgiacetus*) were

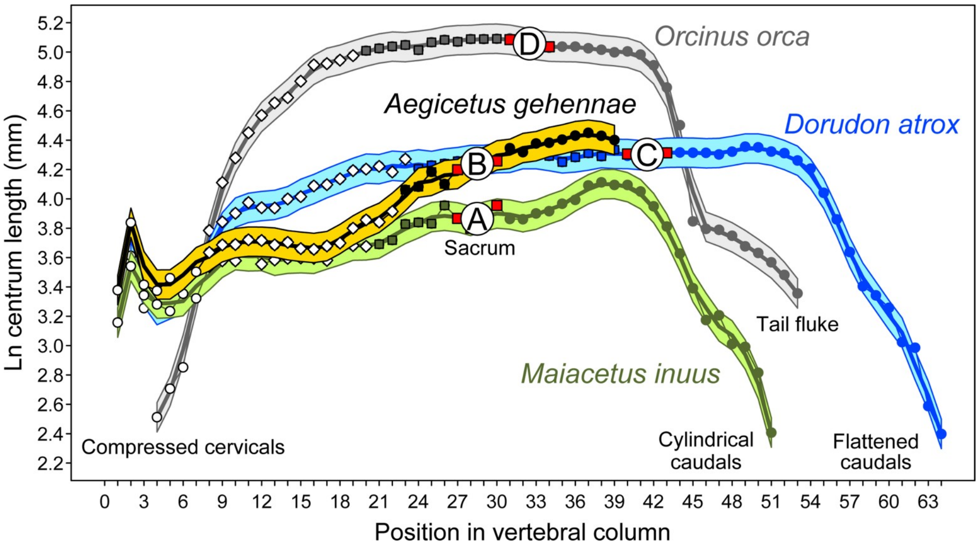


**Fig 25. Vertebral length profiles for three stages of archaeocete evolution leading to modern whales.** A, early middle Eocene protocetid *Maiacetus inuus*, a foot-powered swimmer (GSP-UM 3551; green). B, late Eocene protocetid *Aegicetus gehennae*, a transitional undulatory swimmer with an elongated body (CGM 60584; gold). C, late Eocene basilosaurid *Dorudon atrox*, an undulatory swimmer with an elongated body (UM 101215, etc.; blue). D, modern *Orcinus orca*, an oscillatory tail-powered swimmer (AMNH 34276; gray). Transition from A to B involved elongation of posterior lumbar through anterior caudal vertebrae (Fig 21), reduction of the sacroiliac articulation between the vertebral column and innominate, and loss of fusion between sacral vertebrae. Transition from B to C involved addition of mid-body vertebrae and lumbarization of sacral vertebrae. Transition from C to D involved shortening by reduction in the number of mid-body vertebrae, or, in some cetaceans, shortening of the lengths of individual vertebrae. Symbols are explained in Fig 21.

https://doi.org/10.1371/journal.pone.0225391.g025

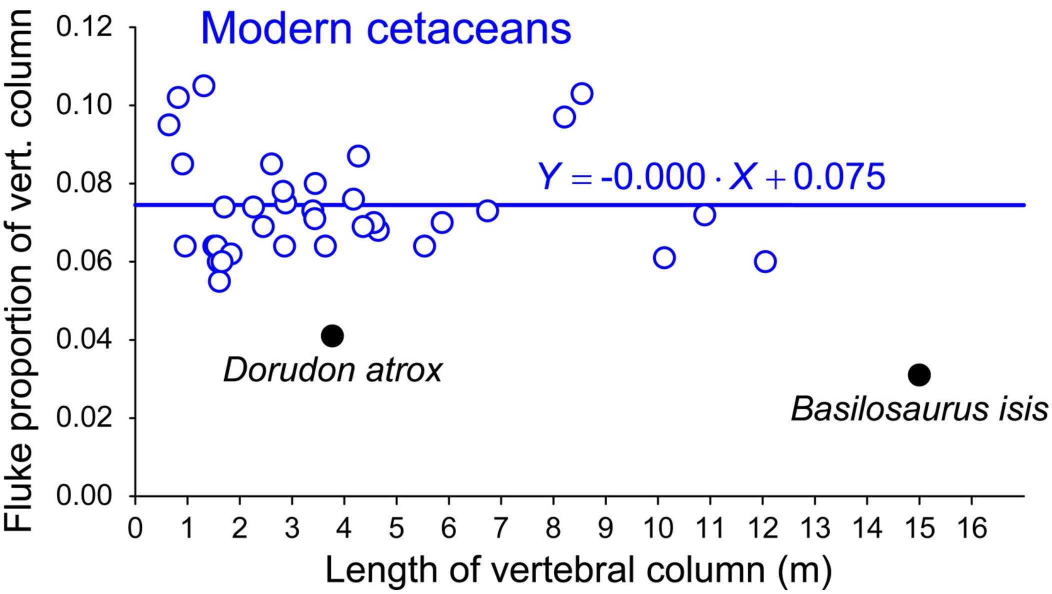


**Fig 26. Fluke vertebral length as a proportion of total vertebral column length in cetaceans.** Modern species are shown in blue (21 species, $N = 36$ individuals). Fluke proportions are regressed on their denominators, showing that fluke proportions are independent of vertebral column length. *Dorudon atrox* has a fluke proportion of 0.041 (4.1%), and *Basilosaurus isis* has a fluke proportion of 0.031 (3.1%). Both are significantly smaller than the fluke proportion for any modern cetacean. Thus it is questionable whether *Dorudon* and *Basilosaurus* were tail-powered swimmers. A small fluke on a relatively long body may have served, initially, as a caudal stabilizer.

https://doi.org/10.1371/journal.pone.0225391.g026

found to have more mobile lumbar columns, and basilosaurids (*Dorudon*, *Basilosaurus*) were found to have the most mobile lumbar columns of any archaeocete. Lumbar-sacral-caudal undulation was inferred to generate propulsion during swimming. Finally, as swimming was increasingly refined in later cetaceans powered by a tail fluke, lumbar mobility was again largely lost as mobility was restricted to the tail. *Aegicetus*, as a close relative of *Georgiacetus*, appears to fit perfectly into this history.

The transition from foot-powered swimming to tail-powered swimming has long been an enigma in cetacean evolution. Some authors (e.g., Fish [51] and references therein) envisioned otter-like simultaneous-pelvic-paddling in concert with spinal undulation to be the key. This would enable, first, conversion of drag-based propulsion into lift-based propulsion, which could then, secondarily, be transferred step-by-step from a foot-formed hydrofoil to a tail-formed hydrofoil. However, decoupling of the pelvis and pelvic limbs from the vertebral column in concert with posterior thoracic, lumbar, sacral, and caudal vertebral elongation, documented by Hulbert [2, 32] for *Georgiacetus* and here for *Aegicetus*, provides contradictory evidence. Elongation of the vertebral column supports an alternative hypothesis.

Elongation of posterior thoracic, lumbar, sacral, and anterior caudal vertebrae beyond that seen in early protocetids would decrease the efficiency of pelvic paddling and increase the potential for undulation. The well-muscled lumbus and tail required for lateral stabilization during pelvic paddling in early protocetids is plausibly a preadaptation for swimming with components of lateral as well as dorsoventral undulation. The first fully aquatic cetaceans could no longer come out on land to rest, and resting may have taken place on the horizontal sea surface where, with their elongated bodies, it is possible that lateral undulation was advantageous.

Following Buchholtz [52] and Bebej and Smith [55], vertebral elongation in late fully-aquatic protocetids such as *Aegicetus* is interpreted to facilitate undulation. Underwater

swimming was probably powered by some combination of dorsoventral and lateral undulation. Basilosaurids with elongated vertebral columns, such as *Basilosaurus isis* and *B. cetoides*, almost certainly swam using a combination of dorsoventral and lateral undulation. These species show the greatest elongation [9, 49] and possibly the greatest undulatory swimming efficiency.

The idea of an intermediate elongated-body undulatory or anguilliform swimming stage in the transition of whales from foot-powered swimming to tail-powered swimming has independent parallels in the early evolution of both ichthyosaurs [56] and mosasaurs [57]. Aquatic propulsion in crocodiles is a model for *Aegicetus*-like caudal undulation, and their kinematic efficiency is considered to be greater than that of semi-aquatic mammals and comparable to the efficiency of fully aquatic mammals [58].

The final step in the transition to oscillatory swimming in modern whales such as *Orcinus orca* involved shortening of the vertebral column by reduction in the number of mid-body vertebrae (Fig 25; [59], and in many cases shortening of individual vertebrae). Steady swimming in cetaceans led to dorsoventral oscillation [60], and a compact body with a shorter tail increased both the leverage and power delivered to a horizontal fluke [53–55, 59–60].

## Conclusions

*Aegicetus gehennae*, new genus and species, from Egypt is represented by the most complete skeleton of a georgiacetine protocetid known to date. Two specimens are known, plausibly sexually dimorphic, and the holotype is interpreted as male. *A. gehennae* was large for a protocetid, with a male body weight estimated at 890 kg. It has a distinctive configuration of occipital bones, and an unusual vertebral count of 15 thoracic vertebrae and only 4 lumbars. The forelimbs and hind limbs are relatively small in proportion to body size when compared to those of earlier protocetids. Within the skeleton of *Aegicetus*, the forelimb and manus are relatively large and robust when compared to the hind limb and pes.

*Aegicetus gehennae* from the earliest Priabonian (earliest late Eocene) is the latest-surviving protocetid known to date. It groups with known protocetids in a quantified spectrum of semi-aquatic mammalian skeletal proportions. However, *A. gehennae*, like *Georgiacetus vogtlensis*, differs in lacking a firm connection of pelvic limbs to the vertebral column: thus it was fully aquatic. *A. gehennae* has the elongated posterior thoracic, lumbar, sacral, and anterior caudal vertebrae first reported for *G. vogtlensis*, and it appears that georgiacetine protocetids, like some later basilosaurids, swam by mid-body-through-tail undulation. It is plausible to consider some combination of dorsoventral and lateral undulation to be a locomotor mode in the transition from foot-powered swimming in early protocetids to the more constrained dorsoventral caudal oscillation of modern whales.

## Supporting information

**S1 Table. Online hyperlinks for three-dimensional images of protocetid *Aegicetus gehennae* skeletal elements (CGM 60584).**
(PDF)

## Acknowledgments

We thank Mohammad Talaat Al Hennawy, Manager, Egyptian Environmental Affairs Agency, for access to the Wadi Al Hitan and Wadi Al Rayan Protected Areas and for encouragement of this research. Park rangers in both areas provided helpful assistance in the field. William J. Sanders and John F. Graf did the preparation, molding, and casting of CGM 60583 and 60584.

Emily Buchholtz provided vertebral measurements for many of the modern whales included in Fig 26. Adam Rountrey helped with curation and together with Marissa Clarke produced the online photogrammetric images. We thank Olivier Lambert and two anonymous reviewers for constructive comments that improved the manuscript substantially.

Research in Egypt was sponsored by the Egyptian Geological Museum, the Egyptian Environmental Affairs Agency, the Egyptian Mineral Resources Authority, the University of Michigan Museum of Paleontology, the U. S. National Science Foundation (NSF OISE0513544, EAR-0920972), and the National Geographic Society (NGS 7226–04).

## Author Contributions

**Conceptualization:** Philip D. Gingerich, Mohammed Sameh M. Antar, Iyad S. Zalmout.

**Data curation:** Philip D. Gingerich, Mohammed Sameh M. Antar, Iyad S. Zalmout.

**Formal analysis:** Philip D. Gingerich, Mohammed Sameh M. Antar, Iyad S. Zalmout.

**Funding acquisition:** Philip D. Gingerich.

**Investigation:** Philip D. Gingerich, Mohammed Sameh M. Antar, Iyad S. Zalmout.

**Methodology:** Philip D. Gingerich, Mohammed Sameh M. Antar, Iyad S. Zalmout.

**Project administration:** Philip D. Gingerich, Mohammed Sameh M. Antar, Iyad S. Zalmout.

**Resources:** Philip D. Gingerich, Mohammed Sameh M. Antar, Iyad S. Zalmout.

**Software:** Philip D. Gingerich.

**Visualization:** Philip D. Gingerich.

**Writing – original draft:** Philip D. Gingerich.

**Writing – review & editing:** Philip D. Gingerich, Mohammed Sameh M. Antar, Iyad S. Zalmout.

## References

1. Gingerich PD, Raza SM, Arif M, Anwar M, Zhou X. New whale from the Eocene of Pakistan and the origin of cetacean swimming. Nature. 1994; 368: 844–847. https://doi.org/10.1038/368844a0
2. Hulbert RC, Petkewich RM, Bishop GA, Bukry D, Aleshire DP. A new middle Eocene protocetid whale (Mammalia: Cetacea: Archaeoceti) and associated biota from Georgia. J Paleontol. 1998; 72: 907–927. https://doi.org/10.1017/S0022336000027232
3. Gingerich PD, Haq Mu, Khan IH, Zalmout IS. Eocene stratigraphy and archaeocete whales (Mammalia, Cetacea) of Drug Lahar in the eastern Sulaiman Range, Balochistan (Pakistan). Contributions from the Museum of Paleontology, University of Michigan. 2001; 30: 269–319.
4. Gingerich PD, Haq Mu, Zalmout IS, Khan IH, Malkani MS. Origin of whales from early artiodactyls: hands and feet of Eocene Protocetidae from Pakistan. Science. 2001; 293: 2239–2242. https://doi.org/10.1126/science.1063902 PMID: 11567134
5. Gingerich PD, Haq Mu, Koenigswald Wv, Sanders WJ, Smith BH, Zalmout IS. New protocetid whale from the middle Eocene of Pakistan: birth on land, precocial development, and sexual dimorphism. PLoS One. 2009; 4 (e4366): 1–20. https://doi.org/10.1371/journal.pone.0004366 PMID: 19194487
6. Uhen MD. New material of *Natchitochia jonesi* and a comparison of the innominata and locomotor capabilities of Protocetidae. Marine Mammal Science. 2014; 30: 1029–1066. https://doi.org/10.1111/mms.12100
7. Lambert O, Bianucci G, Salas-Gismondi R, Di Celma C, Steurbaut E, Urbina M, et al. An amphibious whale from the middle Eocene of Peru reveals early South Pacific dispersal of quadrupedal cetaceans. Curr Biol. 2019; 29: 1352–1359. https://doi.org/10.1016/j.cub.2019.02.050 PMID: 30955933
8. Uhen MD. Form, function, and anatomy of *Dorudon atrox* (Mammalia, Cetacea): an archaeocete from the middle to late Eocene of Egypt. University of Michigan Papers on Paleontology. 2004; 34: 1–222.

9. Gingerich PD, Smith BH, Simons EL. Hind limbs of Eocene *Basilosaurus isis*: evidence of feet in whales. Science. 1990; 249: 154–157. https://doi.org/10.1126/science.249.4965.154 PMID: 17836967
10. Voss M, Antar MSM, Zalmout IS, Gingerich PD. Stomach contents of the archaeocete *Basilosaurus*: apex predator in oceans of the late Eocene. PLoS One. 2019; 14: e0209021. https://doi.org/10.1371/journal.pone.0209021 PMID: 30625131
11. Zalmout IS, Gingerich PD. Late Eocene sea cows (Mammalia, Sirenia) from Wadi Al Hitan in the Western Desert of Fayum, Egypt. University of Michigan Papers on Paleontology. 2012; 37: 1–158.
12. Iskander F. Geological survey of the Gharaq el Sultani sheet no. 68/54. Standard Oil Company, Egypt S. A., Reports. 1943; 57: 1–29.
13. Beadnell HJL. The topography and geology of the Fayum Province of Egypt. Cairo: Survey Department of Egypt; 1905.
14. Said R. The Geology of Egypt. Amsterdam: Elsevier; 1962.
15. Bu Haq, Hardenbol JA Vail PR. Chronology of fluctuating sea levels since the Triassic. Science. 1987; 235: 1156–1167. https://doi.org/10.1126/science.235.4793.1156 PMID: 17818978
16. Hardenbol JA, Thierry J, Farley MB, Jacquin T, Graciansky P-Cd, Vail PR. Mesozoic and Cenozoic sequence chronostratigraphic framework of European basins. In: Graciansky P-Cd, Hardenbol JA, Jacquin T, Vail PR, editors. Mesozoic and Cenozoic sequence stratigraphy of European basins: SEPM Society for Sedimentary Geology, Special Publication 60; 1998: p. 3–13 (eight charts).
17. Gingerich PD, Zalmout IS, Antar MSM, Williams EM, Carlson AE, Kelly DC, et al. Large-scale glaciation and deglaciation of Antarctica during the late Eocene: reply. Geology. 2012; 40: e255. https://doi.org/10.1130/G33046Y.1
18. Strougo A, Faris M, Haggag MAY, Abul-Nasr RA, Gingerich PD. Planktonic foraminifera and calcareous nannofossil biostratigraphy through the middle-to-late Eocene transition at Wadi Hitan, Fayum Province, Egypt. Contributions from the Museum of Paleontology, University of Michigan. 2013; 32: 111–138.
19. Agnini C, Fornaciari E, Giusberti L, Grandesso P, Lanci L, Luciani V, et al. Integrated biomagnetostratigraphy of the Alano section (NE Italy): a proposal for defining the middle-late Eocene boundary. Geol Soc Am Bull. 2011; 123: 841–872. https://doi.org/10.1130/B30158.1
20. Linnaeus C. Systema Naturae, tenth edition. Holmiae (Stockholm): Laurentii Salvii; 1758.
21. Brisson M-J. Regnum Animale in Classes IX. Distributum, sive Synopsis Methodica. Leiden: Lugduni Batavorum, Theodorum Haak; 1762.
22. Flower WH. On the arrangement of the orders and families of existing Mammalia. Proceedings of the Zoological Society of London. 1883; 1883: 178–186.
23. Stromer von Reichenbach E. Die Archaeoceti des ägyptischen Eozäns. Beiträge zur Paläontologie und Geologie Österreich-Ungarns und des Orients, Vienna. 1908; 21: 106–178.
24. Gingerich PD, Zalmout IS, Haq M, Bhatti MA. *Makaracetus bidens*, a new protocetid archaeocete (Mammalia, Cetacea) from the early middle Eocene of Balochistan (Pakistan). Contributions from the Museum of Paleontology, University of Michigan. 2005; 31: 197–210.
25. McLeod SA and Barnes LG. A new genus and species of Eocene protocetid archaeocete whale (Mammalia, Cetacea) from the Atlantic Coastal Plain. In Wang X and Barnes LG, editors. Geology and vertebrate paleontology of western and southern North America: contributions in honor of David P. Whistler. Natural History Museum of Los Angeles County Science Series. 2008; 41: 73–98.
26. Andrews CW. A description of new species of zeuglodont and of leathery turtle from the Eocene of southern Nigeria. Proceedings of the Zoological Society of London. 1920; 89: 309–319. https://doi.org/10.1111/j.1096-3642.1919.tb02124.x
27. Halstead LB, Middleton JA. New material of the archaeocete whale, *Pappocetus lugardi* Andrews, from the middle Eocene of Nigeria. Journal of Mining and Geology. 1974; 8: 81–85.
28. Halstead LB, Middleton JA. Fossil vertebrates of Nigeria. Part II, 3.4, Archaeocete whale: Pappocetus lugardi Andrews, 1920. The Nigerian Field. 1976; 41: 131–133.
29. Gingerich PD, Zouhri S. New fauna of archaeocete whales (Mammalia, Cetacea) from the Bartonian middle Eocene of southern Morocco. J Afr Earth Sci. 2015; 111: 273–286. https://doi.org/10.1016/j.jafrearsci.2015.08.006
30. Trivedy AN, Satsangi PP. A new archaeocete (whale) from the Eocene of India. Abstracts of 27th International Geological Congress, Moscow. 1984; 1: 322–323.
31. Gingerich PD, Arif M, Bhatti MA, Raza HA, Raza SM. *Protosiren* and *Babiacetus* (Mammalia, Sirenia and Cetacea) from the middle Eocene Drazinda Formation, Sulaiman Range, Punjab (Pakistan). Contributions from the Museum of Paleontology, University of Michigan. 1995; 29: 331–357.

32. Hulbert RC. Postcranial osteology of the North American middle Eocene protocetid *Georgiacetus*. In: Thewissen JGM, editor. The emergence of whales: evolutionary patterns in the origin of Cetacea. New York: Plenum Press; 1998. pp. 235–267.
33. Uhen MD. New protocetid (Mammalia, Cetacea) from the late middle Eocene Cook Mountain Formation of Louisiana. J Vertebr Paleontol. 1998; 18: 664–668.
34. Geisler JH, Sanders AE, Luo Z. A new protocetid whale (Cetacea: Archaeoceti) from the late middle Eocene of South Carolina. American Museum Novitates. 2005; 3480: 1–65.
35. Bianucci G, Gingerich PD. *Aegyptocetus tarfa* n. gen. et sp. (Mammalia, Cetacea) from the middle Eocene of Egypt: clinorhynchy, olfaction, and hearing in a protocetid whale. J Vertebr Paleontol. 2011; 31: 1173–1188. https://doi.org/10.1080/02724634.2011.607985
36. Bromley RG, Martinell J. *Centrichnus*, new ichnogenus for centncally pattemed attachment scars on skeletal substrates. Bulletin of the Geological Society of Denmark. 1991; 38: 243–252.
37. Santos A, Mayoral EJ, Guinea FM. Bioerosion scars of acorn barnacles from the south-western Iberian Peninsula, upper Neogene. Rivista Italiana di Paleontologia e Stratigrafia. 2005; 111: 181–189. https://doi.org/10.13130/2039-4942/6289
38. Montie EW, Manire CA, Mann DA. Live CT imaging of sound reception anatomy and hearing measurements in the pygmy killer whale, *Feresa attenuata*. J Exp Biol. 2011; 214: 945–955. https://doi.org/10.1242/jeb.051599 PMID: 21346122
39. Luo Z, Gingerich PD. Terrestrial Mesonychia to aquatic Cetacea: transformation of the basicranium and evolution of hearing in whales. University of Michigan Papers on Paleontology. 1999; 31: 1–98.
40. Mourlam MJ, Orliac MJ. Protocetid (Cetacea, Artiodactyla) bullae and petrosals from the middle Eocene locality of Kpogamé, Togo: new insights into the early history of cetacean hearing. J Syst Palaeontol. 2017;1–24. https://doi.org/10.1080/14772019.2017.1328378
41. Reidenberg JS, Laitman JT. Anatomy of the hyoid apparatus in Odontoceti (toothed whales): specializations of their skeleton and musculature compared with those of terrestrial mammals. Anat Rec. 1994; 240: 598–624. https://doi.org/10.1002/ar.1092400417 PMID: 7879911
42. Flower WH, Gadow H. An Introduction to the Osteology of the Mammalia, Third Edition. London: MacMillan. 1885.
43. Gingerich PD. Body weight and relative brain size (encephalization) in Eocene Archaeoceti (Cetacea). J Mamm Evol. 2015; 23: 17–31. https://doi.org/10.1007/s10914-015-9304-y
44. Fraas E. Neue Zeuglodonten aus dem unteren Mitteleocän vom Mokattam bei Cairo. Geologische und Paläontologische Abhandlungen, Neue Folge, Jena. 1904; 6: 197–220.
45. Sahni A, Mishra VP. Lower Tertiary vertebrates from western India. Palaeontological Society of India, Monographs. 1975; 3: 1–48.
46. Gingerich PD, Arif M, Clyde WC. New archaeocetes (Mammalia, Cetacea) from the middle Eocene Domanda Formation of the Sulaiman Range, Punjab (Pakistan). Contributions from the Museum of Paleontology, University of Michigan. 1995; 29: 291–330.
47. Bajpai S, Thewissen JGM. Protocetid cetaceans (Mammalia) from the Eocene of India. Palaeontol Electronica. 2014; 17: 1–19.
48. Gibson ML, Mnieckowski J, Geisler JH. *Tupelocetus palmeri*, a new species of protocetid whale (Mammalia, Cetacea) from the middle Eocene of South Carolina. J Vertebr Paleontol. 2019;e1555165. https://doi.org/10.1080/02724634.2018.1555165
49. Kellogg R. A review of the Archaeoceti. Carnegie Institution of Washington, Publications. 1936; 482: 1–366.
50. Gingerich PD. Land-to-sea transition of early whales: evolution of Eocene Archaeoceti (Cetacea) in relation to skeletal proportions and locomotion of living semiaquatic mammals. Paleobiology. 2003; 29: 429–454. https://doi.org/10.1666/0094-8373(2003)029<0429:ltiewe>2.0.CO;2
51. Fish FE. Secondary evolution of aquatic propulsion in higher vertebrates: validation and prospect. Integr Comp Biol. 2016; 56: 1285–1297. https://doi.org/10.1093/icb/icw123 PMID: 27697779
52. Buchholtz EA. Implications of vertebral morphology for locomotor evolution in early Cetacea. In: Thewissen JGM, editor. The emergence of whales: evolutionary patterns in the origin of Cetacea. New York: Plenum Press; 199pp. 325–351.
53. Buchholtz EA, Schur SA. Vertebral osteology in Delphinidae (Cetacea). Zool J Linn Soc. 2004; 140: 383–401. https://doi.org/10.1111/j.1096-3642.2003.00105.x
54. Webb PW, Buffrénil V. Locomotion in the biology of large aquatic vertebrates. Trans Am Fish Soc. 1990; 119: 629–641. https://doi.org/10.1577/1548-8659(1990)119<0629:LITBOL>2.3.CO;2

55. Bebej RM, Smith KM. Lumbar mobility in archaeocetes (Mammalia: Cetacea) and the evolution of aquatic locomotion in the earliest whales. Zool J Linn Soc. 2017; 182: 695–721. https://doi.org/10.1093/zoolinnean/zlx058

56. Motani R, You H, McGowan C. Eel-like swimming in the earliest ichthyosaurs. Nature. 1996; 382: 347–348. https://doi.org/10.1038/382347a0

57. Lindgren J, Polcyn MJ, Young BA. Landlubbers to leviathans: evolution of swimming in mosasaurine mosasaurs. Paleobiology. 2011; 37: 445–469. https://doi.org/10.1666/09023.1

58. Seebacher F, Elsworth PG, Franklin CE. Ontogenetic changes of swimming kinematics in a semi-aquatic reptile (*Crocodylus porosus*). Aust J Zool. 2003; 51: 15–24. https://doi.org/10.1071/ZO02036

59. Buchholtz EA. Vertebral osteology and swimming style in living and fossil whales (Order: Cetacea). J Zool. 2001; 253: 175–190. https://doi.org/10.1017/S0952836901000164

60. Pabst DA. To bend a dolphin: convergence of force transmission designs in cetaceans and scombrid fishes. Am Zool. 2000; 40: 146–155. https://doi.org/10.1093/icb/40.1.146
